# Molecular inter-kingdom interactions of endophytes isolated from *Lychnophora ericoides*

**DOI:** 10.1038/s41598-017-05532-5

**Published:** 2017-07-14

**Authors:** Andrés M. Caraballo-Rodríguez, Pieter C. Dorrestein, Monica T. Pupo

**Affiliations:** 1 Faculdade de Ciências Farmacêuticas de Ribeirão Preto, Universidade de São Paulo, Ribeirão Preto, SP 14040-903 Brazil; 2 Collaborative Mass Spectrometry Innovation Center, Skaggs School of Pharmacy and Pharmaceutical Sciences, University of California - San Diego, La Jolla, CA 92093 USA

## Abstract

The importance of microbial natural products has been widely demonstrated in the search for new antibiotics. However, the functional role of microbial metabolites in nature remains to be deciphered. Several natural products are known to mediate microbial interactions through metabolic exchange. One approach to investigate metabolic exchange in the laboratory is through microbial interactions. Here, we describe the chemical study of selected endophytes isolated from the Brazilian medicinal plant *Lychnophora ericoides* by pairwise inter-kingdom interactions in order to correlate the impact of co-cultivation to their metabolic profiles. Combining mass spectrometry tools and NMR analyses, a total of 29 compounds were identified. These compounds are members of polyene macrocycles, pyrroloindole alkaloids, angucyclines, and leupeptins chemical families. Two of the identified compounds correspond to a new fungal metabolite (**29**) and a new actinobacterial angucycline-derivative (**23**). Our results revealed a substantial arsenal of small molecules induced by microbial interactions, as we begin to unravel the complexity of microbial interactions associated with endophytic systems.

## Introduction

Endophytes, reported for the first time more than a century ago^1^, are described as microorganisms inhabiting plant tissues without causing pathogenic effects into their host^2, 3^. These microorganisms are part of complex systems of balanced interactions mediated by natural products between endophytes and their host^4–6^. The biotechnological interest in endophytic microbes due to their potential as sources of natural products began with the discovery of paclitaxel^7^, and subsequent reports of other important bioactive molecules from endophytes, such as deoxypodophyllotoxin^8^, maytansine^9, 10^, campthotecin^11, 12^, among many others^13^.

In the recent years, the investigation of natural products from microorganisms by using co-culture strategies has increased^14–16^. The co-culture approach is one of the several other strategies to induce the expression of cryptic microbial metabolites^17^, and increase metabolic diversity^15, 18, 19^. Since microorganisms are part of natural consortia^6^, the discovery of natural products from microbial interactions, not present in axenic cultures, is expected. Studies have demonstrated the elicitation of microbial metabolites as a consequence of interactions from endophytes^20, 21^, including overproduction of paclitaxel^22^. Following this rational, it is a promising endeavor to study the chemistry of interacting microorganisms to reveal molecules that are instrumental for the establishment of complex communities^23^. Studies into the chemistry of endophytes, combined with investigation of their involvement in microbial interactions, would contribute to our understanding of their role in natural environments^24, 25^.

The co-culture strategy, combined with mass spectrometry (MS) approaches, such as molecular networking^26–28^, and automatic search against experimental and *in silico* databases^29^, can lead to the detection and identification of microbial metabolites. MS-based molecular networking uses tandem MS (MS/MS) to correlate chemical entities by structural similarity, identify known molecules and accelerate the discovery of new ones^27, 30^. However, the challenge remains in the correct identification of most of these chemical entities^31^. Therefore, the fully characterization by Nuclear Magnetic Resonance (NMR) and MS of purified compounds is pivotal to confirm chemical structures^27, 30^.

Several studies investigating natural products from the Brazilian medicinal plant *Lychnophora ericoides* have been published^32–43^. Due to the importance of this plant in folk medicine^42, 43^, and the interesting biosynthetic potential of endophytic microorganisms^13^, we initiated the investigation of natural products from endophytes of *L*. *ericoides* ^44^. A total of sixteen compounds were isolated from endophytic actinobacteria of *L*. *ericoides*, revealing these microorganisms as a promising source of natural products^44^. This potential was also demonstrated in a recent study where we investigated the chemical arsenal produced by one of these endophytic actinobacteria strains, *Streptomyces albospinus* RLe7^45^. A total of seven compounds were isolated, including three of them that were previously unknown as natural products^45^. Although *S*. *albospinus* RLe7 showed a low cytotoxicity against cancer cell lines^44^, it induced a particular fungal response in the endophytic *Coniochaeta* sp. FLe4 due to the production of amphotericin B (**1**)^45^. These results suggest endophytes may have the potential to produce chemical compounds during microbial interactions.

With the purpose of investigating the chemical arsenal of endophytic microorganisms, randomly selected strains from our collection of endophytic strains of the Brazilian medicinal plant *L*. *ericoides* were submitted to pairwise microbial interactions. In this study, we present the microbial interactions between five actinobacteria (*S*. *cattleya* RLe1, *S*. *mobaraensis* RLe3, *S*. *albospinus* RLe7, *Streptomyces* sp. RLe9 and *Kytasatospora cystarginea* RLe10) and two fungi (*Colletotrichum boninense* FLe8.1 and *Coniochaeta* sp. FLe4). Particularly, chemical families from *S*. *mobaraensis* RLe3, which showed a high cytotoxicity activity against cancer cells in our previous study^44^, and *S*. *albospinus* RLe7, an amphotericin B (**1**) producer^45^, were identified by performing complementary approaches involving mono- and co-cultures, characterization of isolated compounds via NMR and MS, as well as molecular networking workflow.

Therefore, we report the identification of chemical families (pyrroloindole alkaloids, polyene macrocycles, leupeptins and angucyclines) produced by endophytic actinobacteria, characterization of cytotoxic agents (angucycline-derivatives) from *S*. *mobaraensis* RLe3, inducer activity of amphotericin B (**1**) (polyene macrocycle) from *S*. *albospinus* RLe7 by isolating a new fungal compound (**29**) from *Coniochaeta* sp. FLe4, characterization of a new compound from *S*. *mobaraensis* RLe3 (**23**) (angucycline-derivative), and showed the impact of inter-kingdom interactions between actinobacteria and fungi. Finally, we contribute with the annotation of the experimental MS/MS spectra from the 29 identified microbial natural products to the GNPS library (http://gnps.ucsd.edu)^27^.

## Results and Discussion

### Pairwise inter-kingdom interactions

**Figure 1:**
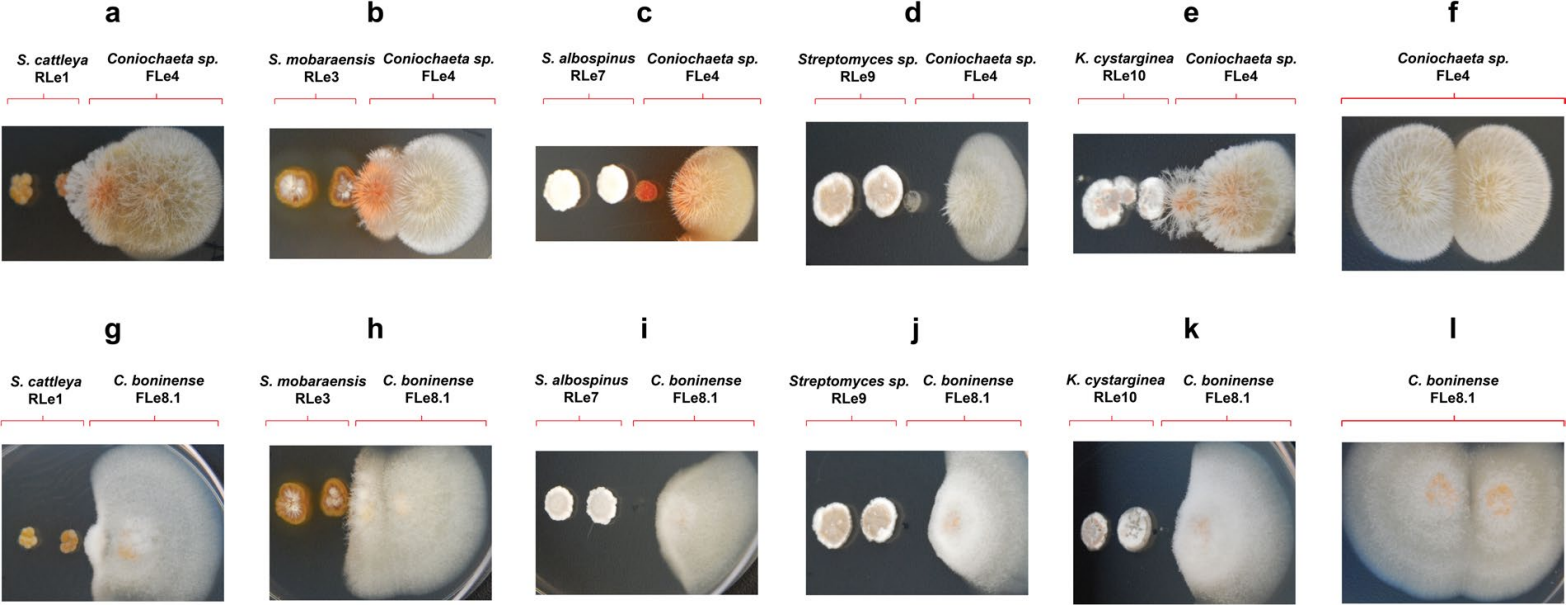
Pairwise inter-kingdom interactions involving endophytic fungi *Coniochaeta* sp. FLe4 and *C*. *boninense* FLe8.1 with endophytic actinobacteria from *L*. *ericoides*. Actinobacteria: *S*. *cattleya* RLe1, *S*. *mobaraensis* RLe3, *S*. *albospinus* RLe7, *Streptomyces* sp. RLe9, *K*. *cystarginea* RLe10. Fungi: *Coniochaeta* sp. FLe4 and *C*. *boninense* FLe8.1. Every interaction involved two colonies of each microorganism. Photos taken after 96 hours of cultivation.

Microbial interactions were performed between the endophytic strains of actinobacteria (*S*. *cattleya* RLe1, *S*. *mobaraensis* RLe3, *S*. *albospinus* RLe7, *Streptomyces* sp. RLe9 and *Kytasatospora cystarginea* RLe10) and fungi (*Colletotrichum boninense* FLe8.1 and *Coniochaeta* sp. FLe4) (Supplementary Fig. S1). It was observed that co-culturing the fungi *Colletotrichum boninense* FLe8.1 and *Coniochaeta* sp. FLe4 with actinobacteria had different impact on fungal development (Fig. 1). The actinobacteria affected fungal growth of the closest colonies of the fungus *Coniochaeta* sp. FLe4, but did not completely inhibit the fungus (Fig. 1a–e). A red pigmented phenotype was visible when *Coniochaeta* sp. FLe4 was co-cultured with four of the actinobacteria (Fig. 1a–c and e). This phenotype was most pronounced during the interaction with *S*. *albospinus* RLe7 (Fig. 1c). The colony of *C*. *boninense* FLe8.1 most proximal to actinobacteria did not grow in any of the interactions and no pigmentation was observed (Fig. 1g–k). These observations suggested the presence of antifungal compounds produced by actinobacteria.

### Identification of antibiotics/antifungals from actinobacteria by molecular networking

To reveal the small molecules produced during the pairwise inter-kingdom interactions, particularly the metabolite responsible for the induction of the red phenotype of *Coniochaeta* sp. FLe4 (Fig. 1c), molecular networking workflow was performed. Molecular network of microbial interactions enabled easy visualization of specific microbial metabolic features and identification of the producer microorganisms (Supplementary Fig. S2). Briefly, in a molecular network, every node represents one chemical entity, while clusters of nodes correspond to structurally related molecules based on similarity of their MS/MS spectra pattern^27^. While some nodes are annotated as previously characterized compounds, clusters including those metabolites are called molecular families^46^. A complementary strategy composed by the built-in automatic library search by GNPS^27^, manual confirmation based on MS/MS fragmentation pattern, accurate mass and NMR data of isolated compounds were used to verify and confirm the molecules and chemical classes described as follows.

**Figure 2:**
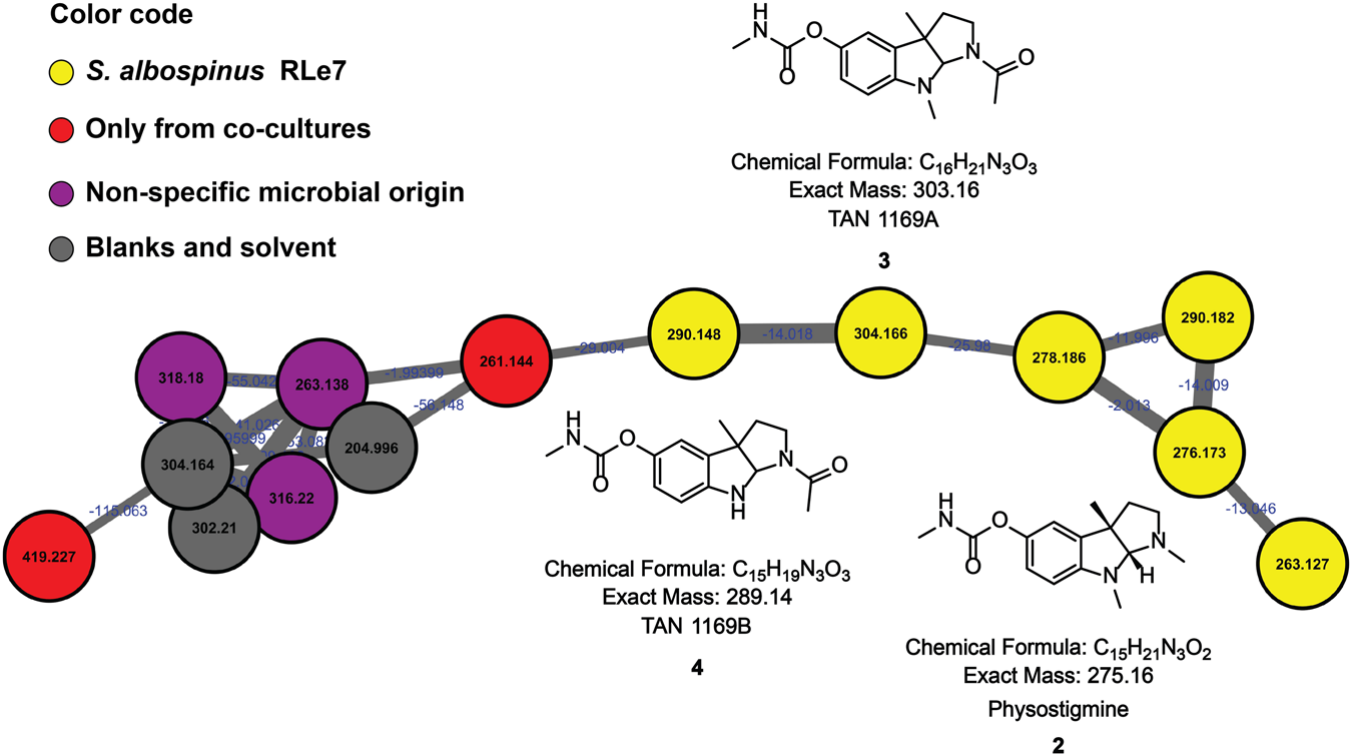
Pyrroloindole alkaloids-cluster from the molecular network of interactions among endophytic microorganisms from *L*. *ericoides*. Nodes from co-cultures are red, nodes from several strains (non-specified microbial origin) are purple, nodes observed in mono- and co-cultures involving *S*. *albospinus* RLe7 are in yellow.

One of the metabolites previously identified from *S*. *albospinus* RLe7 corresponds to the pyrroloindole alkaloid physostigmine (compound **2**)^44^. As it is a characterized natural product from this endophytic actinobacterium, the detection of physostigmine during microbial interaction was expected. In addition, the MS/MS fragmentation pathway of this well-known anticholinesterase compound has been reported^47^. The node corresponding to physostigmine clustered with other potentially related molecules (Figs 2 and S3–5). Two of them where consistent with the chemical formula of antibiotics TAN1169A (compound **3**) and B (compound **4**), previously reported natural products^48^, recently found to be intermediates of the physostigmine biosynthesis^49^. Although physostigmine was consistently detected from samples of mono- and co-cultures of *S*. *albospinus* RLe7, its biosynthesis was negatively affected by microbial interactions (Supplementary Fig. S6). Compounds **3** and **4** were also detected in mono- and co-culture samples (Supplementary Figs S7 and S8), and represented as yellow nodes (Fig. 2). Therefore, these three pyrroloindole alkaloid analogues were detected in mono- and co-culture samples. Physostigmine (**2**) has no reported antifungal activity, and it was not responsible for the induction of the red phenotype of *Coniochaeta* sp. FLe4 when interacting with *S*. *albospinus* RLe7^45^. However, the insecticidal activity of this compound against silkworm larvae and the importance of the *N*-8 methyl group in this bioactivity was suggested^50^. It is possible that physostigmine, as well as its analogues (compounds **3** and **4**), play an ecological role by protecting plants against insects, illustrating the importance of endophytes for symbiotic interactions.

**Figure 3:**
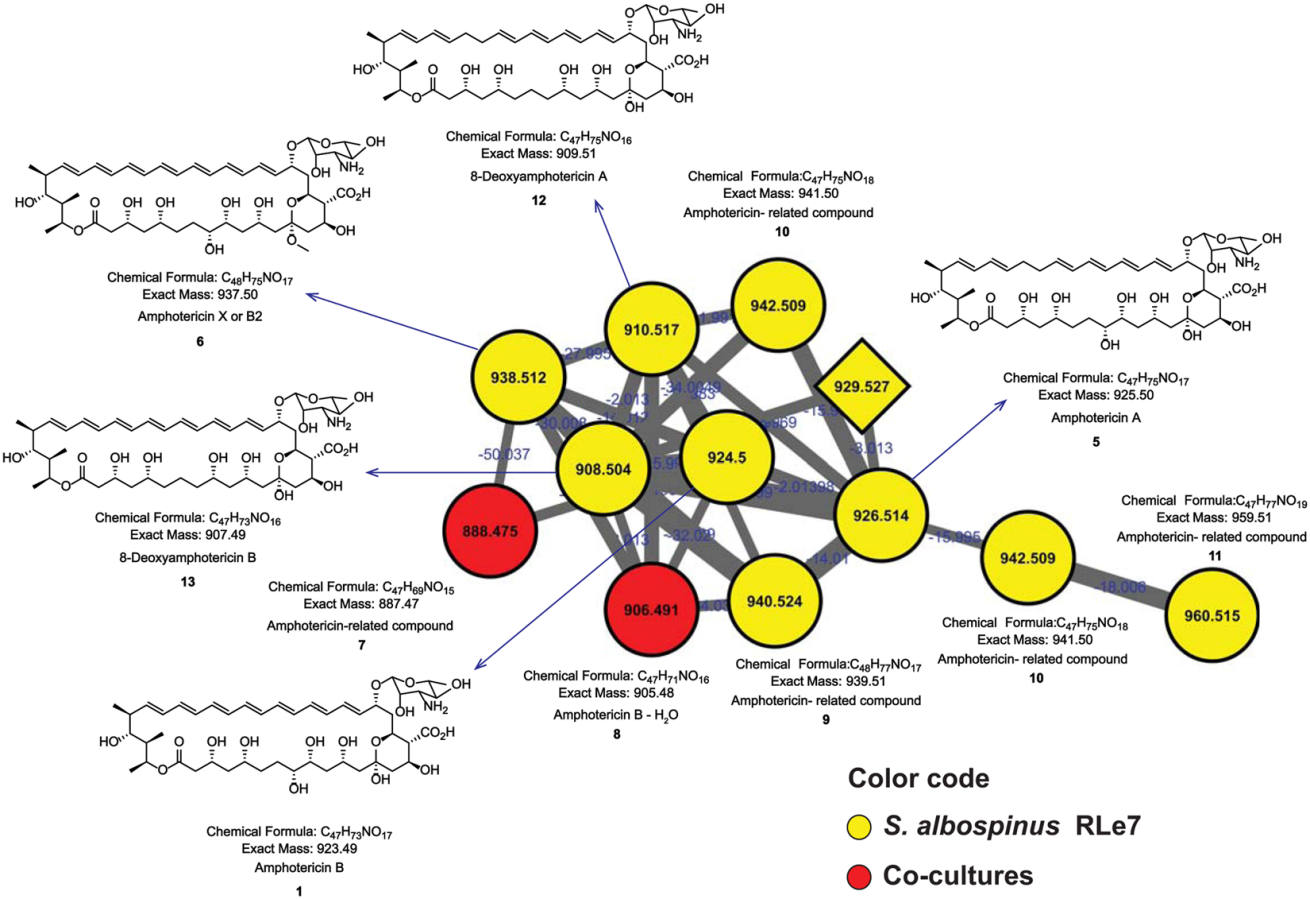
Amphotericin-cluster from the molecular network of interactions among endophytic microorganisms from *L*. *ericoides*. Nodes from co-cultures are red, nodes observed only in mono-cultures are differentiated by a diamond shape, nodes originated from *S*. *albospinus* RLe7 in mono- and co-cultures are yellow and round nodes.

The automatic library search by GNPS suggested the presence of amphotericin-derivatives by similarity of fragmentation patterns. However, manual verification of MS/MS and accurate mass were necessary to confirm the presence of amphotericin B (compound **1**) (Figs 3 and S9). MS/MS fragmentation of this class of compounds under our acquisition parameters was useful for identification of related compounds, due to their characteristic fragmentation patterns^51^. Therefore, it was confirmed that *S*. *albospinus* RLe7 also produced macrocyclic polyenes, related to amphotericin B (compound **1**). This cluster suggested the presence of several analogues, such as amphotericin A (compound **5**) (Supplementary Fig. S10), in consistency with the previous reports about coproduction of amphotericin A (**5**) and B (**1**)^52, 53^. Besides that, some “*impurities*”^54^, or even sub-products that may correspond to extracting adducts, such as amphotericin X or B_2_ (compound **6**) (Supplementary Fig. S11), which possess a methoxy group at C-13 position^55^, were identified. Additional putative analogues with mass differences were consistent with variations in the oxygenation and double bond patterns, such as the ions of *m/z* 888 (compound **7**), *m/z* 906 (compound **8**), *m/z* 940 (compound **9**), *m/z* 942 (compound **10**) and *m/z* 960 (compound **11**). This cluster was also interesting because of the presence of some analogues with molecular formulas of compounds obtained by genetically engineered strains, such as 8-deoxyamphotericin A (compound **12**) (Supplementary Fig. S12) and B (compound **13**) (Supplementary Fig. S13)^56^. Manual verification of the detected amphotericin-analogues enabled us to confirm that compounds **1**, **5**–**13** were detected in mono- and co-cultures (Supplementary Figs S14–S23). The detection of the ions of *m/z* 888 and *m/z* 906 at low levels from samples of mono-cultures from *S*. *albospinus* RLe7 affected the acquisition of MS/MS, decreasing the quality of the spectra due to contribution of noise signals, resulting in both nodes represented as features only observed from co-cultures (red nodes, Fig. 3). Although some amphotericin analogues have been investigated from a pharmaceutical point of view^57^, their role in the environment remain to be characterized. Since we demonstrated the involvement of this chemical class in the induction of the described particular fungal response of *Coniochaeta* sp. FLe4, visualized as a red-pigmented phenotype^45^, chemical signaling is perhaps a potential role polyene macrocycles may have in nature.

**Figure 4:**
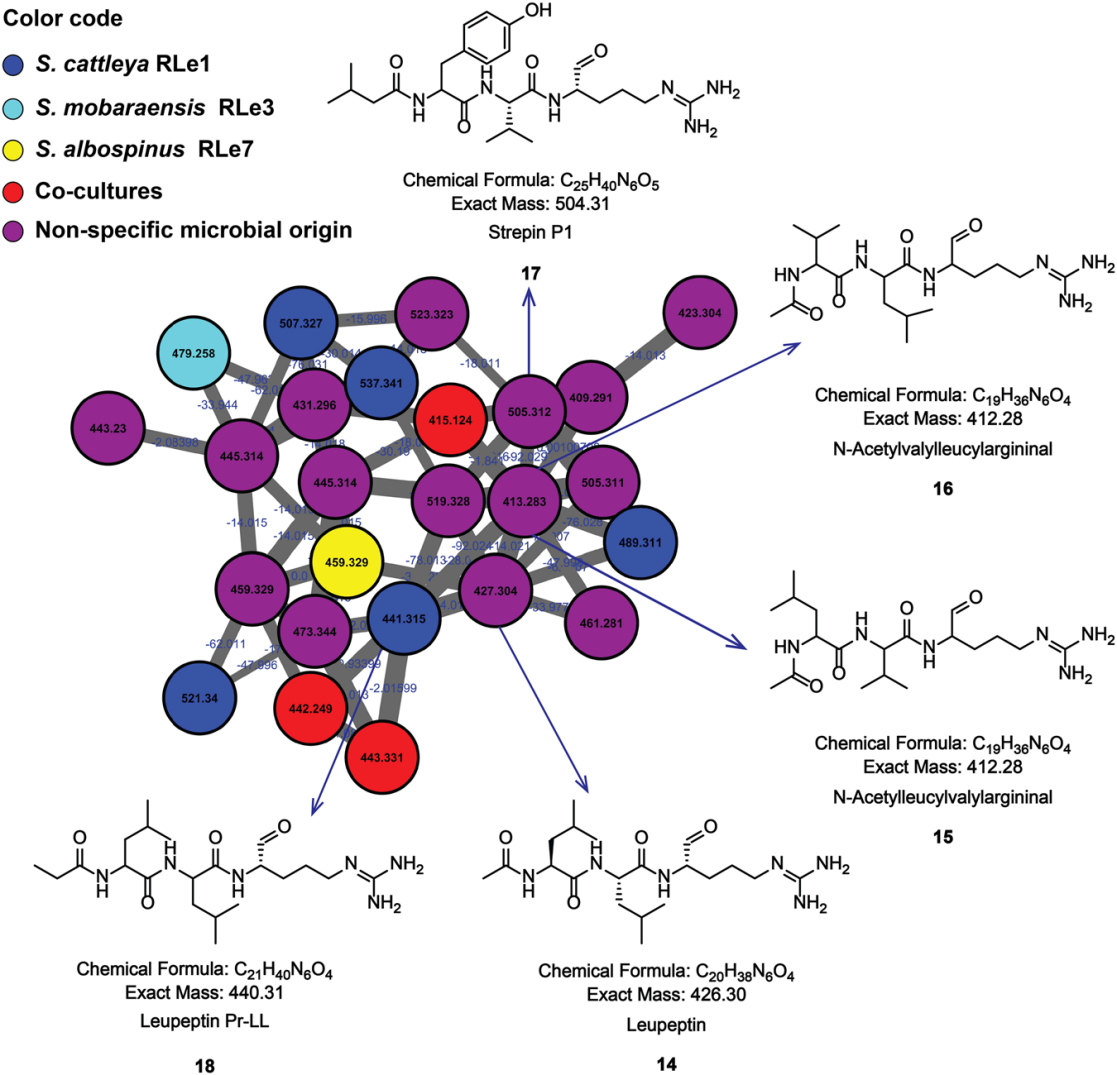
Leupeptin-cluster from the molecular network of interactions among endophytic microorganisms from *L*. *ericoides*. Nodes from co-cultures are red, nodes from several strains (unspecified microbial origin) are purple, nodes from mono- and co-cultures of *S*. *cattleya* RLe1 are in blue, nodes from mono- and co-cultures of *S*. *mobaraensis* RLe3 are in aquamarine, nodes from mono- and co-cultures of *S*. *albospinus* RLe7 are in yellow.

A widely distributed chemical class identified in this study corresponds to leupeptins (Fig. 4). The identification of leupeptin, *m/z* 427 (compound **14**), based on the automatic library search by GNPS and manual verification of its fragmentation pattern (Supplementary Fig. S23), enabled the putative annotation of the ions of *m/z* 413 and *m/z* 505 as *N*-acetylleucylvalylargininal (compound **15**) (or *N*-acetylvalylleucylargininal, compound **16**) (Supplementary Fig. S25) and strepin P1 (**17**) (Supplementary Fig. S26), respectively. Additionally, the ion of *m/z* 441 was correctly identified as Leupeptin Pr-LL (compound **18**) (Supplementary Fig. S27). Manual verification enabled us to confirm that leupeptins **14** and **15** (or **16**) were produced by *S*. *albospinus* RLe7 and *S*. *cattleya* RLe1 (Supplementary Figs S28–S31). Strepin P1 (**17**) was produced by *S*. *cattleya* RLe1 (Supplementary Fig. S32) but also produced by *K*. *cystarginea* RLe10 (Supplementary Fig. S33). However, compound **17** was detected in mono-cultures involving *K*. *cystarginea* RLe10 and co-cultures with *S*. *mobaraensis* RLe3 (Supplementary Fig. S33), suggesting the other interactions led to inhibit its production. Finally, compound **18** was produced by *S*. *cattleya* RLe1 and detected in mono- and co-cultures (Supplementary Fig. S34). Protein inhibitors, such as leupeptin (**14**) and analogues (**15–18**), have been reported from *Streptomyces* ^58–60^ and also from marine *Alteromonas* ^61^. Compound **15**, also proteinase inhibitor, previously isolated from *Streptomyces* and *Alteromonas* species, has been also obtained by synthesis^61, 62^, while the thrombin inhibitor **16**, has been reported from *S*. *flavogriseus* ^63, 64^. Strepin P1 (**17**) was previously isolated from *S*. *tanabaensis* by bioactivity-guided fractionation showing proteinase inhibition^65^. Then, leupeptins are widely produced by actinomycetes^59^, which is also consistent with the obtained cluster of leupeptins visualized in the molecular network (Fig. 4), where leupeptin were not restricted to just one strain. Although the specific role of leupeptins in actinobacteria is not known, the involvement of leupeptins in morphological differentiation of mycelia has been demonstrated, giving a first insight into the natural role of this family of compounds during colony development of actinobacteria in the environment^66, 67^.

### Angucycline-derivatives from *S*. *mobaraensis* RLe3

**Figure 5:**
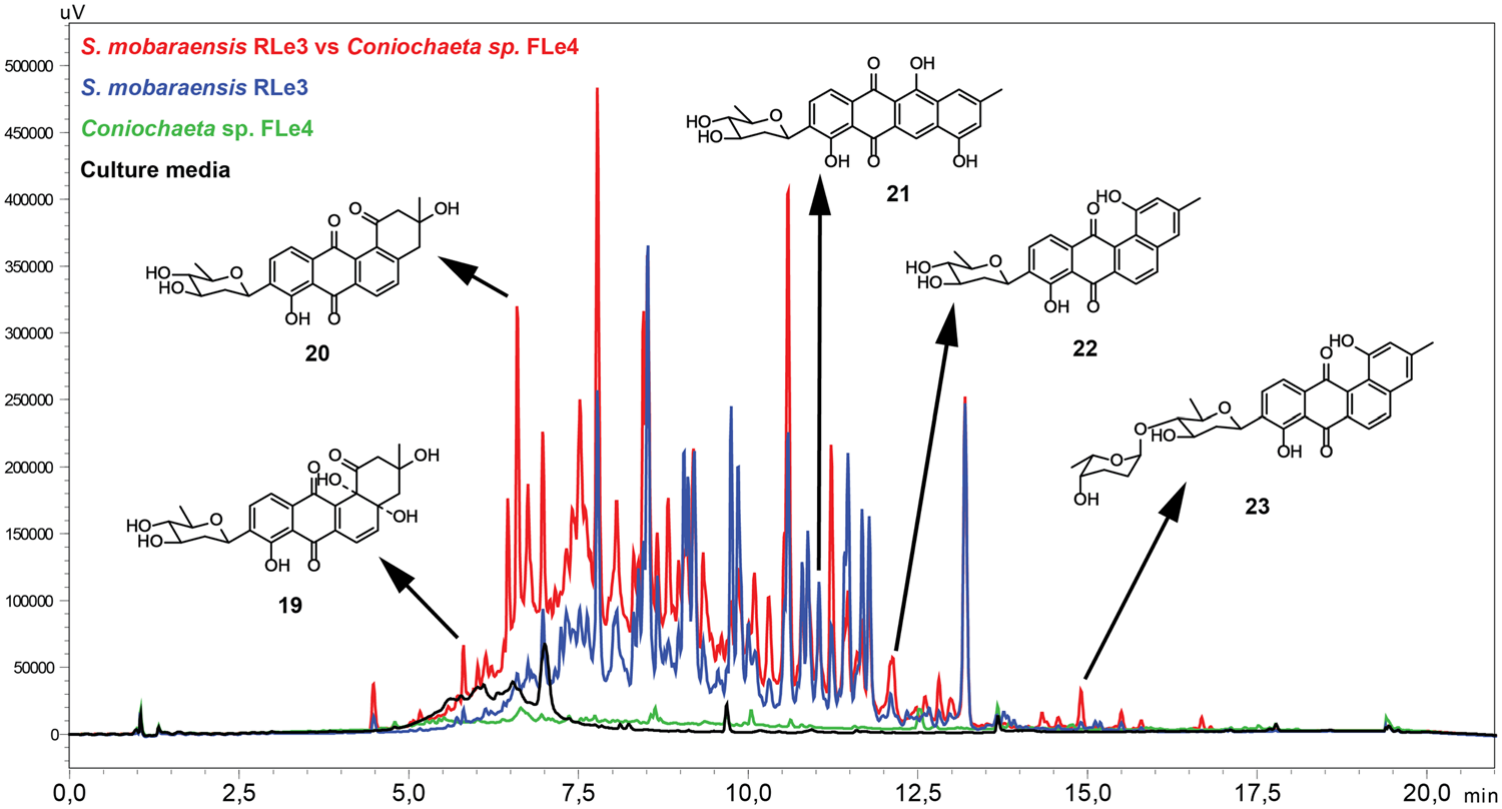
HPLC-DAD profile of extracts from microbial interaction between *S*. *mobaraensis* RLe3 and *Coniochaeta* sp. FLe4 in liquid culture. Overlaid chromatograms at 225 nm from culture media (black), *S*. *mobaraensis* RLe3 (blue), *Coniochaeta* sp. FLe4 (green), co-culture between RLe3 and FLe4 (red). Chemical structures from angucycline-derivatives isolated in this study. Urdamycinone B (**20**) is a representative metabolite overproduced by *S*. *mobaraensis* RLe3 during co-culture with *Coniochaeta* sp. FLe4 in liquid media.

Since *S*. *mobaraensis* RLe3 also induced a red pigmented phenotype during co-culture in *Coniochaeta* sp. FLe4, we hypothesized that *S*. *mobaraensis* RLe3 also produced a compound responsible for eliciting this phenotype. Additional cultures in liquid and parboiled rice media of *S*. *mobaraensis* RLe3 were performed in order to investigate the chemical profile of this strain. A preliminary screening of inter-kingdom interactions in liquid media showed microbial interactions between *S*. *mobaraensis* RLe3 and *Coniochaeta* sp. FLe4 as an interesting co-culture for induction of microbial metabolites. Therefore, large-scale co-culture was performed with this interaction (Fig. 5). Chemical investigation of co-culture between *S*. *mobaraensis* RLe3 and *Coniochaeta* sp. FLe4 led to the isolation of three known compounds (Supplementary Fig. S35): aquayamycin (**19**) (Supplementary Figs S36–S40 and Supplementary Table S1), urdamycinone B (**20**) (Supplementary Figs S41–S45 and Supplementary Table S2) and galtamycinone (**21**) (Supplementary Figs S46–S50 and Supplementary Table S3). Overproduction of metabolites was observed when *Coniochaeta* sp. FLe4 was present during microbial interaction in liquid culture (Fig. 5). This observation was more obvious for urdamycinone B (**20**), but was also evident for aquayamycin (compound **19**).

Additionally, large-scale mono-culture on parboiled rice led to the isolation of two compounds (Supplementary Fig. S51): dehydroxyaquayamycin (**22**) (Supplementary Figs S52–S56 and Supplementary Table S4) and marangucycline A_2_ (**23**) (Supplementary Figs S57–S62 and Supplementary Table S5). The compound **23** showed similar NMR data and chemical formula consistent with a recently isolated new metabolite, marangucycline A^68^. Interestingly, NMR data acquired from this compound (Supplementary Figs S57–S62 and Supplementary Table S5) suggested the presence of a distinct sugar subunit. While marangucycline A contains a β-D-olivose and α-L-amicetose moiety, our data indicated a different configuration of the hydroxyl group at the C-4″ position, consistent with the presence of α–L-rhodinose instead of α-L-amicetose^69^. We named this analogue as marangucycline A_2_ (**23**). Additionally, the levels of compounds **22** and **23** were increased during microbial interaction in liquid co-culture of *S*. *mobaraensis* RLe3 and *Coniochaeta* sp. FLe4, as shown in Fig. 5.

**Figure 6:**
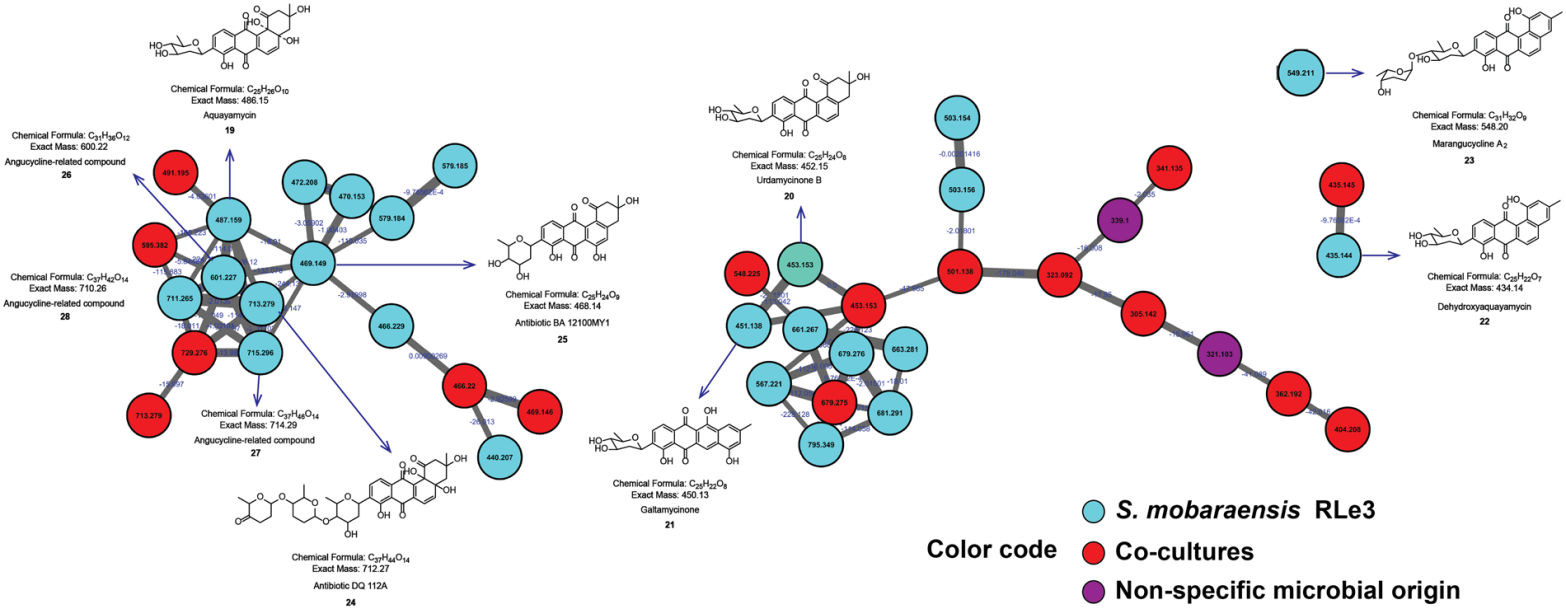
Angucyclines-clusters from molecular network of interactions among endophytic microorganisms from *L*. *ericoides*. Nodes from co-cultures are red and nodes from mono- and co-cultures of *S*. *mobaraensis* RLe3 are in aquamarine.

Therefore, *S*. *mobaraensis* RLe3 was confirmed as an angucycline-producer by NMR and HR-MS. Although the mass fragmentation of this class of compounds has not been studied in detail, clusters representing angucycline-derivatives were visualized in the molecular network from microbial interactions in solid media (Fig. 6). Aquayamycin (**19**) (Supplementary Fig. S63), previously isolated from actinobacteria^70–72^, was also detected from mono- and co-cultures involving *S*. *mobaraensis* RLe3 (Supplementary Fig. S64). Additional angucycline-related compounds were also detected. For instance, the antibiotics DQ112A (compound **24**)^73^ (Supplementary Figs S65 and S66) and BA12100MY1 (compound **25**)^74^, (Supplementary Figs S67 and S68) detected in mono- and co-cultures (*m/z* 713 and *m/z* 469, respectively), clustered with aquayamycin (**19**) highlighting structural similarities (Fig. 6). Additionally, the ions of *m/z* 601 (compound **26**), *m/z* 715 (compound **27**) and *m/z* 711 (compound **28**) were putatively annotated as angucycline analogues since their suggested chemical formula were consistent with at least two possible matches for each of them from the DNP database (Dictionary of Natural Products)^75^, and were detected in mono- and co-cultures involving *S*. *mobaraensis* RLe3 (Supplementary Figs S69-71). Urdamycinone B (**20**) was detected in mono- and co-cultures during interactions in solid media (Supplementary Figs S72 and S73). Urdamycinone B (**20**) was previously reported as a member of angucyclinones with an anthraquinone chromophore^76^. Galtamycinone (**21**)^77, 78^, which has been already reported from microbial sources^79^, was detected in mono- and in co-cultures and its fragmentation pattern showed similarity to urdamycinone B (**20**) (Supplementary Figs S74 and S75), resulting in both of them clustered together (Fig. 6). Dehydroxyaquayamycin (**22**), previously described as natural product from marine actinobacteria^80^, originally obtained as dehydrated product of aquayamycin (**19**)^72^, was detected in mono- and co-cultures involving *S*. *mobaraensis* RLe3 (Supplementary Figs S76 and S77). Dehydroxyaquayamycin (**22**) was found in low abundance which resulted in fragment spectra with additional peaks from chemical noise. Consequently, a separate two-nodes cluster was created by the algorithm (Fig. 6). The compound corresponding to marangucycline A_2_ (**23**) was detected in mono- and co-cultures of *S*. *mobaraensis* RLe3 (Supplementary Figs S78 and S79). Due to the detection of the corresponding ion of *m/z* 549 at low abundance, this compound (**23**) was represented as a single node in the molecular network (Fig. 6).

Angucyclines constitute the largest group of polycyclic aromatic polyketides with a wide range of biological activities^81^. Although this class of compounds has been widely reported, few studies have been carried out in order to reveal their function in nature. Recently, it was demonstrated the role of a “pseudo” gamma-butyrolactone receptor, which responded to exogenous angucyclines, in the regulation of the biosynthesis of endogenous antibiotics as well as its involvement in morphological development of *Streptomyces* ^82^. That study opened the door for understanding the ecological impact of natural products as mediators of microbial signaling. Although none of the angucycline-derivatives isolated in this study induced the red pigmentation in *Coniochaeta* sp. FLe4 (Supplementary Fig. S80), other compounds yet to be identified may be involved in this fungal response. On the other hand, the presence of angucycline-derivatives explained the high cytotoxic activities previously obtained from extracts of *S*. *mobaraensis* RLe3^44^.

### Structural elucidation of a new fungal metabolite

**Figure 7:**
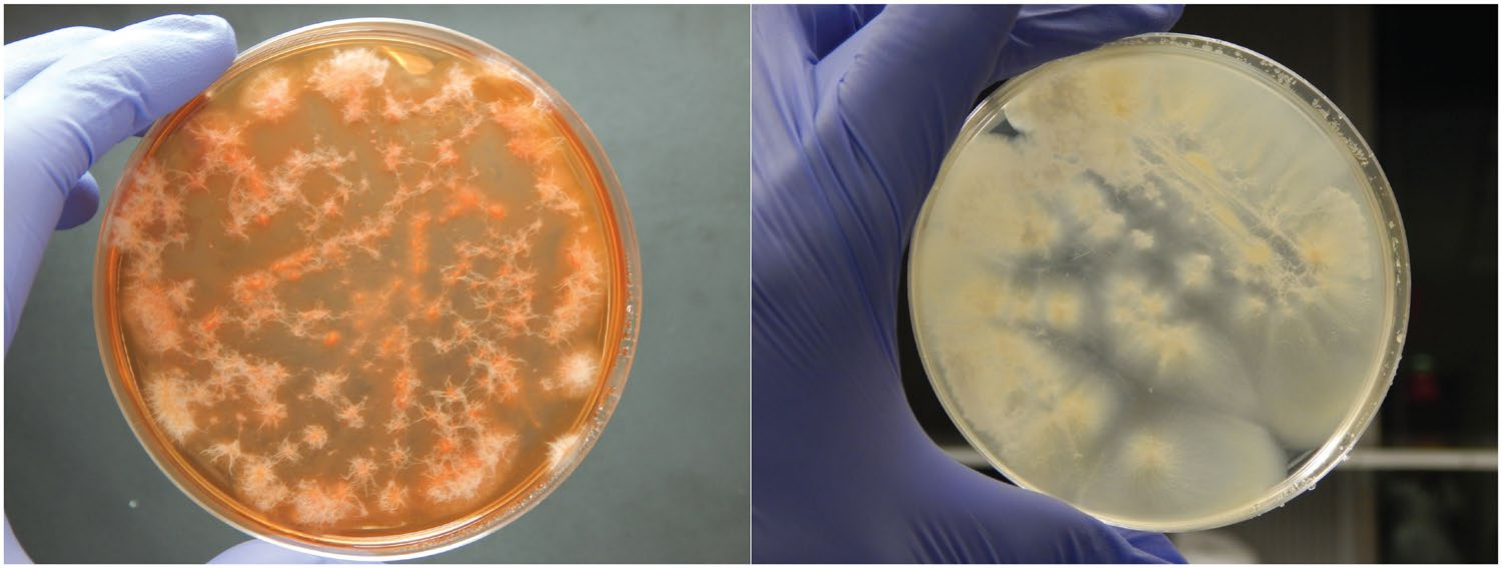
Comparison of cultures involving *Coniochaeta* sp. FLe4 in presence and absence of amphotericin B. *Coniochaeta* sp. FLe4 cultured in amphotericin B-enriched ISP-2 medium at 2 µM (left) and in absence of amphotericin B, cultured only in ISP-2 medium (right). Photos were taken after six days of culture. For experimental details, see description for isolation of compound **29** in **Materials and Methods** section.

**Figure 8:**
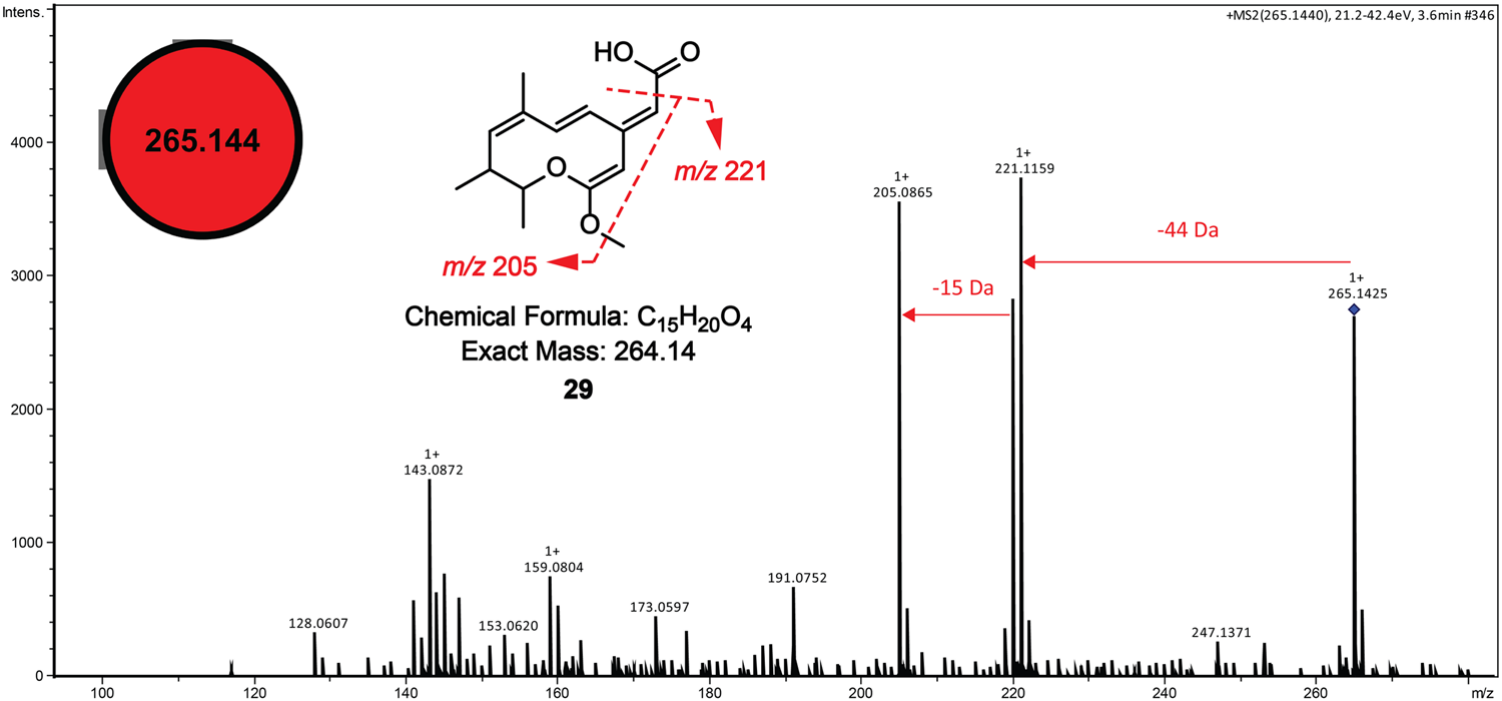
Node and representative MS/MS spectrum of *m/z* 265 (compound **29**) from *Coniochaeta* sp. FLe4. Red node corresponds to detected metabolites from microbial interactions (co-cultures). Specifically, the node of *m/z* 265 was detected from samples of co-cultures involving *Coniochaeta* sp. FLe4 with *S*. *cattleya* RLe1, *S*. *mobaraensis* RLe3, *S*. *albospinus* RLe7 and *K*. *cystarginea* RLe10.

Based on the observation that the strongest red pigmented phenotype of the fungus *Coniochaeta* sp. FLe4 was induced when interacting with *S*. *albospinus* RLe7 (Fig. 1c), the identification of amphotericin B (**1**) from this actinobacteria, and the previous finding that this antifungal compound (**1**) is at least one of the responsible agents for inducing the red pigmented phenotype^45^, large-scale cultivation of *Coniochaeta* sp. FLe4 in presence of amphotericin B (**1**) was performed (Fig. 7). After several efforts to isolate the apparently induced compounds from the red pigment mixture, this fungal culture led to the isolation of compound **29** (Supplementary Figs S81 and S82), corresponding to the ion of *m/z* 265 (Fig. 8). Structural elucidation based on 1D and 2D NMR experiments (^1^H, *g*COSY, *g*HSQC, *g*HMBC, NOESY, NOE DIFF, TOCSY) (Supplementary Figs S83–S90 and Supplementary Table S6) together with HR-MS-MS/MS led to a suggested molecular formula of C_15_H_20_O_4_, which was consistent with six degrees of unsaturation. Three methyl groups were detected as two duplets at δ_H_ 1.05 (*J* = 6.7 Hz) and 1.12 (*J* = 6.3 Hz) and one as a singlet at δ_H_ 1.87. HMBC correlation between hydrogens of a methoxy group at δ_H_ 3.86 to a carbon at δ_C_ 173.7 suggested the attachment of the methoxy group to a deshielded C sp^2^ which is linked to an oxygen atom. Two olefinic hydrogens at δ_H_ 6.19 (*J* = 15.7 Hz) and δ_H_ 7.11 (*J* = 15.7 Hz) showed *trans* correlation according to their *J* value. Experiment of NOE differential showed spatial correlation of the olefinic hydrogen at δ_H_ 5.57 to the methoxy group. Besides that, HMBC showed correlations of δ_H_ 5.57 to δ_C_ 101.4, δ_C_ 173.7 and δ_C_ 166.5. Additionally, HMBC correlations of δ_H_ 6.19 to δ_C_ 166.5 enabled to consistently suggest a free carboxyl group at δ_C_ 166.5 and the methoxy group to be linked to δ_C_ 173.7. Due to the low amount of sample it was not possible to directly detect the δ_C_ 160.7, which was attributed due to HMBC correlations to δ_H_ 6.14 and δ_H_ 6.19. The fragmentation pattern of compound **29** supports the possibility for a free carboxyl group that can lead to the product ion of *m/z* 220, while a subsequent loss of the methoxy group is consistent with the presence of the product ion of *m/z* 205. Together, our data support the proposal of the fungal compound **29** (Fig. 8).

In order to verify if induction of compound **29** was a consequence of microbial interaction, manual inspection of the raw LC-MS/MS data used to create the molecular network was performed. It was observed that the ion of *m/z* 265, corresponding to compound **29**, was detected from microbial interactions involving *Coniochaeta* sp. FLe4 with the actinobacteria *S*. *cattleya* RLe1, *S*. *mobaraensis* RLe3, *S*. *albospinus* RLe7 and *K*. *cystarginea* RLe10. However, it was not observed, at least at detectable levels, from the interaction with *Streptomyces* sp. RLe9 or any of the microbial mono-cultures (Supplementary Fig. S91). In addition, manual verification of the LC-MS/MS data from cultures of *Coniochaeta* sp. FLe4 in presence and absence of amphotericin B enabled to confirm the presence of compound **29** from cultures in amphotericin B-enriched medium (Supplementary Fig. S92).

Finally, our results showed that amphotericin B acts as an inducer of a fungal response leading to the production of the new fungal compound (**29**). As it was recently demonstrated, antibiotics such as trimethoprim, can induce the production of encrypted metabolites^83^. Our data showed that compound **29** was produced when *Coniochaeta* sp. FLe4 interacted with *S*. *cattleya* RLe1, *S*. *mobaraensis* RLe3, *S*. *albospinus* RLe7 and *K*. *cystarginea* RLe10 but not with *Streptomyces* sp. RLe9 (Supplementary Fig. S91). This suggests a correlation with the observed red pigmented phenotype (Fig. 1a–c and e), not observed during interaction with *Streptomyces* sp. FLe9 (Fig. 1d). Notably, the fragmentation pattern of this compound shared no significant similarity with other nodes of the molecular network resulting in a single node or self-loop (Figs 8 and S2). This is one of the useful means to prioritize leads which correspond to structural uniqueness, as it has been illustrated for a *Nocardiopsis* metabolite, ciromicin A^84^. Further work focused on revealing the biological role of the fungal compound (**29**) will be necessary. Additionally, several induced metabolites may be part of the induced red pigmented complex since compound **29** is not a red pigment. Further investigation of this interesting finding may lead to the identification of other microbial natural products induced by compound **1**.

Demonstrating the biological role for every molecule in nature is challenging. Therefore, the investigation of natural products in microbial communities is more rational since microorganisms are not alone in nature. Here we demonstrated the impact of microbial interactions among endophytes from *L*. *ericoides* that led to an increased production of some metabolites. We revealed the presence of an anticholinesterase compound (physostigmine-analogues), an antifungal compound (amphotericin-analogues), protease inhibitors (leupeptin-analogues) and cytotoxic (angucycline-analogues) chemical entities during investigation of microbial interactions (Summary Table 1). We demonstrated that metabolic exchange may induce chemical and phenotypical responses in inter-kingdom interactions among endophytic actinobacteria and fungi from *L*. *ericoides*. This study encourages us for further work to completely reveal both the chemistry and the biological role of small molecules from interacting endophytic microorganisms.

**Table 1:** Summary of identified compounds in this study.

Compound number in this study	Calculated *m/z* for [M + H]^+^	Observed *m/z* for [M + H]^+^	Error (ppm)	Molecular formula [M+H]^+^	Annotated name at the GNPS library	GNPS Library Membership	Microbial source in this study
1	924.4951	924.5007	−6.0	C_47_H_74_NO_17_	Amphotericin B	Annotation - this study	*S*. *albospinus* RLe7
2	276.1707	276.1732	−9.2	C_15_H_22_N_3_O_2_	Physostigmine	Massbank:KO002828	*S*. *albospinus* RLe7
3	304.1656	304.1660	−1.4	C_16_H_22_N_3_O_3_	Antibiotic TAN1169A	Annotation - this study	*S*. *albospinus* RLe7
4	290.1499	290.1504	−1.7	C_15_H_20_N_3_O_3_	Antibiotic TAN1169B	Annotation - this study	*S*. *albospinus* RLe7
5	926.5108	926.5162	−5.9	C_47_H_76_NO_17_	Amphotericin A	Annotation - this study	*S*. *albospinus* RLe7
6	938.5108	938.5142	−3.6	C_48_H_76_NO_17_	Amphotericin X or B2	Annotation - this study	*S*. *albospinus* RLe7
7	888.4740	888.4762	−2.5	C_47_H_70_NO_15_	Amphotericin-related compound	Annotation - this study	*S*. *albospinus* RLe7
8	906.4846	906.4877	−3.5	C_47_H_72_NO_16_	Dehydrated amphotericin B	Annotation - this study	*S*. *albospinus* RLe7
9	940.5264	940.5282	−1.9	C_48_H_78_NO_17_	Amphotericin-related compound	Annotation - this study	*S*. *albospinus* RLe7
10	942.5057	942.5077	−2.1	C_47_H_76_NO_18_	Amphotericin-related compound	Annotation - this study	*S*. *albospinus* RLe7
11	960.5163	960.51990	−3.8	C_47_H_78_NO_19_	Amphotericin-related compound	Annotation - this study	*S*. *albospinus* RLe7
12	910.5158	910.5151	0.8	C_47_H_76_NO_16_	Deoxyamphotericin A	Annotation - this study	*S*. *albospinus* RLe7
13	908.5002	908.5056	−5.9	C_47_H_74_NO_16_	Deoxyamphotericin B	Annotation - this study	*S*. *albospinus* RLe7
14	427.3027	427.3052	−5.8	C_20_H_39_N_6_O_4_	Leupeptin	Massbank:KO009038	*S*. *cattleya* RLe1 and *S*. *albospinus* RLe7
15–16	413.2871	413.2878	−1.7	C_19_H_37_N_6_O_4_	Leupeptin analogue LVR or VLR	Annotation - this study	*S*. *cattleya* RLe1 and *S*. *albospinus* RLe7
17	505.3133	505.3102	6.1	C_25_H_41_N_6_O_5_	Strepin P1	Annotation - this study	*S*. *cattleya* RLe1 and *K*. *cystarginea* RLe10
18	441.3184	441.3197	−3.0	C_21_H_41_N_6_O_4_	Leupeptin Pr-LL	Annotation - this study	*S*. *cattleya* RLe1
19	487.1599	487.1590	1.8	C_25_H_27_O_10_	Aquayamycin	Annotation - this study	*S*. *mobaraensis* RLe3
20	453.1544	453.1548	−0.9	C_25_H_25_O_8_	Urdamycinone B	Annotation - this study	*S*. *mobaraensis* RLe3
21	451.1387	451.1382	1.4	C_25_H_23_O_8_	Galtamycinone	Annotation - this study	*S*. *mobaraensis* RLe3
22	435.1438	435.1457	−4.3	C_25_H_23_O_7_	Dehydroxyaquayamycin	Annotation - this study	*S*. *mobaraensis* RLe3
23	549.2119	549.2115	0.7	C_31_H_33_O_9_	Marangucycline A_2_	Annotation - this study	*S*. *mobaraensis* RLe3
24	713.2804	713.2843	−5.5	C_37_H_45_O_14_	Angucycline-related compound	Annotation - this study	*S*. *mobaraensis* RLe3
25	469.1493	469.1493	0.0	C_25_H_25_O_9_	Angucycline-related compound	Annotation - this study	*S*. *mobaraensis* RLe3
26	601.2280	601.2269	1.8	C_31_H_37_O_12_	Angucycline-related compound	Annotation - this study	*S*. *mobaraensis* RLe3
27	715.2960	715.2962	−0.2	C_37_H_47_O_14_	Angucycline-related compound	Annotation - this study	*S*. *mobaraensis* RLe3
28	711.2647	711.2658	−1.5	C_37_H_43_O_14_	Angucycline-related compound	Annotation - this study	*S*. *mobaraensis* RLe3
29	265.1434	265.1440	−2.1	C_15_H_21_O_4_	(*E*)-2-((2*Z*,5*E*,7*Z*)-2-methoxy-7,9,10-trimethyl-9,10-dihydro-4*H*-oxecin-4-ylidene) acetic acid.	Annotation - this study	*Coniochaeta* sp. FLe4

## Materials and Methods

### Strains and culture conditions

#### Endophytic strains

Endophytic microorganisms belong to the collection of the Laboratory of Chemistry of Microorganisms of the School of Pharmaceutical Sciences of Ribeirão Preto, University of São Paulo. The selected actinobacteria strains included in this work (*Streptomyces cattleya* RLe1, *S*. *mobaraensis* RLe3, *S*. *albospinus* RLe7, *Streptomyces* sp. RLe9 and *Kytasatospora cystarginea* RLe10) were isolated from the roots of *L*. *ericoides* and identified as previously described^44^. The fungus *Coniochaeta* sp. FLe4 was isolated from the leaves of the same plant and identified as described^44, 45^. The fungal strain *Colletotrichum boninense* FLe8.1 was isolated from the leaves of the same plant and its identification will be published soon. Permission of accessing and research with endophytic microorganisms from *L*. *ericoides* was provided by the Brazilian government under process number CNPq 010858/2014-8.

#### Reactivation of endophytic strains

Endophytic microorganisms were reactivated from their sterile mineral oil stocks^44^ in SFM agar medium (2 g soy flour (Sigma-Aldrich), 2 g agar (Sigma-Aldrich), 2 g mannitol (Sigma-Aldrich) per 100 mL of deionized water) for 7–10 days at 30 °C. Small pieces of SFM media-containing mycelia (5 mm diameter) were transferred to 4 mL of ISP-2 liquid (4 g yeast extract (Sigma-Aldrich), 10 g malt extract (Sigma-Aldrich), 4 g dextrose (Sigma-Aldrich) per 1 L of deionized water) and incubated for 72 hours in rotatory shaker at 200 rpm and 27–30 °C, obtaining a pre-culture.

#### Microbial interactions -inter-kingdom interactions- in solid media

In order to perform the microbial interactions, 1 μL of the pre-culture was spotted on Petri dishes (10 mm) containing 10 mL of ISP-2 agar media (15 g agar per 1 L of ISP-2). Mono-cultures of each microorganisms were performed by adding two 1 μL-spots of the pre-culture at 5 mm distance on the culture media. Co-cultures were performed following the same procedure, adding two 1 μL-spots of one microorganism pre-culture at 5 mm distance, and two 1 μL-spots of the other microorganism pre-culture at 5 mm distance (Supplementary Fig. S1 and Fig. 1). Four replicated samples from each mono- and co-culture were prepared. Microbial cultures were incubated at 30 °C during 96 h and then extraction was performed to prepare samples for subsequent LC- MS/MS analysis.

#### Large-scale of *Streptomyces mobaraensis* RLe3 in liquid co-culture with *Coniochaeta* sp. FLe4

Co-culture of *S*. *mobaraensis* RLe3 against *Coniochaeta* sp. FLe4 was performed as follows: 4 mL of the pre-culture in ISP-2 liquid containing *S*. *mobaraensis* RLe3, as described in the ***Strains and culture conditions*** section, and 12 plugs (5 mm diameter) of the reactivated *Coniochaeta* sp. FLe4 on SFM media containing fungal mycelia, as described in the ***Strains and culture conditions*** section, were transferred to 200 mL of ISP-2 in 500 mL-Erlenmeyer flasks. A total of 18 Erlenmeyer flasks were incubated on rotatory shaker at 30 °C and 200 rpm during 11 days.

#### Large-scale of *Streptomyces mobaraensis* RLe3 in mono-culture on parboiled rice

Mono-culture of *Streptomyces mobaraensis* RLe3 in parboiled rice was performed as described previously^44^, with some modifications briefly explained as following: 4 mL of the pre-culture in ISP-2 containing *S*. *mobaraensis* RLe3, as described in the ***Strains and culture conditions*** section, were transferred to 40 g of sterile parboiled rice contained in 500 mL-Erlenmeyer flasks. A total of 20 Erlenmeyer flasks were incubated at 30 °C during 40 days.

#### Large-scale of *Coniochaeta* sp. FLe4 in presence of amphotericin B

Amphotericin B (A2411 Sigma-Aldrich) was firstly dissolved in DMSO/MeOH 1:1 and added into sterilized ISP-2 agar at a final concentration of 2 µM. One hundred Petri dishes containing 10 mL of ISP-2 agar at a final concentration of 2 µM amphotericin B were prepared and inoculated with 500 µL of *Coniochaeta* sp. FLe4 from a 72 h pre-culture in ISP-2 liquid, as described in the ***Strains and culture conditions*** section. Plates were incubated under 30 °C for six days.

### Instrumentation for purification via HPLC and NMR acquisition

HPLC purification of described compounds were carried out on a Shimadzu Prominence Nexera XR HPLC coupled to LC-6AD pumps, SPD-M20A Prominence Diode Array Detector, CTO-20A Prominence Column Oven, CBM-20A Communication Bus Module, and Class VP software. 1D and 2D NMR spectra were acquired on Bruker (R) – DRX500-Ultra Shield (R) (^1^H: 500.13 MHz, ^13^C: 125.77 MHz) and Bruker Avance III HD 600-MHz spectrometers.

### Extraction, fractionation and purification procedures

#### Large-scale of *Streptomyces mobaraensis* RLe3 in liquid co-culture with *Coniochaeta* sp. FLe4

Extraction of the large-scale culture of the microbial interaction between *S*. *mobaraensis* RLe3 and *Coniochaeta* sp. FLe4 in liquid culture was carried as follows: after 11 days of co-culture, microbial cells were removed by filtration and the supernatant was extracted using SPE C-18. Briefly, SPE C-18 (10 g) cartridges (Phenomenex®) were conditioned with water, the filtrate was added to the cartridge and washed with water. The extract was eluted with MeOH and dried under vacuum. Sephadex LH20 (2.5 cm × 64 cm) was used for separation of ~975 mg of extract followed by further SPE C-18 fractionation before HPLC purification yielded the corresponding fractions containing the angucycline-related compounds **19**, **20** and **21**, respectively (Purification workflow and purified HPLC-DAD peaks: Supplementary Figs S35, S36, S41 and S46).

#### Large-scale of *Streptomyces mobaraensis* RLe3 in mono-culture on parboiled rice

Extraction of the large-scale culture for the scale up culture of *S*. *mobaraensis* RLe3 on parboiled rice was performed as follows: MeOH was added directly into the Erlenmeyer flasks containing the cultured microorganism, sonicated for 15 min, the methanolic extract was filtered and the elutant dried on a rotatory evaporator. Then, 1 g of the methanolic extract was subjected to fractionation by using Amberlite® XAD-16N resin (20–60 mesh, 4.5 cm × 32 cm column) with sequential elution using MeOH/water 1:1 until 100% MeOH was reached, followed by elution with 100% acetone. The acetone extract (202 mg) was subjected to further chromatographic steps by using Sephadex® LH20 (2.5 cm × 64 cm column). Additional purification steps included either SPE C-18 or Si (10 g cartridge), as well as HPLC yielded the corresponding fractions containing the angucycline-related compounds **22** and **23**, respectively (Purification workflow purified HPLC-DAD peaks: Supplementary Figs S51, S52 and S57).

#### Purification of angucycline-derivatives

Further purification of the angucycline-derivatives was performed on a Phenomenex® Gemini C6-Phenyl 110 A semipreparative (5 µm × 250 mm × 10 mm) column. Solvent B: ACN, solvent A: water. Solvents were HPLC grade. The gradient employed for chromatographic separation was: 0–2 min 5% solvent B, 2–32 min linear gradient from 5% B to 100% B, 32–36 min 100% B, 36–40 min 100% to 5% B, 40–45 min 5% B at a flow rate of 4.7 mL/min throughout the run. Peaks of interest eluted at: 14.5 min (compound **19**, 700 µg), 16.5 min (compound **20**, 6.8 mg), 23.8 min (compound **21**, 200 µg), 25.4 min (compound **22**, 400 µg), 28.9 min (compound **23**, 280 µg).

#### Large-scale of *Coniochaeta* sp. FLe4 in presence of amphotericin B

The large-scale cultures of *Coniochaeta* sp. FLe4 on ISP-2 media, enriched with amphotericin B (A2411 Sigma-Aldrich) at a final concentration of 2 µM, were extracted with 1 L of MeOH after six days of culture, sonicated for 15 min, filtered and dried by rotatory evaporation. The extract (1 g) was dissolved in 10 mL of milli-Q water and fractionated by using solid Phase Extraction (SPE) C-18 cartridge (10 g, Phenomenex®). The SPE cartridge was previously washed with MeOH and equilibrated with water. Gradient fractionation was carried out with water and increasing proportions of MeOH by 10%, collecting volumes of 100 mL until MeOH 100%. The eluted fraction of 60% MeOH contained the target compound (**29**). Further purification was carried out on a Phenomenex® Gemini C18 110 A semipreparative (5 µm × 250 mm × 10 mm) column under isocratic condition at 35% B. at a flow rate of 4.7 mL/min. Solvent B: ACN, solvent A: water, both solvents were HPLC grade. Peak of interest eluted at 15.9 min yielding approximately 300 µg of compound **29** (Purification workflow and purified HPLC-DAD peak, Supplementary Figs S81 and S82).

Confirmation of all the purified peaks was performed by using analytical column Ascentis® Express C18. Solvent B: ACN, solvent A: water, both solvents were HPLC grade. Conditions: 0–1 min 5% B, 1–16 min 5–100% B, 16–18 min 100% B, 18–20 min 100–5%, total run 21 min. Flow rate of 1 mL/min.

### NMR data of purified compounds

Compound **19**: NMR (Supplementary Table S1 and Supplementary Figs S37–S40) and MS (HR-ESI-MS *m/z* 487.1590 (calcd for C_25_H_27_O_10_, [M + H]^+^, 487.1599) data are consistent with the structure of aquayamycin^72^.

Compound **20**: NMR (Supplementary Table S2 and Supplementary Figs S42–S45) and MS (HR-ESI-MS *m/z* 453.1548 (calcd for C_25_H_25_O_8_, [M + H]^+^, 453.1544) data are consistent with the structure of urdamycinone B^76^.

Compound **21**: NMR (Supplementary Table S3 and Supplementary Figs S47–S50) and MS (HR-ESI-MS *m/z* 451.1381 (calcd for C_25_H_23_O_8_, [M + H]^+^, 451.1387) data are consistent with the structure of galtamycinone^79^.

Compound **22**: NMR (Supplementary Table S4 and Supplementary Figs S53–S56) and MS (HR-ESI-MS *m/z* 435.1457 (calcd for C_25_H_23_O_7_, [M + H]^+^, 435.1438) data are consistent with the structure of dehydroxyaquayamycin^80^.

Compound **23** (*m/z* 549 [M + H]^+^) **(**see Supplementary Table S5 and Supplementary Figs S58–S62 for complete NMR data**)**: ^1^H NMR (500 MHz, CDCl_3_) δ 12.65 (s, 1H, 8-OH), 11.42 (s, 1H, 1-OH), 8.33 (d, *J* = 8.5 Hz, 1H, 6-H), 8.15 (d, *J* = 8.5 Hz, 1H, 5-H), 7.92 (d, *J* = 8.0 Hz, 1H, 10-H), 7.90 (d, *J* = 8.0 Hz, 1H, 11-H), 7.27 (s, 1H, 4-H), 7.16 (s, 1H, 2-H), 4.94 (d, *J* = 10.0 Hz, 1H, 1′-H), 4.25 (q, *J* = 6.5 Hz, 1H, 5″-H), 3.84 (m, 1H, 3′-H), 3.69 (s, 1H, 4″-H), 3.57 (m, 1H, 5′-H), 3.09 (t, *J* = 8.6 Hz, 1H, 4′-H), 2.56 (dd, *J* = 13.0, 4.6 Hz, 1H, 2′-Ha), 2.10 (m, 1H, 2″-Hb), 2.04 (m, 1H, 3″-Hb), 1.82 (m, 1H, 3″-Ha), 1.66 (m, 1H, 2″-Ha), 1.46 (m, 1H, 2′-Hb), 1.39 (d, *J* = 6.0 Hz, 3 H, 6′-H_3_), 1.27 (d, *J* = 6.6 Hz, 3H, 6″-H_3_). HR-ESI-MS *m/z* 549.2115 (calcd for C_31_H_33_O_9_, [M + H]^+^, 549.2119). Our NMR data, combined with MS data support the annotation of marangucycline A_2_.

Compound **29 (**See Supplementary Table S6 and Figs S83–S90 for complete NMR data**)**: ^1^H NMR (500 MHz, MeOH-*d*4) δ 7.11 (d, *J* = 15.7 Hz, 1H), 6.19 (d, *J* = 15.7 Hz, 1H), 6.14 (s, 1H), 5.73 (d, *J* = 10.3 Hz, 1H), 5.57 (s, 1H), 3.87 (s, 1H), 3.58 (br quint, 6.6, 6.3 Hz), 2.57 (dquint, *J* = 10.3, 6.7 Hz, 1H), 1.87 (s, 1H), 1.29 (s, 1H), 1.13 (d, *J* = 6.3 Hz, 1H), 1.05 (d, *J* = 6.7 Hz, 1H). ^13^C assignments through *g*HSQC and *g*HMBC (^1^H 500 MHz, ^13^C 125 MHz, MeOH-*d*4) δ 173.7, 166.5, 160.7, 141.4, 134.0, 117.6, 101.4, 143.5, 88.5, 56.6, 72.1, 41.9, 12.1, 21.2, 16.5. HR-ESI-MS *m/z* 265.1440 (calcd for C_15_H_21_O_4_, [M + H]^+^, 265.1434). The proposed structure corresponds to a new fungal compound, (*E*)-2-((2*Z*,5*E*,7*Z*)-2-methoxy-7,9,10-trimethyl-9,10-dihydro-4*H*-oxecin-4-ylidene) acetic acid.

### Sample preparation for LC-MS/MS

After the incubation time of microbial interactions -inter-kingdom interactions- on solid media (96 h), the region of interest was excised from the Petri dish for extraction procedures. The removed region containing the microbial colonies of interest (mono- or co-cultures) was transferred to Eppendorf tubes and 1 mL of extraction solvent was added. Four solvent mixtures were used, each one for each of the four replicates of the mono- and co-culture samples: (1:1 acetonitrile:methanol (ACN:MeOH); 1:1 ACN:water; 1:1 ACN:MeOH 0.1% formic acid, 1:1 ACN:water 0.1% formic acid). After 10 minutes of sonication, the supernatant was transferred to a clean vial, centrifuged at 16873 rcf (14000 rpm – radius of rotor ~76.865 mm Eppendorf Centrifuge 5418) for 15 minutes and 300 µL of the top extract were transferred to clean 0.5 mL 96-well polypropylene plates (Agilent Technologies Inc., Santa Clara, CA, USA) and sealed with Zone-Free Sealing Film (Excel Scientific) for LC-MS/MS analysis.

### LC-MS/MS experiments

Crude extracts were analyzed by UPLC-HRMS-MS/MS on an Agilent 1290 UPLC using a Kinetex^™^ 50 mm × 2.1 mm C18 RP column (1.7 µm particle size) coupled to a MicrOTOF-QII mass spectrometer (Bruker Daltonics) equipped with the standard Apollo ESI source. Solvent B: ACN containing 0.1% formic acid, solvent A: water 0.1% formic acid, both solvents were of LC-MS grade. The gradient employed for chromatographic separation was 5% solvent B for 1 minute, linear gradient from 5% B to 95% B in 8 minutes, kept at 95% B for 2 minutes, back to 5% B in 1 minute and kept at 5% B for 1 minute to end up with total run time of 13 minutes at a flow rate of 0.5 mL/min. MS spectra were acquired in positive ion mode in the range of 50–2000 *m/z*. External calibration was performed prior to data collection using ESI-L Low Concentration Tuning Mix (Agilent Technologies). Hexakis (2,2-difluoroethoxy) phosphazene (Synquest Laboratories) *m/z* 622.028960, used for internal calibration, was added into a calibrant reservoir and placed inside the ion source. Other instruments settings were as follows: capillary voltage 4000 V, nebulizer gas pressure (N_2_) 2.0 bar, ion source temperature 200 °C, dry gas flow 9 L/min source temperature, spectral rate 3 Hz for MS1 and 10 Hz for MS2. For acquiring MS/MS fragmentation, 10 most intense ions per MS1 were selected for subsequent CID with stepped CID energy applied. More detailed parameters for tandem MS were used as previously published^85^.

### Molecular networking

Input data for molecular networking was generated through conversion of the LC-MS/MS raw data to.mzXML data format by using the Bruker Daltonics Software. Data were submitted to the molecular networking workflow at the GNPS platform (gnps.ucsd.edu)^27^. The public dataset of this work is available at ftp://massive.ucsd.edu/MSV000079048. Molecular networking output was imported and visualized by using Cytoscape^86^, version 8.3. The complete analysis can be accessed via: http://gnps.ucsd.edu/ProteoSAFe/status.jsp?task=5dd7b0a5aeca4354945bf5bd1bddac27.

The GNPS molecular network was created using the parameters as follows: the data was filtered by removing all MS/MS peaks within +/− 17 Da of the precursor *m/z*, then clustered with MS-Cluster with a parent mass tolerance of 0.5 Da and a MS/MS fragment ion tolerance of 0.5 Da to create consensus spectra. Further, only consensus spectra that contained 2 nearly identical spectra were considered. A network was then created where edges were filtered to have a cosine score above 0.7 and 6 or more matched peaks. Further edges between two nodes were kept in the network if and only if each of the nodes appeared in each other’s respective top 10 most similar nodes. The spectra in the network were then searched against GNPS’s spectral libraries. The library spectra were filtered in the same manner as the input data. All matches kept between network spectra and library spectra were required to have a score above 0.7 and at least 6 matched peaks. A total of 15676 spectra were considered corresponding to 1590 nodes, and 175 clusters containing 931 clusternodes in the molecular network.

### Minimal Inhibitory Concentration (MIC) assay against *Coniochaeta sp.* FLe4

MIC assay involving compound **23** and amphotericin B (**1**) was carried out according to a serial dilution^87^. The tested substance was prepared by serial dilutions to half of the concentration of the previous well, from a range of concentrations between 400 and 0.1953 µg/mL.

### Diffusion assay with purified compounds against *Coniochaeta sp*. FLe4

A chemical complementation assay with purified compounds was carried out. Briefly, 1 μL of a pre-culture of *Coniochaeta* sp. FLe4 in ISP-2 liquid media, as described in the ***Strains and culture conditions*** section, was spotted on Petri dishes (10 mm) containing 10 mL of ISP-2 agar media. A second spot of the solubilized compound at the required concentration was pipetted onto the plate. Plates were incubated at 30 °C for six days.

## Electronic supplementary material

Supplementary Information
