# Supplementary material for: Molecular inter-kingdom interactions of endophytes isolated from Lychnophora ericoides
Source: Sci Rep. 2017 Jul 14;7:5373. doi: 10.1038/s41598-017-05532-5 (PMC5511137; doi:10.1038/s41598-017-05532-5)
Supplement: Supplementary file 1 — Supplementary Information [file 41598_2017_5532_MOESM1_ESM.pdf]

Supplementary information

Molecular inter-kingdom interactions of endophytes isolated from *Lychnophora ericoides*

Authors: Andrés M Caraballo-Rodríguez[a], Pieter C Dorrestein[b], Monica T Pupo[a*]

Affiliation: [a]Faculdade de Ciências Farmacêuticas de Ribeirão Preto, Universidade de São Paulo, Ribeirão Preto, SP, Brazil, 14040-903; [b]Collaborative Mass Spectrometry Innovation Center, Skaggs School of Pharmacy and Pharmaceutical Sciences, University of California - San Diego, La Jolla, CA 92093.

Index of figures

Figure S1. Pairwise inter-kingdom interactions of endophytic microorganisms from *L. ericoides*. 5

Figure S2. Molecular network of microbial interactions amongst endophytic microorganisms from *L. ericoides*. 6

Figure S3. MS/MS spectrum of detected physostigmine (**2**) from the molecular network of interactions amongst endophytic microorganisms from *L. ericoides*. 7

Figure S4. MS/MS spectrum of detected TAN 1169A (**3**) from the molecular network of interactions amongst endophytic microorganisms from *L. ericoides*. 7

Figure S5. MS/MS spectrum of detected TAN 1169B (**4**) from the molecular network of interactions amongst endophytic microorganisms from *L. ericoides*. 8

Figure S6. Extracted ion chromatogram comparison for physostigmine (**2**) 8

Figure S7. Extracted ion chromatogram comparison for TAN 1169A (**3**) 9

Figure S8. Extracted ion chromatogram comparison for TAN 1169B (**4**) 9

Figure S9. MS/MS spectrum of detected amphotericin B (**1**) from the molecular network of interactions amongst endophytic microorganisms from *L. ericoides*. 10

Figure S10. MS/MS spectrum of detected amphotericin A (**5**) from the molecular network of interactions amongst endophytic microorganisms from L. ericoides. 11

Figure S11. MS/MS spectrum of putative amphotericin X or B2 (**6**) from molecular networking of interactions amongst endophytic microorganisms from *L. ericoides*. 12

Figure S12. MS/MS spectrum of putative deoxyamphotericin A (**12**) from the molecular network of interactions amongst endophytic microorganisms from *L. ericoides*. 13

Figure S13. MS/MS spectrum of putative deoxyamphotericin B (**19**) from the molecular network of interactions amongst endophytic microorganisms from *L. ericoides*. 14

Figure S14. Extracted ion chromatogram comparison for compound (**1**) 14

Figure S15. Extracted ion chromatogram comparison for compound (**5**) 15

Figure S16. Extracted ion chromatogram comparison for compound (**6**) 15

Figure S17. Extracted ion chromatogram comparison for compound (**7**) 16

Figure S18. Extracted ion chromatogram comparison for compound (**8**) 16

Figure S19. Extracted ion chromatogram comparison for compound (**9**) 17

Figure S20. Extracted ion chromatogram comparison for compound (**10**) 17

Figure S21. Extracted ion chromatogram comparison for compound (**11**) 18

Figure S22. Extracted ion chromatogram comparison for compound (**12**) 18

Figure S23. Extracted ion chromatogram comparison for compound (**13**) 19

Figure S24. MS/MS spectrum of detected leupeptin (**14**) from the molecular network of interactions amongst endophytic microorganisms from *L. ericoides*. 19

Figure S25. MS/MS spectrum of detected leupeptin acetyl-(LVR (**15**) or VLR (**16**)) from the molecular network of interactions amongst endophytic microorganisms from *L. ericoides*. 20

Figure S26. MS/MS spectrum of detected strepin P1 (**17**) from molecular network of interactions amongst endophytic microorganisms from *L. ericoides*. 21

Figure S27. MS/MS spectrum of detected leupeptin Pr-LL (**18**) from molecular network of interactions amongst endophytic microorganisms from *L. ericoides.* 21

Figure S28. Extracted ion chromatogram comparison for compound (**14**) from mono- and co-cultures involving *S. cattleya* RLe1 22

Figure S29. Extracted ion chromatogram comparison for compound (**14**) from mono- and co-cultures involving *S. albospinus* RLe7 22

Figure S30. Extracted ion chromatogram comparison for compound (**15**/**16**) from mono- and co-cultures involving *S. cattleya* RLe1 23

Figure S31. Extracted ion chromatogram comparison for compound (**15**/**16**) from mono- and co-cultures involving *S. albospinus* RLe7 23

Figure S32. Extracted ion chromatogram comparison for compound (**17**) from mono- and co-cultures involving *S. cattleya* RLe1 24

Figure S33. Extracted ion chromatogram comparison for compound (**17**) from mono- and co-cultures involving *K. cystarginea* RLe10 24

Figure S34. Extracted ion chromatogram comparison for compound (**18**) 25

Figure S35. Purification workflow for compounds **19**, **20** and **21** 26

Figure S36. HPLC-DAD of purified peak corresponding to compound (**19**) 26

Figure S37. ^{1}H NMR (500 MHz, MeOH-d_4) spectrum of aquayamycin (**19**). 27

Figure S38. gCOSY (500 MHz, MeOH-d_4) spectrum of aquayamycin (**19**). 27

Figure S39. gHSQC (500 MHz, MeOH-d_4) spectrum of aquayamycin (**19**). 28

Figure S40. gHMBC (500 MHz, MeOH-d_4) spectrum of aquayamycin (**19**). 28

Figure S41. HPLC-DAD of purified peak corresponding to compound (**20**) 29

Figure S42. ^{1}H NMR (500 MHz, acetone-d_6) spectrum of urdamycinone B (**20**). 29

Figure S43. gCOSY (500 MHz, acetone-d_6) spectrum of urdamycinone B (**20**). 30

Figure S44. gHSQC (500 MHz, acetone-d_6) spectrum of urdamycinone B (**20**). 31

Figure S45. gHMBC (500 MHz, acetone-d_6) spectrum of urdamycinone B (**20**). 32

Figure S46. HPLC-DAD of purified peak corresponding to compound (**21**) 32

Figure S47. ^{1}H NMR (500 MHz, MeOH-d_4) spectrum of galtamycinone (**21**). 33

Figure S48. gCOSY (500 MHz, MeOH-d_4) spectrum of galtamycinone (**21**). 34

Figure S49. gHSQC (500 MHz, MeOH-d_4) spectrum of galtamycinone (**21**). 35

Figure S50. gHMBC (500 MHz, MeOH-d4) spectrum of galtamycinone (**21**). 36

Figure S51. Purification workflow for compounds **22** and **23** 37

Figure S52. HPLC-DAD of purified peak corresponding to compound (**22**) 38

Figure S53. ^{1}H NMR (500 MHz, $CDCl_3$) spectrum of dehydroxyaquayamycin (**22**). 38

Figure S54. gCOSY (500 MHz, $CDCl_3$) spectrum of dehydroxyaquayamycin (**22**). 39

Figure S55. gHSQC (500 MHz, $CDCl_3$) spectrum of dehydroxyaquayamycin (**22**). 40

Figure S56. gHMBC (500 MHz, $CDCl_3$) spectrum of dehydroxyaquayamycin (**22**). 41

Figure S57. HPLC-DAD of purified peak corresponding to compound (**23**) 41

Figure S58. ^{1}H NMR (500 MHz, $CDCl_3$) spectrum of marangucycline A_2 (**23**). 42

Figure S59. COSY (300 MHz, $CDCl_3$) spectrum of marangucycline A_2 (**23**). 42

Figure S60. gHSQC (400 MHz, $CDCl_3$) spectrum of marangucycline A_2 (**23**). 43

Figure S61. gHMBC (500 MHz, $CDCl_3$) spectrum of marangucycline A_2 (**23**). 43

Figure S62. NOESY (500 MHz, $CDCl_3$) spectrum of marangucycline A_2 (**23**). 44

Figure S63. MS/MS spectrum of detected aquayamycin (**19**) from molecular networking of interactions amongst endophytic microorganisms from *L. ericoides*. 45

Figure S64. Extracted ion chromatogram comparison for compound (**19**) 45

Figure S65. MS/MS spectrum of detected aquayamycin analogue *m/z* 713 (**24**) from the molecular network of interactions amongst endophytic microorganisms from *L. ericoides*. 46

Figure S66. Extracted ion chromatogram comparison for compound (**24**) 46

Figure S67. MS/MS spectrum of detected aquayamycin analogue *m/z* 469 (**25**) from the molecular network of interactions amongst endophytic microorganisms from *L. ericoides*. 47

Figure S68. Extracted ion chromatogram comparison for compound (**25**) 47

Figure S69. Extracted ion chromatogram comparison for compound (**26**) 48

Figure S70. Extracted ion chromatogram comparison for compound (**27**) 48

Figure S71. Extracted ion chromatogram comparison for compound (**28**) 49

Figure S72. MS/MS spectrum of detected urdamycinone B (**20**) from the molecular network of interactions amongst endophytic microorganisms from *L. ericoides*. 50

Figure S73. Extracted ion chromatogram comparison for compound (**20**) 50

Figure S74. MS/MS spectrum of detected galtamycinone (**21**) from the molecular network of interactions amongst endophytic microorganisms from *L. ericoides*. 51

Figure S75. Extracted ion chromatogram comparison for compound (**21**) 51

Figure S76. MS/MS spectrum of detected dehydroxyaquayamycin (**22**) from the molecular network of interactions amongst endophytic microorganisms from *L. ericoides*. 52

Figure S77. Extracted ion chromatogram comparison for compound (**22**) 53

Figure S78. MS/MS spectrum of detected marangucycline A_2 (**23**) from the molecular network of interactions amongst endophytic microorganisms from *L. ericoides*. 54

Figure S79. Extracted ion chromatogram comparison for compound (**23**) 54

Figure S80. Biological assay of purified compounds against *Coniochaeta* sp. FLe4. 55

Figure S81. Purification workflow for compound (**29**) 56

Figure S82. HPLC-DAD of purified peak corresponding to compound (**29**) 56

Figure S83. ^{1}H NMR (500 MHz, MeOH-*d*4) spectrum of compound (**29**) 57

Figure S84. COSY (500 MHz, MeOH-*d*4) spectrum of compound (**29**) 58

Figure S85. gHSQC (500 MHz, MeOH-*d*4) spectrum of compound (**29**) 58

Figure S86. gHMBC (500 MHz, MeOH-*d*4) spectrum of compound (**29**) 59

Figure S87. NOE DIFF (500 MHz, MeOH-*d*4) spectrum of compound (**29**) 60

Figure S88. NOESY (500 MHz, MeOH-*d*4) spectrum of compound (**29**) 61

Figure S89. TOCSY 1D 1.12 ppm (600 MHz, MeOH-*d*4) spectrum of compound (**29**) 62

Figure S90. TOCSY 1D 6.19 ppm (600 MHz, MeOH-*d*4) spectrum of compound (**29**) 62

Figure S91. Comparison of extracted ion chromatograms (MS1) of *m/z* 265.1434±0.002 of mono- and co-cultures involving *Coniochaeta* sp. FLe4. 63

Figure S92. Comparison of extracted ion chromatograms (MS1) of *m/z* 265.1434±0.002 from samples of cultures involving *Coniochaeta* sp. FLe4 in presence and absence of amphotericin B. 64

Index of tables

Table S1. NMR Spectroscopy Data (500 MHz, MeOH-d_4) for compound **19** consistent with reported compound from literature.[1] 65

Table S2. NMR Spectroscopy Data (500 MHz, Acetone-d_6) for compound **20** consistent with reported data from literature.[2] 66

Table S3. NMR Spectroscopy Data (500 MHz, MeOH-d_4) for compound **21** consistent with reported data from literature.[3] 67

Table S4. NMR Spectroscopy Data (500 MHz, $CDCl_3$) for compound **22**, consistent with reported data from literature.[4] 68

Table S5. NMR Spectroscopy Data (500 MHz, $CDCl_3$) for marangucycline A[5] and compound **23** 69

Table S6. NMR Spectroscopy Data (500 MHz, MeOH-*d*4) for compound **29**, this study) 70

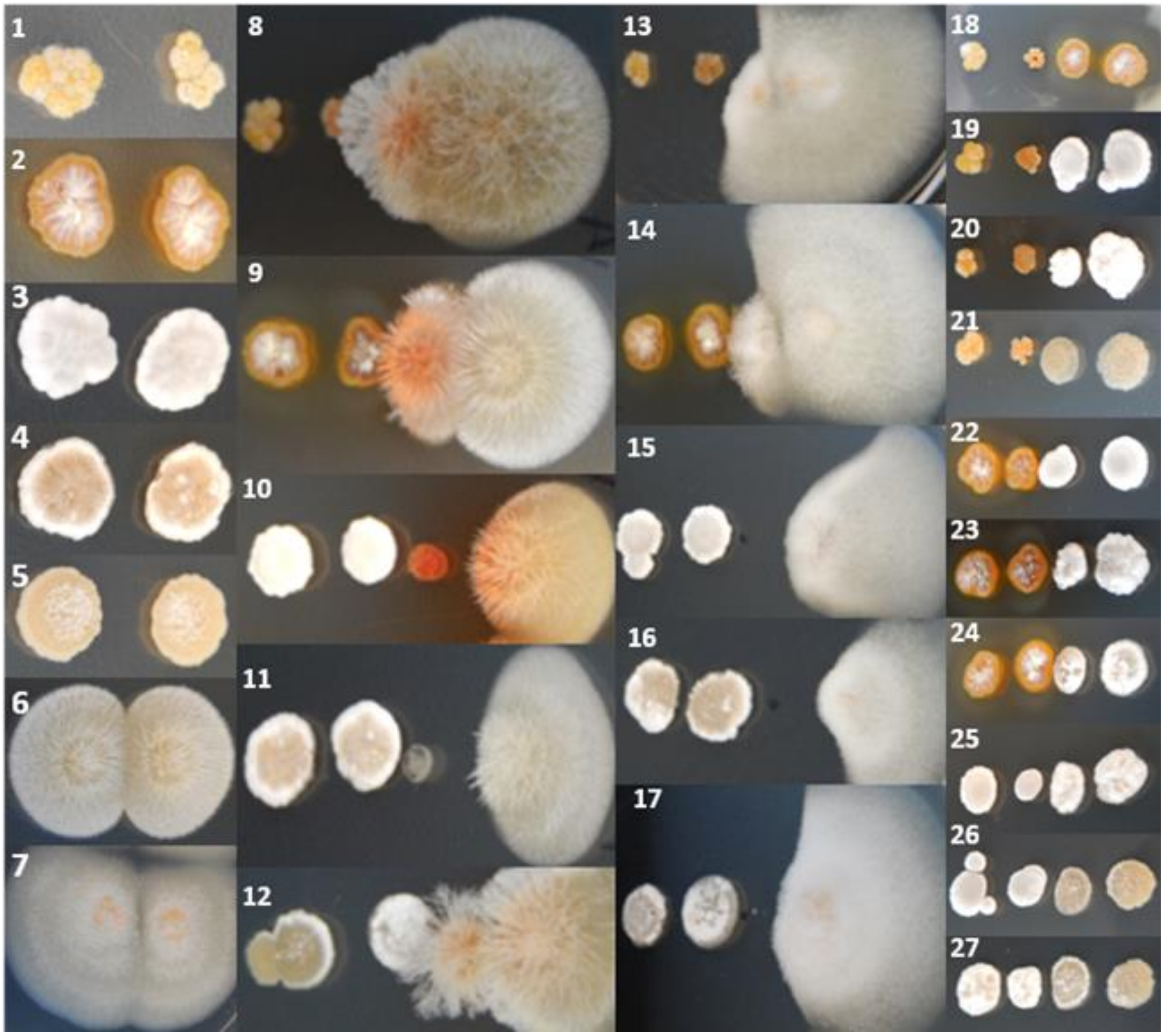

Figure S1. Pairwise inter-kingdom interactions of endophytic microorganisms from *L. ericoides*.

Actinobacteria: *S. cattleya* RLe1, *S. mobaraensis* RLe3, *S. albospinus* RLe7, *Streptomyces* sp. RLe9, *K. cystarginea* RLe10 and fungi: *Coniochaeta* sp. FLe4 and *Colletotrichum boninense* FLe8.1. Every interaction involved two colonies of each microorganism showing different phenotype when comparing interacting and not interacting colonies. **1.** RLe1; **2.** RLe3; **3.** RLe7; **4.** RLe9; **5.** RLe10; **6.** FLe4; **7.** FLe8.1; **8.** Left: RLe1, Right: FLe4; **9.** Left: RLe3, Right: FLe4; **10.** Left: RLe7, Right: FLe4; **11.** Left: RLe9, Right: FLe4; **12.** Left: RLe10, Right: FLe4; **13.** Left: RLe1, Right: FLe8.1; **14.** Left: RLe3, Right: FLe8.1; **15.** Left: RLe7, Right: Fle8.1; **16.** Left: RLe9, Right: FLe8.1; **17.** Left: RLe10, Right: FLe8.1; **18.** Left: RLe1, Right: RLe3; **19.** Left: RLe1, Right: RLe7; **20.** Left: RLe1, Right: RLe9; **21.** Left: RLe1, Right: RLe10; **22.** Left: RLe3, Right: RLe7, **23.** Left: RLe3, Right: RLe9; **24.** Left: RLe3, Right: RLe10; **25.** Left: RLe7, Right: RLe9; **26.** Left: RLe7, Right: RLe10; **27.** Left: RLe9, Right: RLe10.
Photos taken after 96 hours of cultivation.

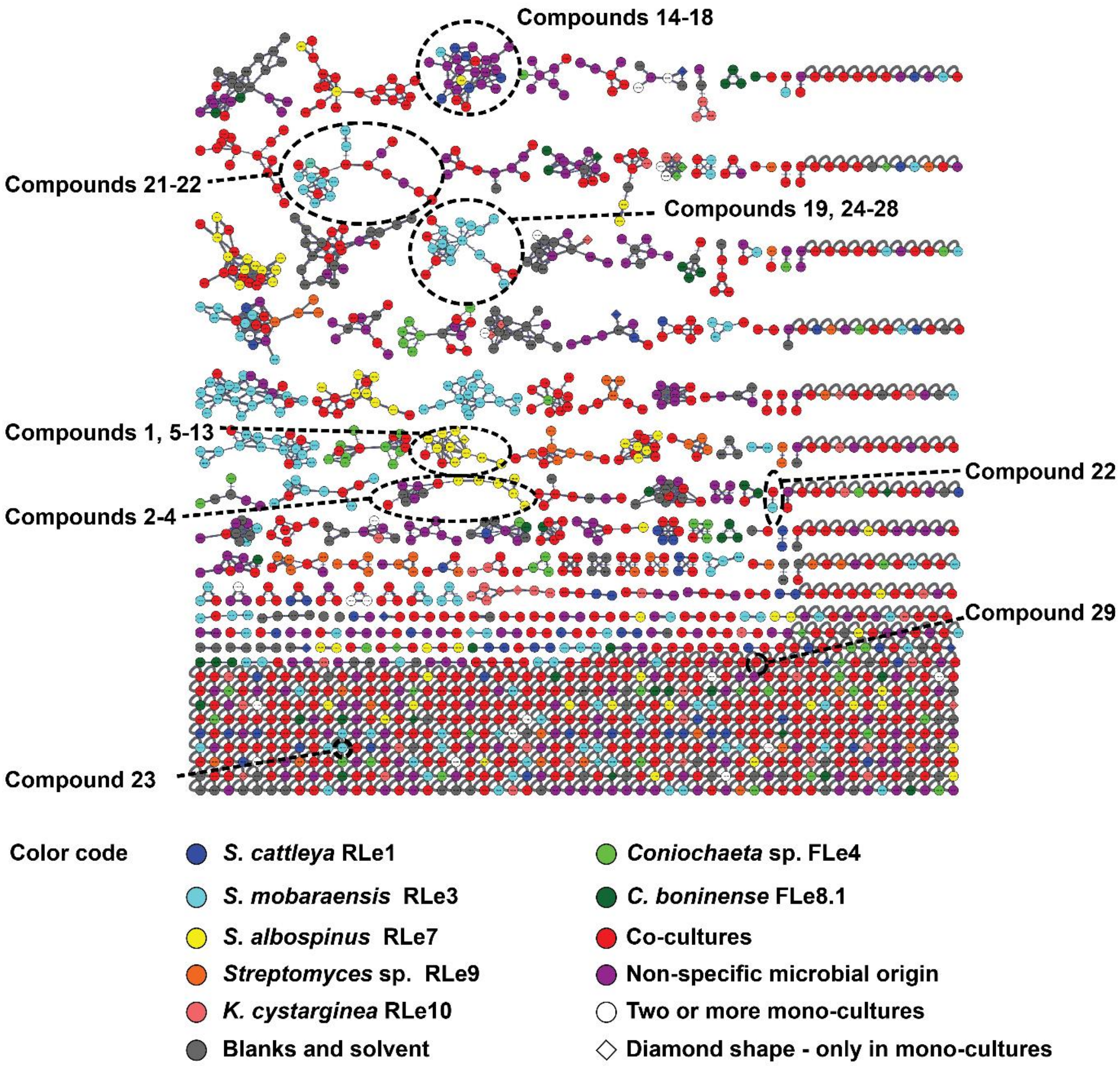

Figure S2. Molecular network of microbial interactions amongst endophytic microorganisms from *L. ericoides*.

Nodes represent molecules produced by each strain in mono- and co-cultures as shown in the color code description: *S. cattleya* RLe1 (blue), *S. mobaraensis* RLe3 (aquamarine), *S. albospinus* RLe7 (yellow), *Streptomyces* sp. RLe9 (orange), *K. cystarginea* RLe10 (pink) and fungi: *Coniochaeta* sp. FLe4 (light green) and *Colletotrichum boninense* FLe8.1 (dark green). Red nodes represent molecules detected in co-culture samples. Molecules detected from several strains (non-specific microbial origin) are represented by purple nodes. Molecules detected only from mono-cultures of each strain are differentiated by a diamond shape. Molecules detected from two or more mono-cultures of different strains are represented by white nodes. Grey nodes represent molecules from culture media and solvents.

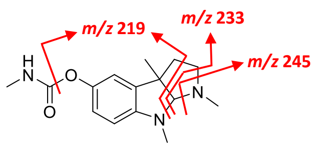

Physostigmine (**2**)
$C_{15}H_{21}N_3O_2$
$[M+H]^+$ Calcd.: 276.1707
$[M+H]^+$ Obs.: 276.1732
Δ = -9.2 ppm

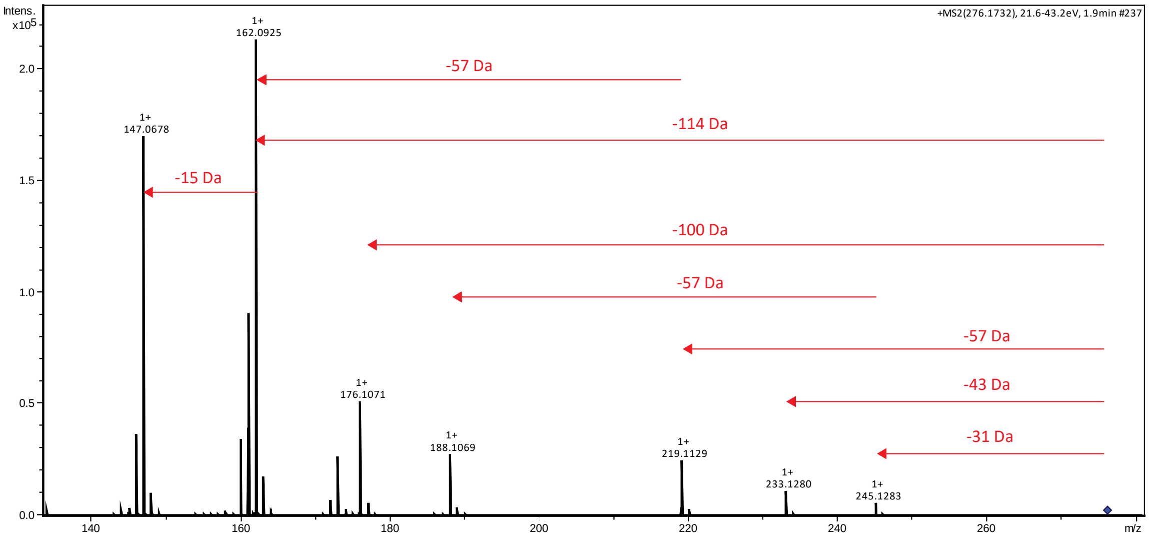

Figure S3. MS/MS spectrum of detected physostigmine (**2**) from the molecular network of interactions amongst endophytic microorganisms from *L. ericoides*.

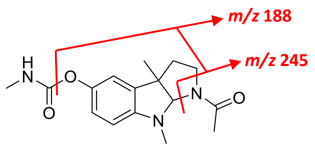

TAN 1169A **(3)**
$C_{16}H_{21}N_3O_3$
$[M+H]^+$ Calcd.: 304.1656
$[M+H]^+$ Obs.: 304.1660
Δ = -1.4 ppm

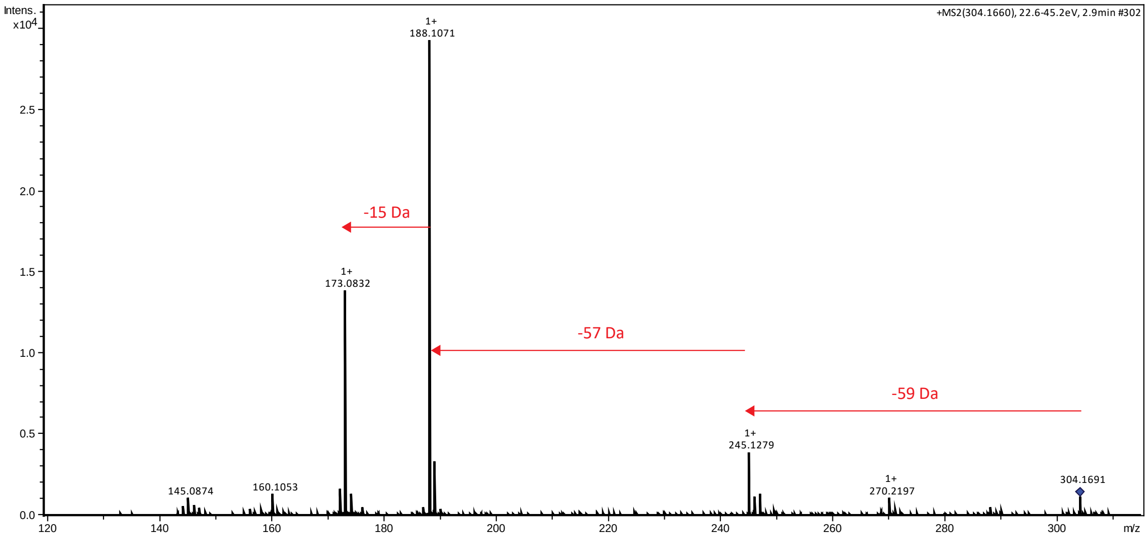

Figure S4. MS/MS spectrum of detected TAN 1169A (**3**) from the molecular network of interactions amongst endophytic microorganisms from *L. ericoides*.

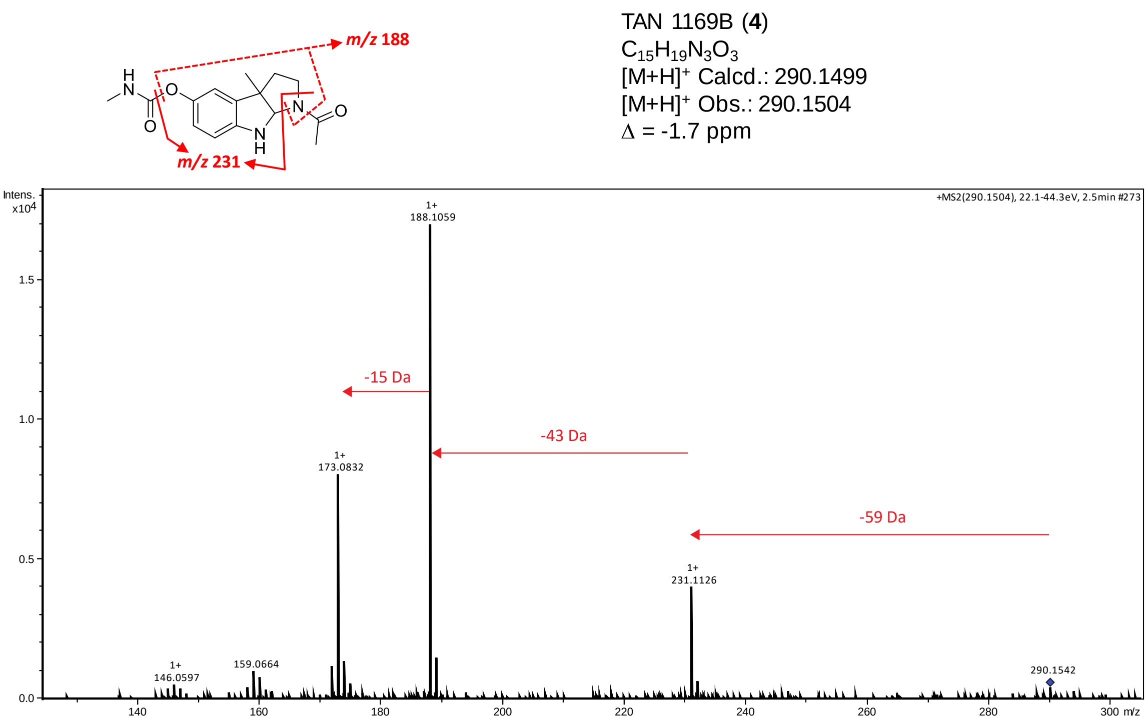

Figure S5. MS/MS spectrum of detected TAN 1169B (**4**) from the molecular network of interactions amongst endophytic microorganisms from *L. ericoides*.

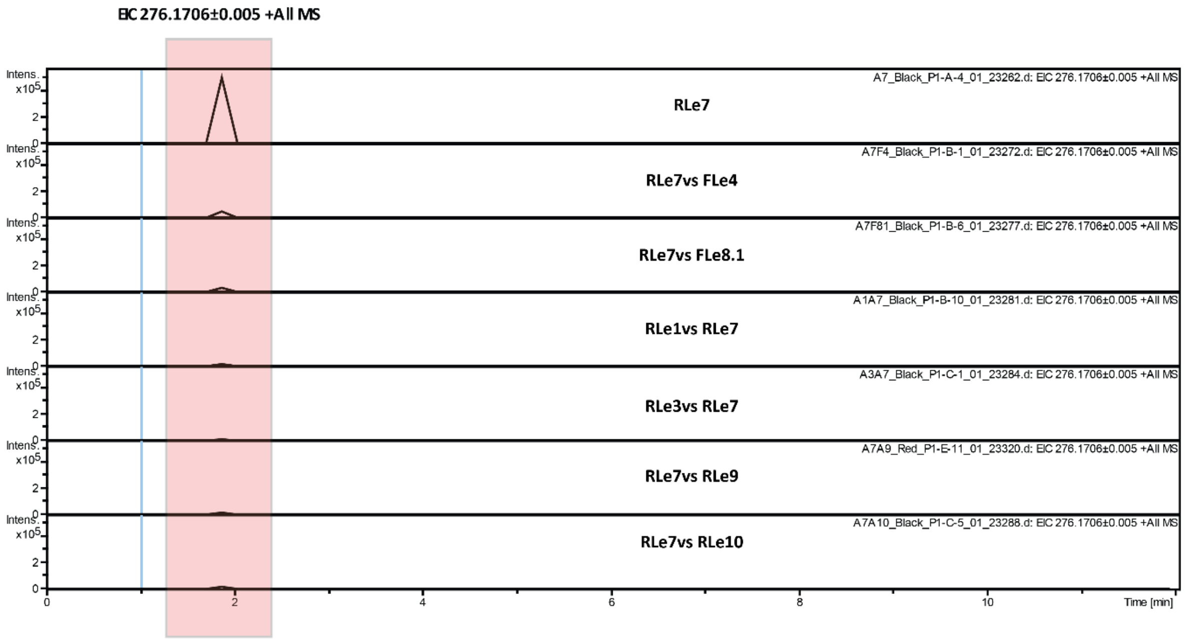

Figure S6. Extracted ion chromatogram comparison for physostigmine (**2**)
Physostigmine, *m/z* 276, was produced by *S. albospinus* RLe7 and detected in both, mono- and co-cultures. However, during microbial interactions the levels of the detected ion were consistently lower when compared to mono-cultures. The EIC are representative from four replicates.

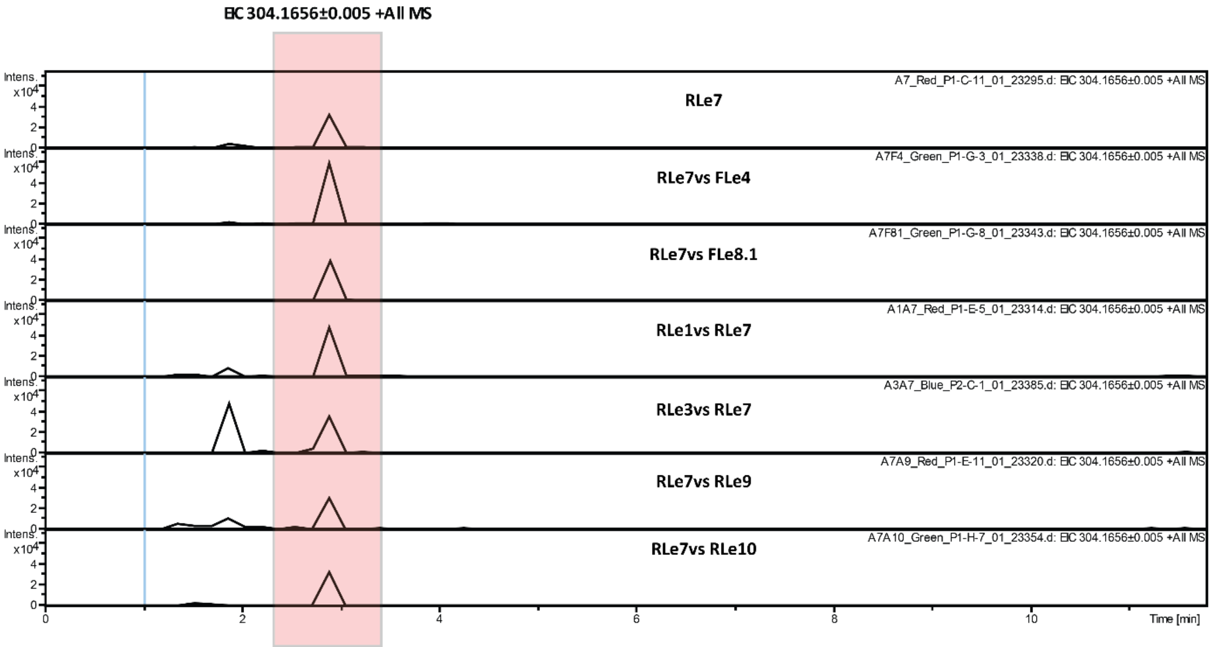

Figure S7. Extracted ion chromatogram comparison for TAN 1169A (**3**)
TAN 1169A, *m/z* 304, was produced by *S. albospinus* RLe7 and detected in both, mono- and co-cultures. The EIC are representative from four replicates.

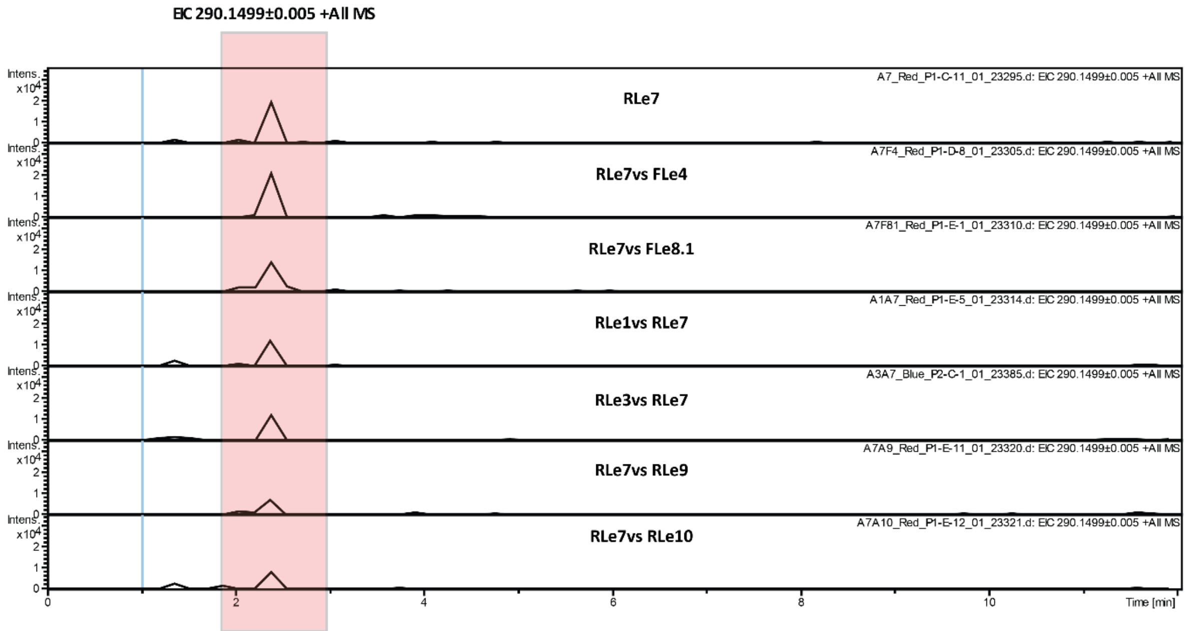

Figure S8. Extracted ion chromatogram comparison for TAN 1169B (**4**)
TAN 1169B, *m/z* 290, was produced by *S. albospinus* RLe7 and detected in both, mono- and co-cultures. The EIC are representative from four replicates.

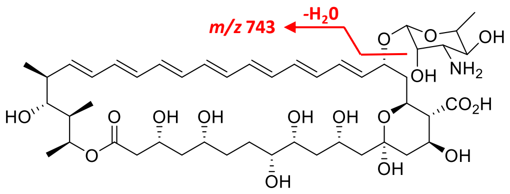

Amphotericin B (**1**)
$C_{47}H_{73}NO_{17}$
$[M+H]^+$ Calcd.: 924.4951
$[M+H]^+$ Obs.: 924.5007
Δ = - 6.0 ppm

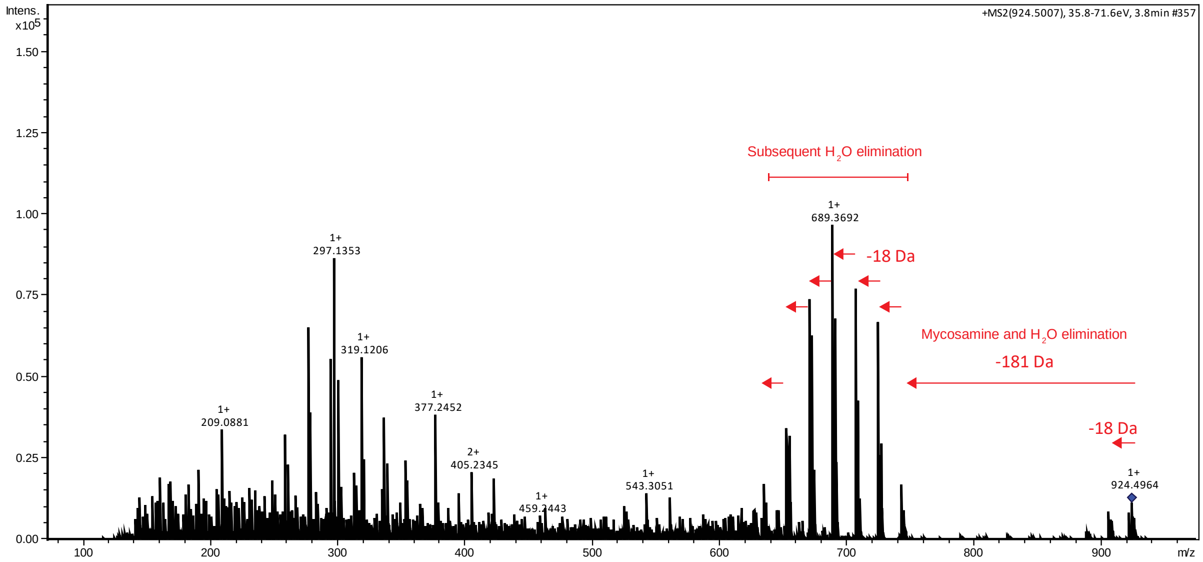

Figure S9. MS/MS spectrum of detected amphotericin B (**1**) from the molecular network of interactions amongst endophytic microorganisms from *L. ericoides.*

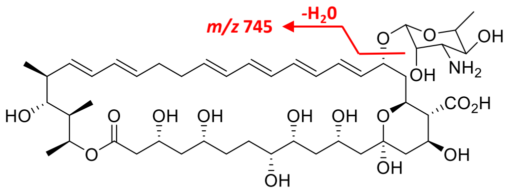

Amphotericin A (**5**)
$C_{47}H_{75}NO_{17}$
$[M+H]^+$ Calcd.: 926.5107
$[M+H]^+$ Obs.: 926.5162
Δ = -5.9 ppm

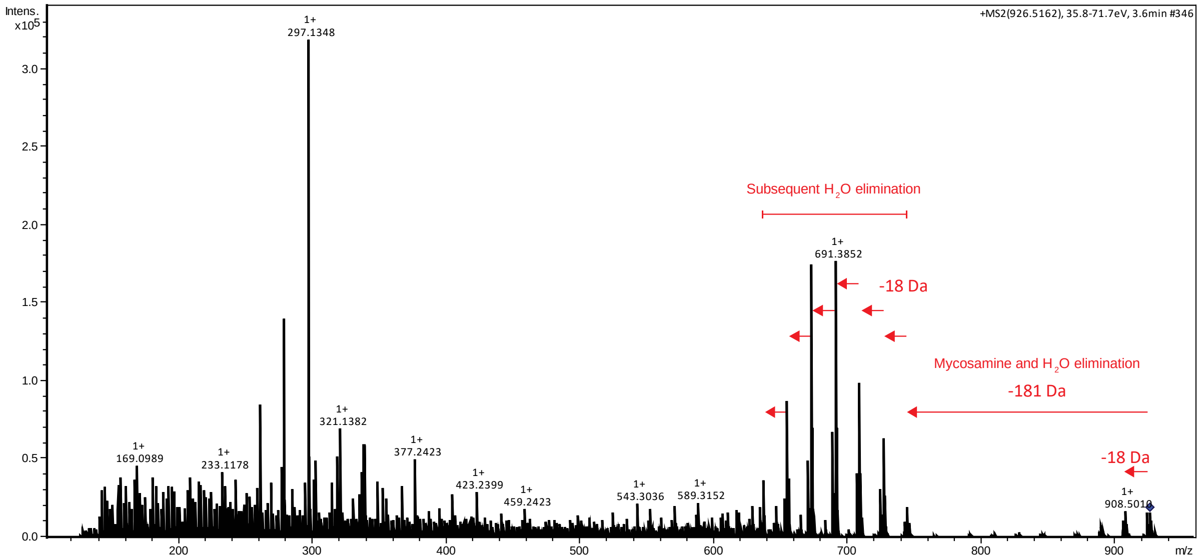

Figure S10. MS/MS spectrum of detected amphotericin A (**5**) from the molecular network of interactions amongst endophytic microorganisms from *L. ericoides.*

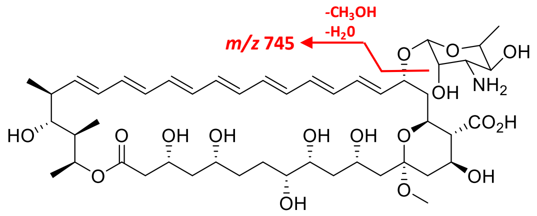

Amphotericin X or B2 (**6**)
$C_{48}H_{75}NO_{17}$
$[M+H]^+$ Calcd.: 938.5108
$[M+H]^+$ Obs.: 938.5142
Δ = - 3.6 ppm

Ampothericin X or B2 as representative annotation

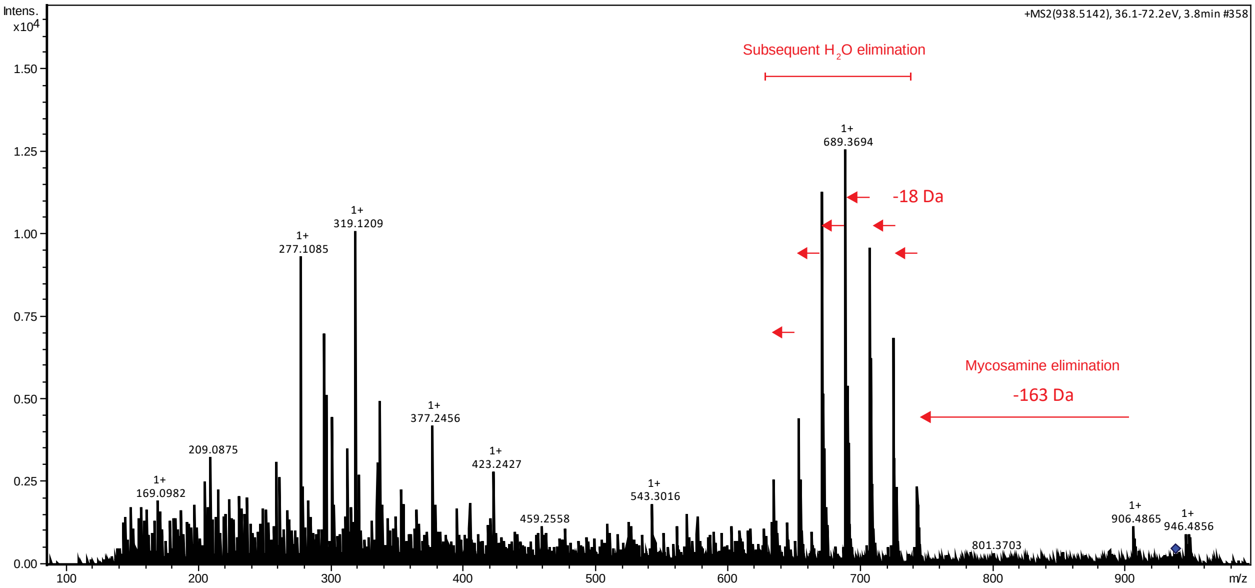

Figure S11. MS/MS spectrum of putative amphotericin X or B2 (**6**) from molecular networking of interactions amongst endophytic microorganisms from *L. ericoides*.

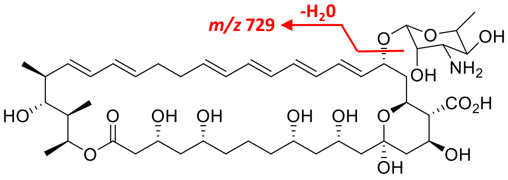

8-Deoxyampothericin A as representative annotation

Putative deoxyamphotericin A (**12**)
$C_{47}H_{75}NO_{16}$
$[M+H]^+$ Calcd.: 910.5158
$[M+H]^+$ Obs.: 910.5151
Δ = 0.8 ppm

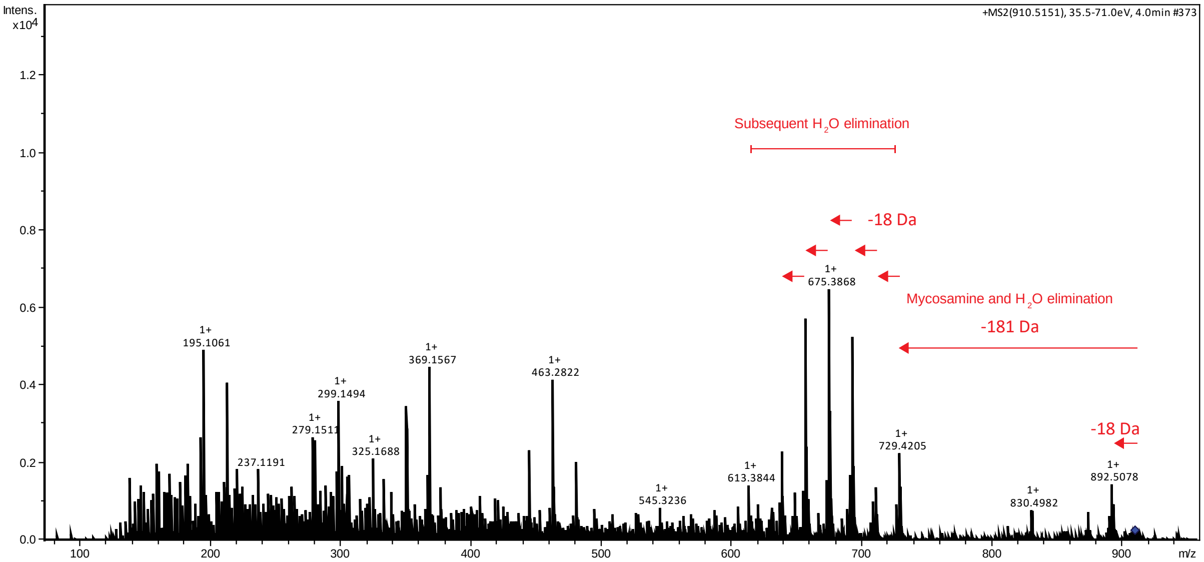

Figure S12. MS/MS spectrum of putative deoxyamphotericin A (**12**) from the molecular network of interactions amongst endophytic microorganisms from *L. ericoides*.

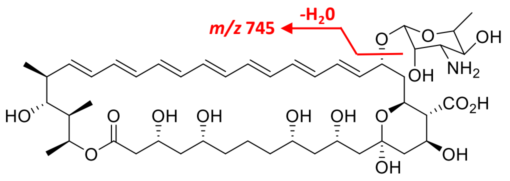

Putative deoxyamphotericin B (**13**)
$C_{47}H_{73}NO_{16}$
$[M+H]^+$ Calcd.: 908.5002
$[M+H]^+$ Obs.: 908.5056
Δ = -5.9 ppm

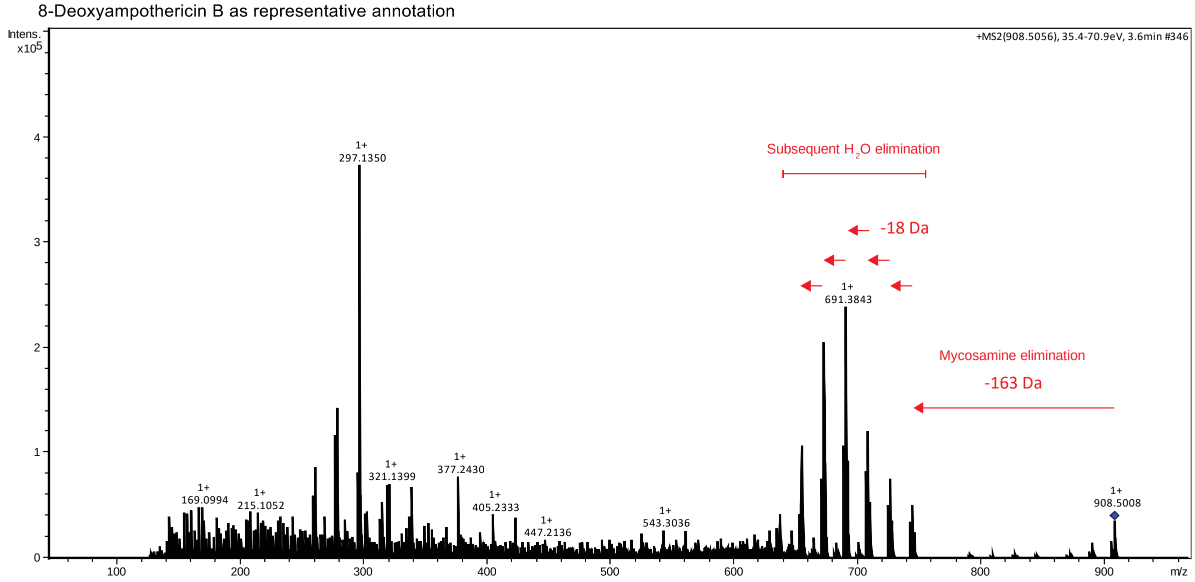

Figure S13. MS/MS spectrum of putative deoxyamphotericin B (**19**) from the molecular network of interactions amongst endophytic microorganisms from *L. ericoides*.

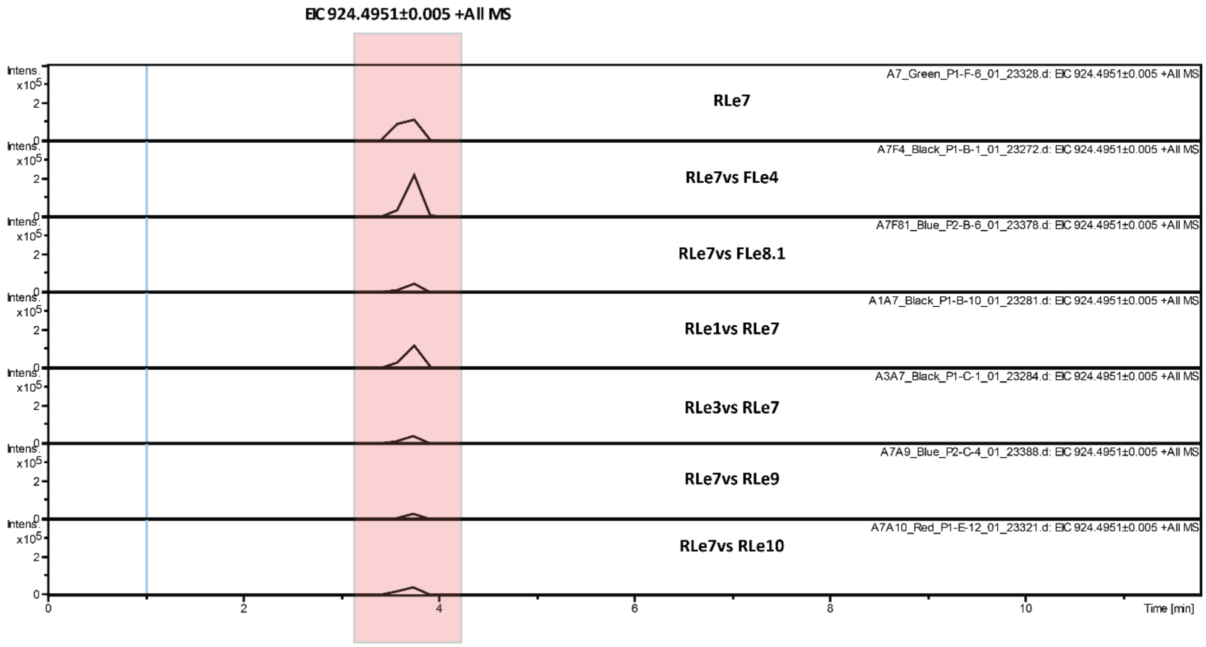

Figure S14. Extracted ion chromatogram comparison for compound (**1**)
Amphotericin B, *m/z* 924, was produced by *S. albospinus* RLe7 and detected in both, mono- and co-cultures. The EIC are representative from four replicates.

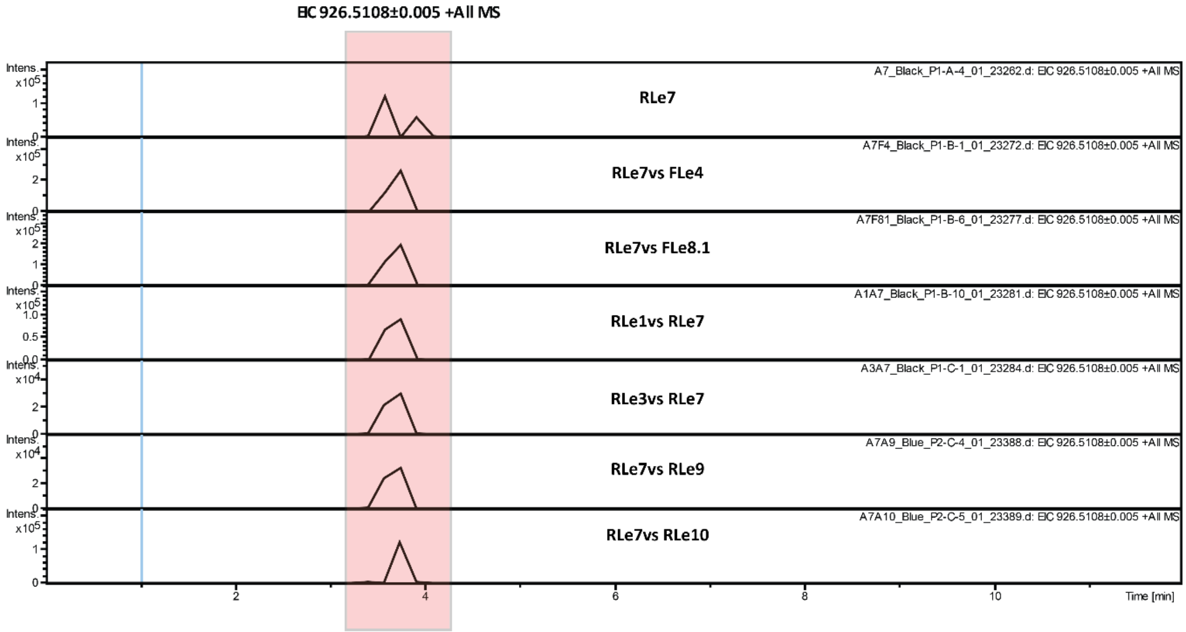

Figure S15. Extracted ion chromatogram comparison for compound (**5**)
Amphotericin B, *m/z* 926, was produced by *S. albospinus* RLe7 and detected in both, mono- and co-cultures. The EIC are representative from four replicates.

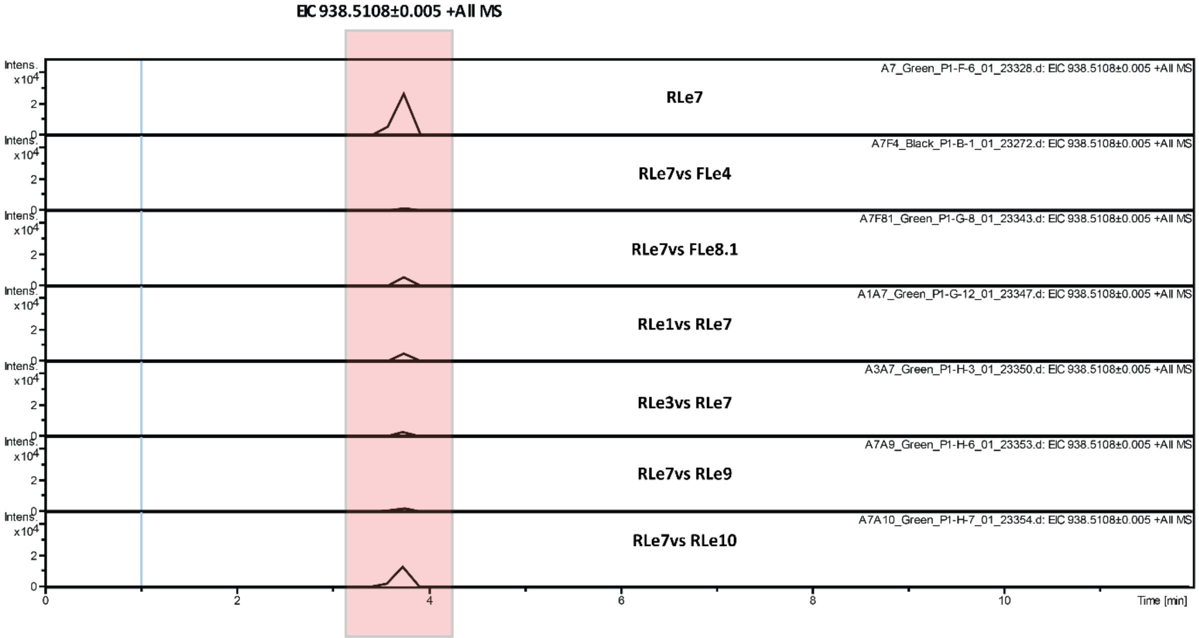

Figure S16. Extracted ion chromatogram comparison for compound (**6**)
Amphotericin X or B_2, *m/z* 938, was produced by *S. albospinus* RLe7 and detected in both, mono- and co-cultures. The EIC are representative from four replicates.

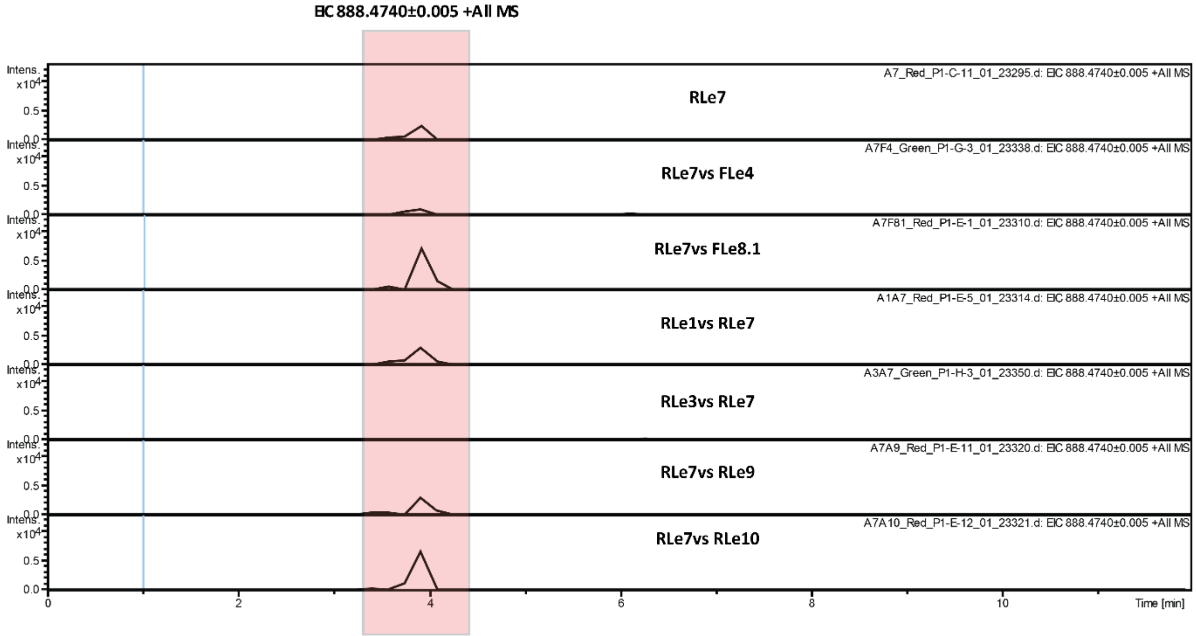

Figure S17. Extracted ion chromatogram comparison for compound (**7**)
Compound **7**, *m/z* 888, was produced by *S. albospinus* RLe7 and detected in both, mono- and co-cultures. The EIC are representative from four replicates.

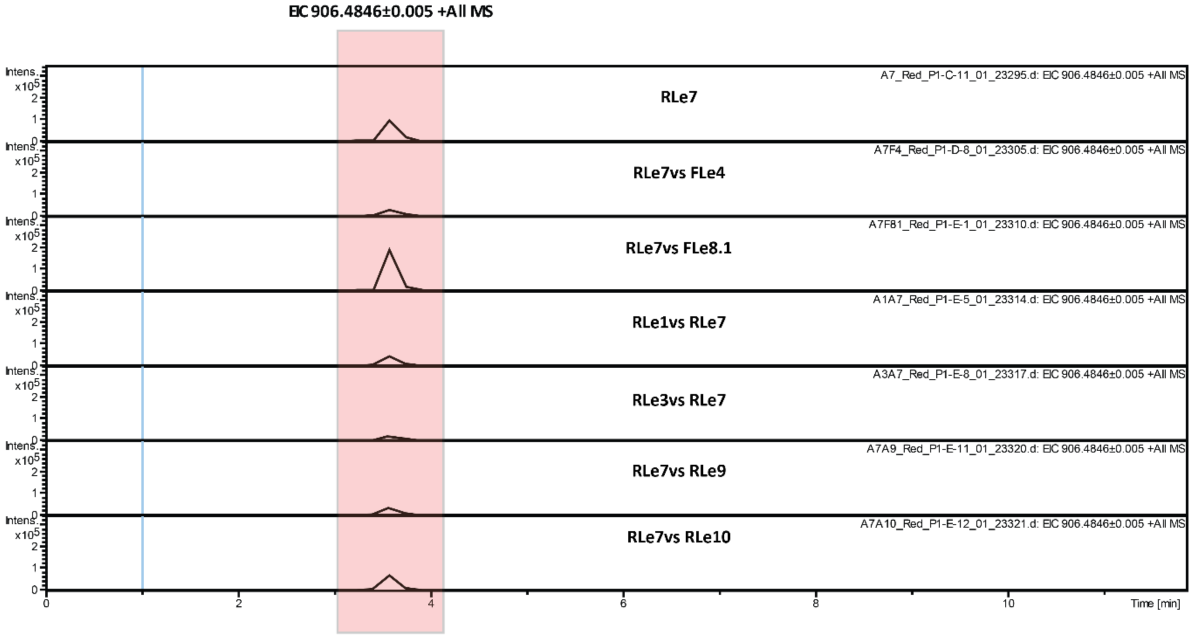

Figure S18. Extracted ion chromatogram comparison for compound (**8**)
Compound **8**, *m/z* 906, was produced by *S. albospinus* RLe7 and detected in both, mono- and co-cultures. The EIC are representative from four replicates.

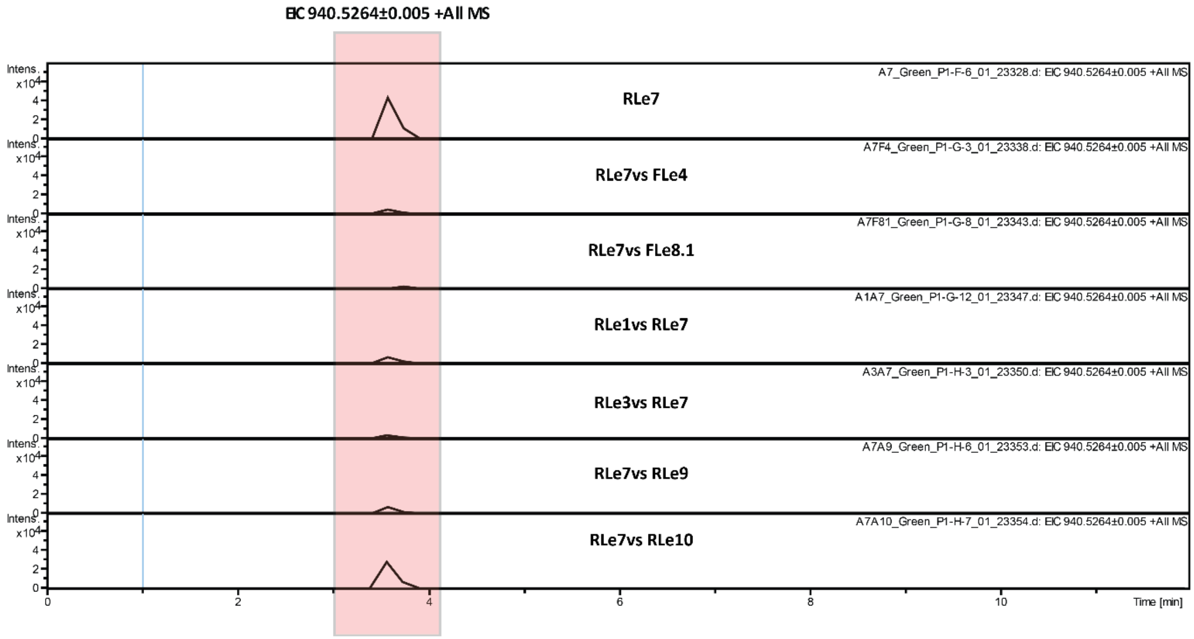

Figure S19. Extracted ion chromatogram comparison for compound (**9**)
Compound **9**, *m/z* 940, was produced by *S. albospinus* RLe7 and detected in both, mono- and co-cultures. The EIC are representative from four replicates.

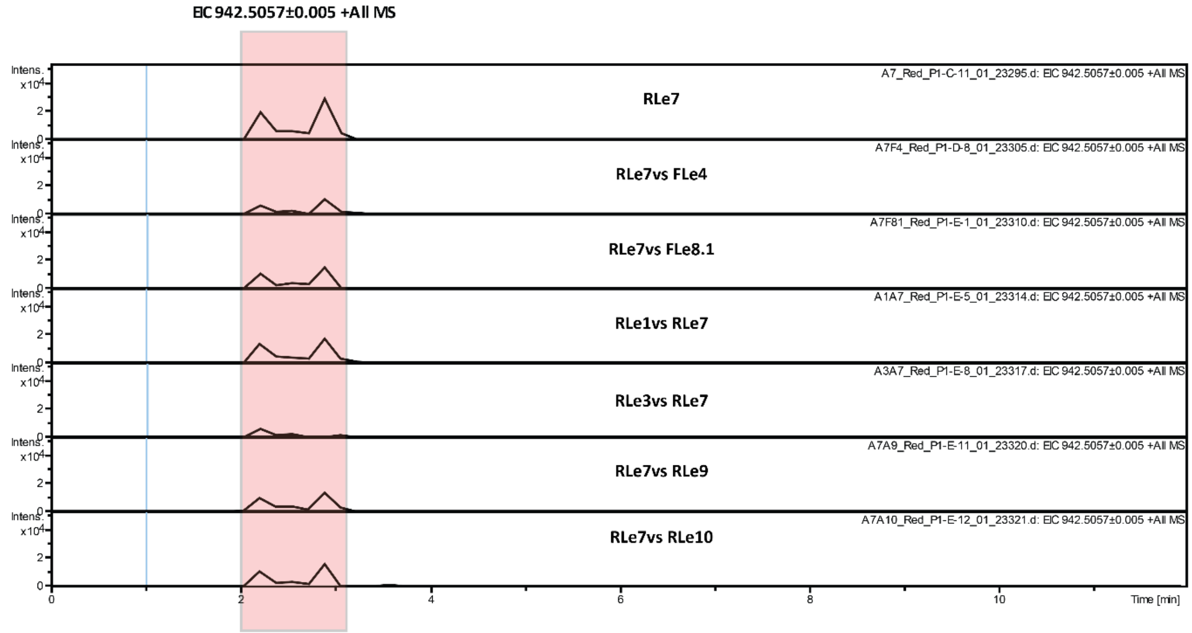

Figure S20. Extracted ion chromatogram comparison for compound (**10**)
Compound **10**, *m/z* 942, was produced by *S. albospinus* RLe7 and detected in both, mono- and co-cultures. The EIC are representative from four replicates.

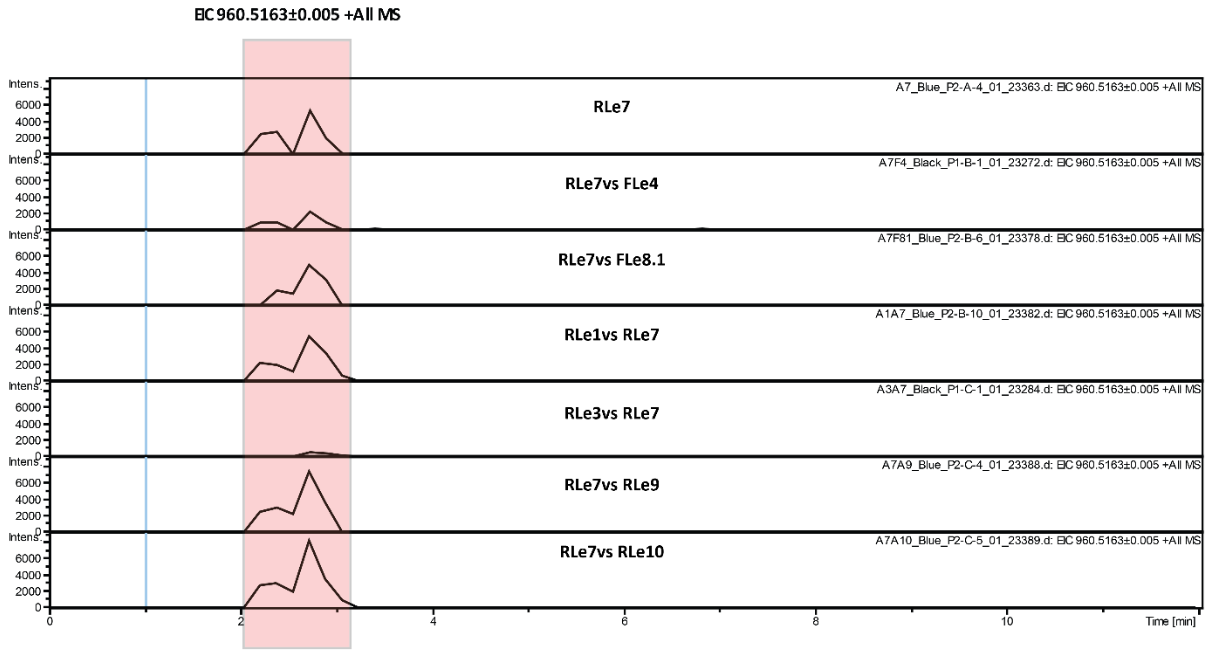

Figure S21. Extracted ion chromatogram comparison for compound (**11**)
Compound **11**, *m/z* 960, was produced by *S. albospinus* RLe7 and detected in both, mono- and co-cultures. The EIC are representative from four replicates.

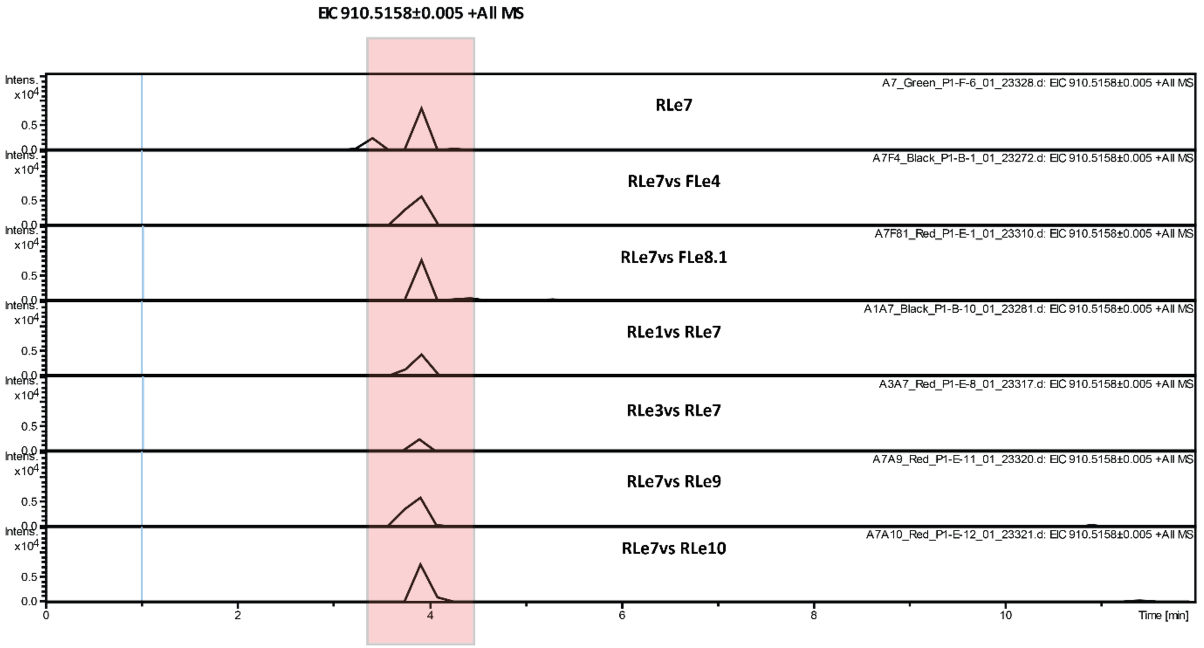

Figure S22. Extracted ion chromatogram comparison for compound (**12**)
Compound **12**, *m/z* 910, was produced by *S. albospinus* RLe7 and detected in both, mono- and co-cultures. The EIC are representative from four replicates.

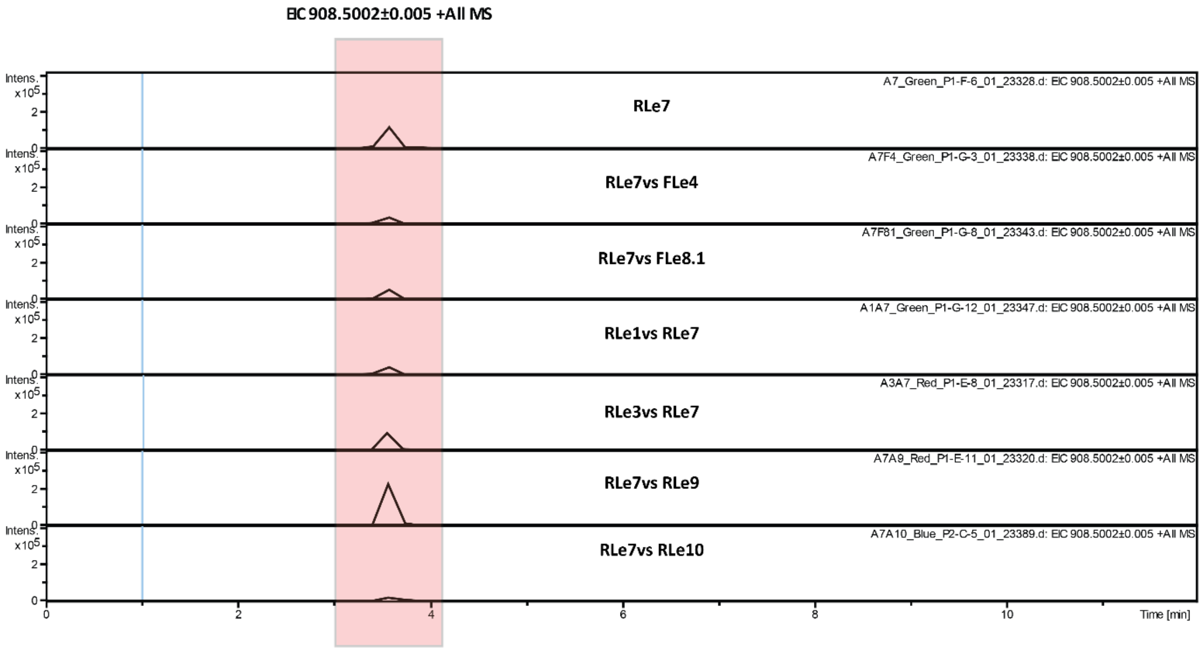

Figure S23. Extracted ion chromatogram comparison for compound (**13**)
Compound **13**, *m/z* 908, was produced by *S. albospinus* RLe7 and detected in both, mono- and co-cultures. The EIC are representative from four replicates.

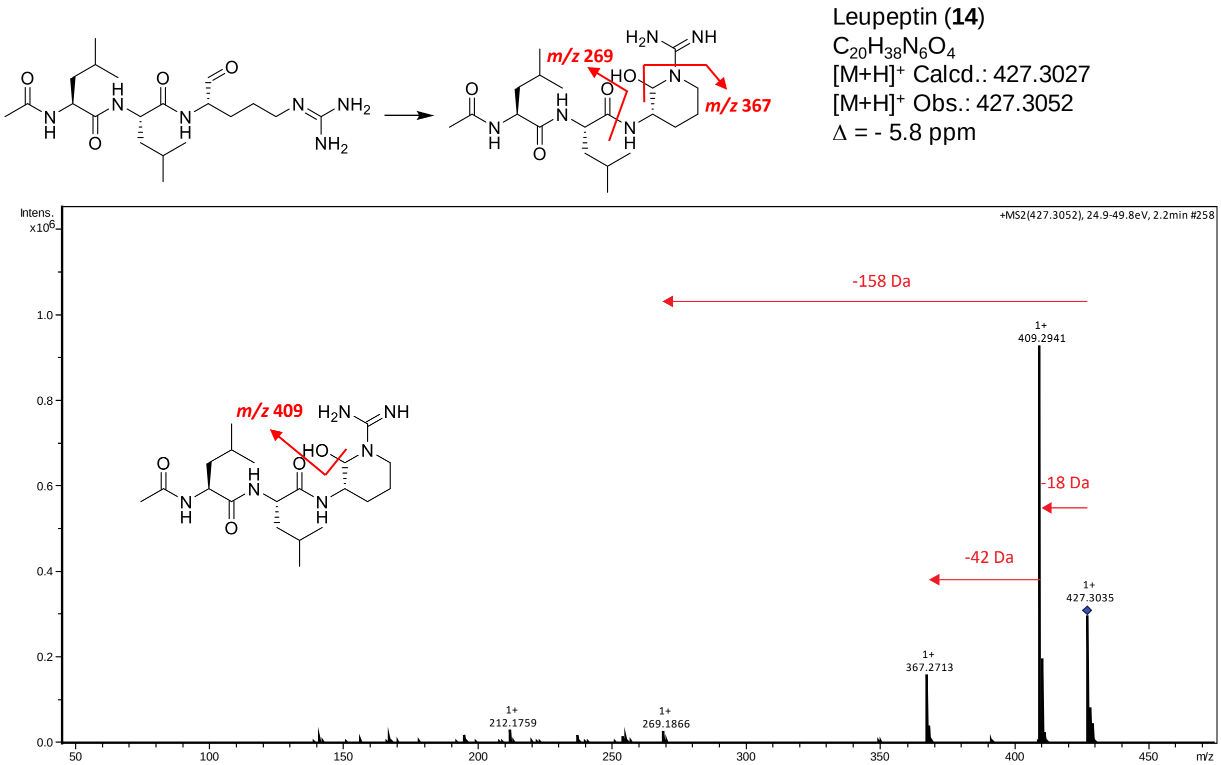

Figure S24. MS/MS spectrum of detected leupeptin (**14**) from the molecular network of interactions amongst endophytic microorganisms from *L. ericoides*.

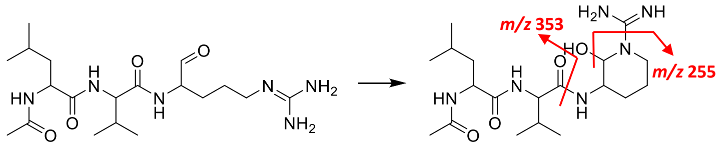

N-Acetylleucylvalylargininal as representative annotation

Acetyl-(LVR (**15**) or VLR) (**16**)
$C_{19}H_{36}N_6O_4$
$[M+H]^+$ Calcd.: 413.2871
$[M+H]^+$ Obs.: 413.2878
Δ = -1.7 ppm

No differentiation between N-acetylleucylvalylargininal and N-acetylvalylleucylargininal in this study

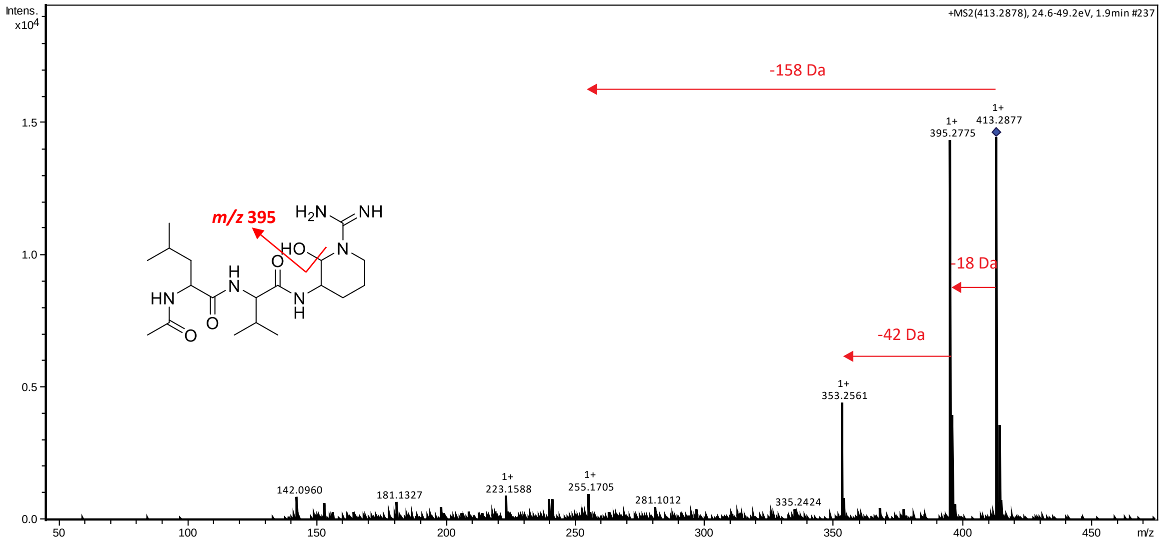

Figure S25. MS/MS spectrum of detected leupeptin acetyl-(LVR (**15**) or VLR (**16**)) from the molecular network of interactions amongst endophytic microorganisms from *L. ericoides*.

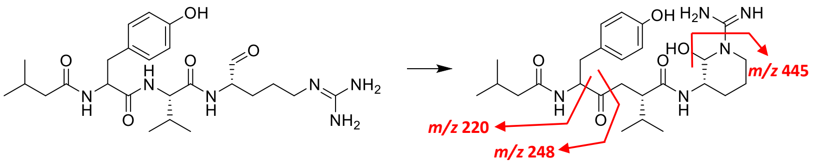

Strepin P1 (**17**)
$C_{25}H_{40}N_6O_5$
$[M+H]^+$ Calcd.: 505.3133
$[M+H]^+$ Obs.: 505.3102
Δ = 6.1 ppm

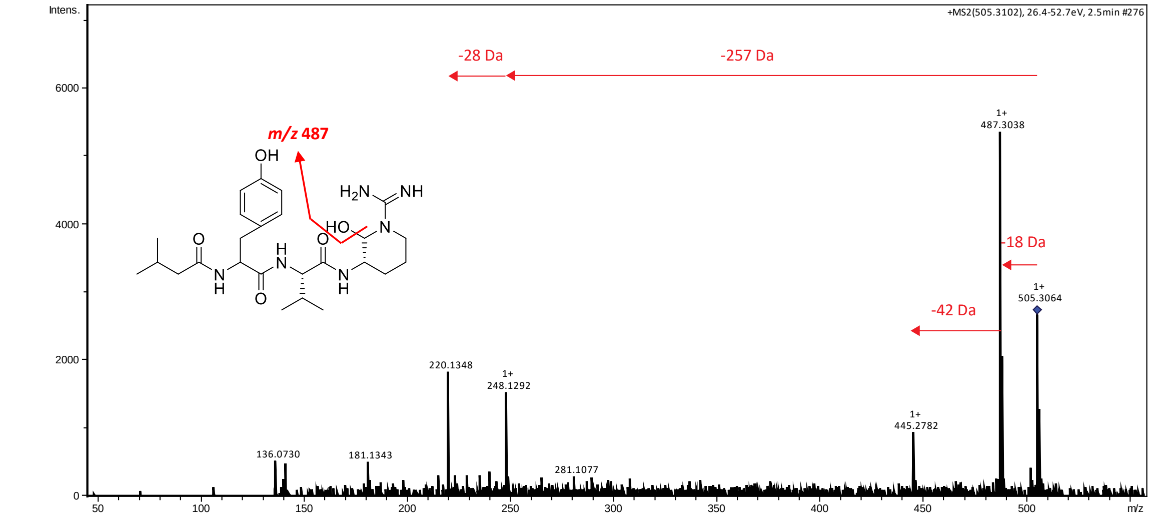

Figure S26. MS/MS spectrum of detected strepin P1 (**17**) from molecular network of interactions amongst endophytic microorganisms from *L. ericoides*.

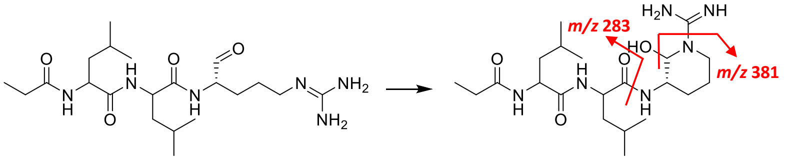

Leupeptin Pr-LL (**18**)
$C_{21}H_{40}N_6O_4$
$[M+H]^+$ Calcd.: 441.3184
$[M+H]^+$ Obs.: 441.3197
Δ = - 3.0 ppm

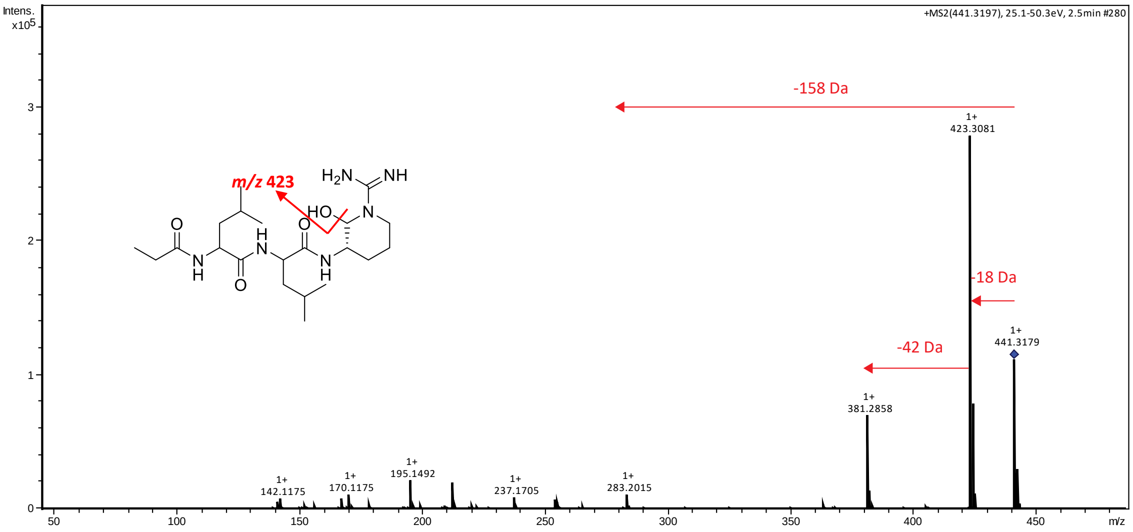

Figure S27. MS/MS spectrum of detected leupeptin Pr-LL (**18**) from molecular network of interactions amongst endophytic microorganisms from *L. ericoides*.

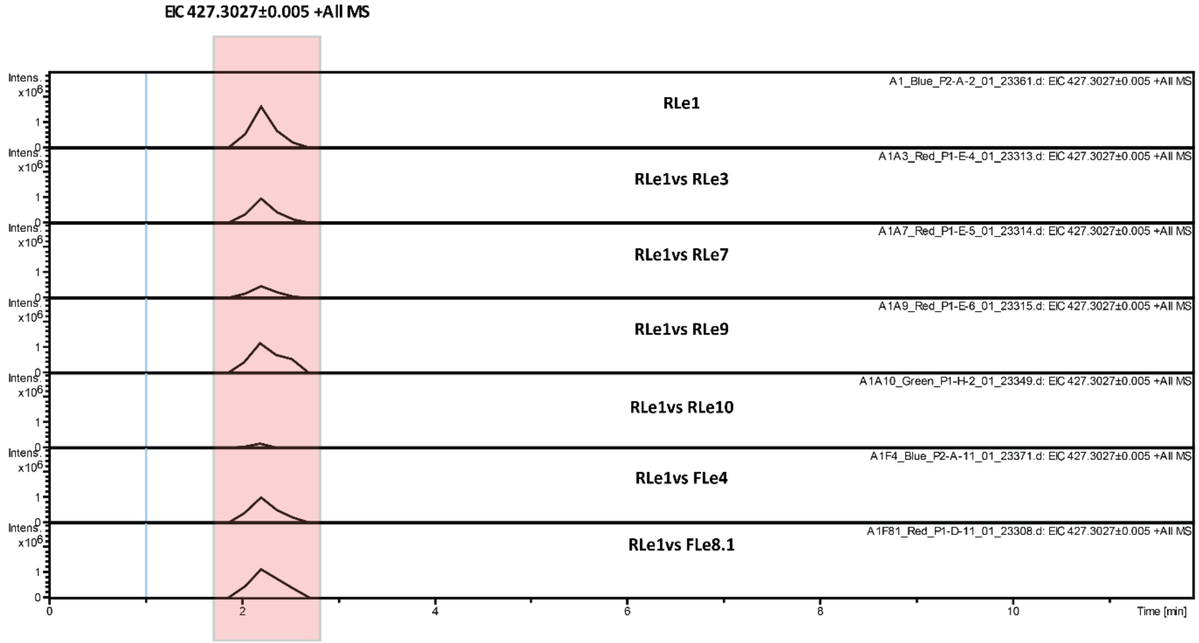

Figure S28. Extracted ion chromatogram comparison for compound (**14**) from mono- and co-cultures involving *S. cattleya* RLe1

Leupeptin **14**, *m/z* 427, was produced by *S. cattleya* RLe1 and *S. albospinus* RLe7 and detected in both, mono- and co-cultures. The EIC are representative from four replicates.

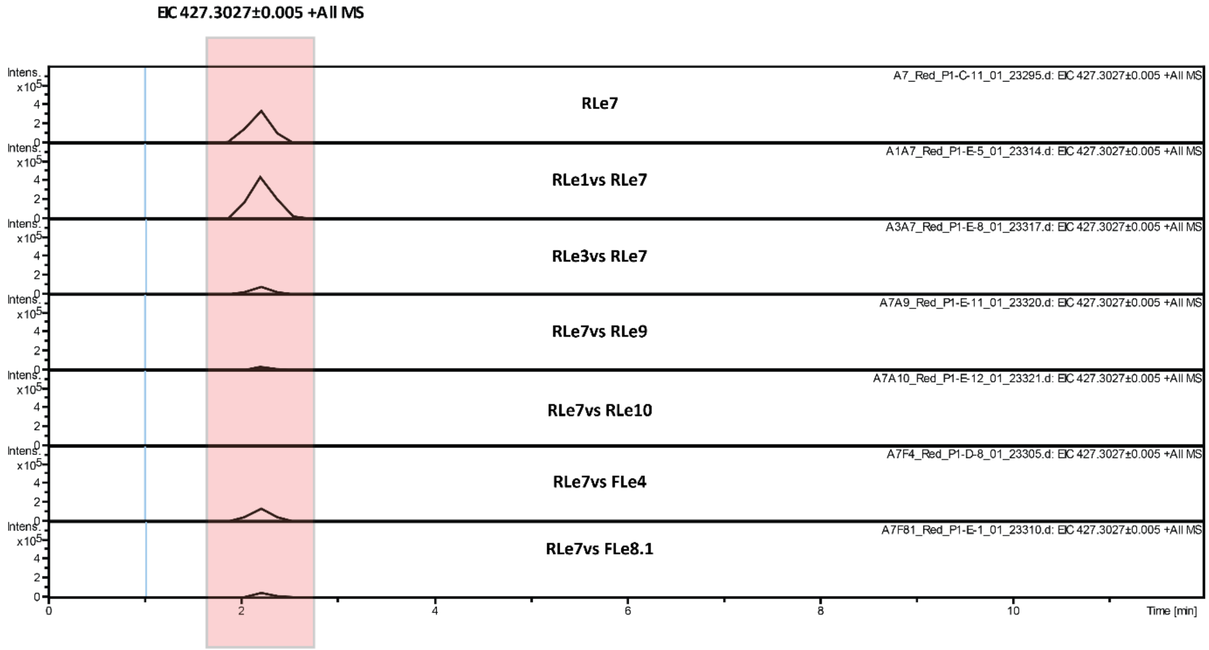

Figure S29. Extracted ion chromatogram comparison for compound (**14**) from mono- and co-cultures involving *S. albospinus* RLe7

Leupeptin **14**, *m/z* 427, was produced by *S. cattleya* RLe1 and *S. albospinus* RLe7 and detected in both, mono- and co-cultures. The EIC are representative from four replicates.

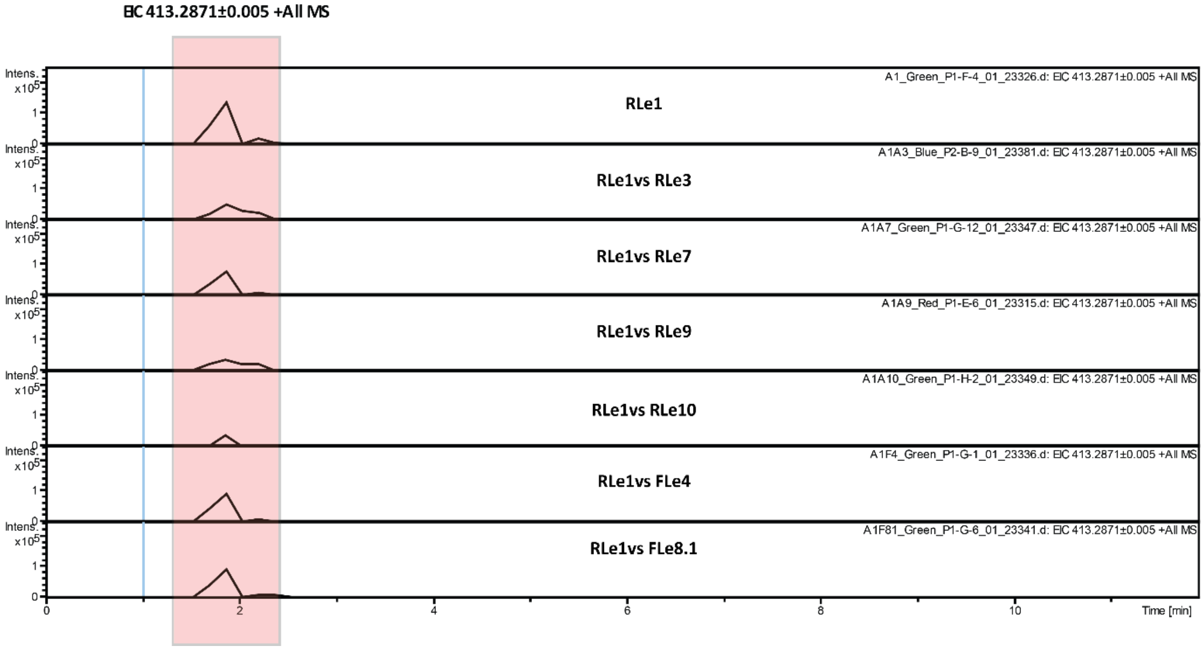

Figure S30. Extracted ion chromatogram comparison for compound (**15**/**16**) from mono- and co-cultures involving *S. cattleya* RLe1

Leupeptin analogue **15**/**16**, *m/z* 413, was produced by *S. cattleya* RLe1 and *S. albospinus* RLe7 and detected in both, mono- and co-cultures. The EIC are representative from four replicates.

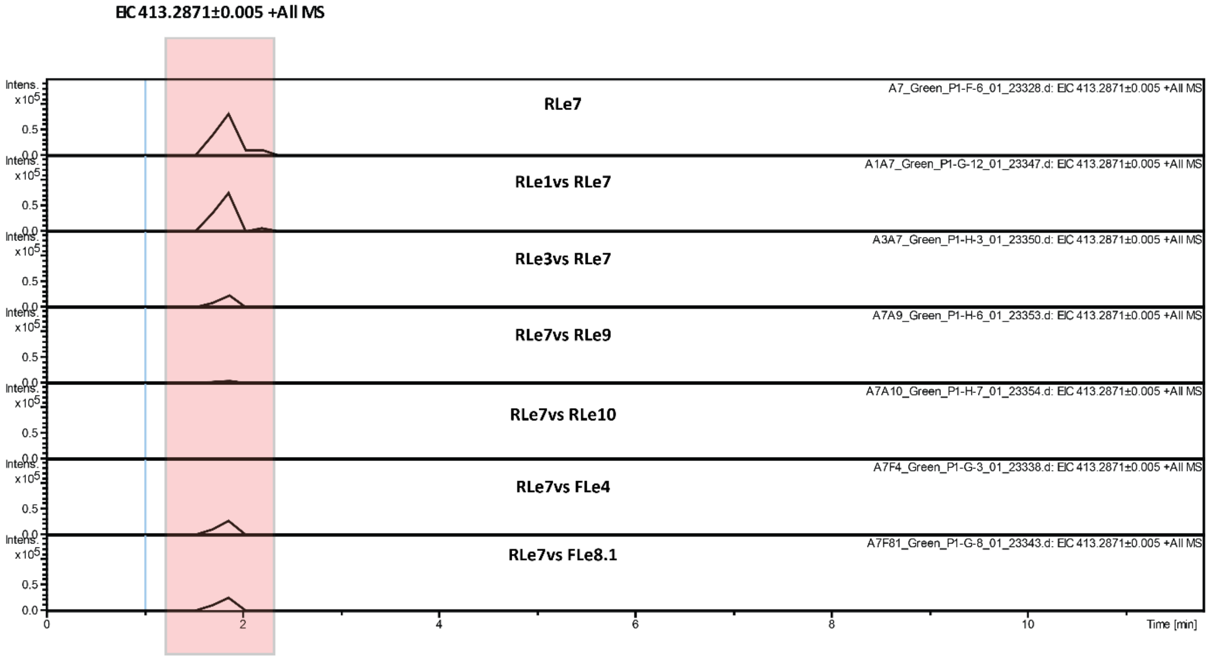

Figure S31. Extracted ion chromatogram comparison for compound (**15**/**16**) from mono- and co-cultures involving *S. albospinus* RLe7

Leupeptin analogue **15**/**16**, *m/z* 413, was produced by *S. cattleya* RLe1 and *S. albospinus* RLe7 and detected in both, mono- and co-cultures. The EIC are representative from four replicates.

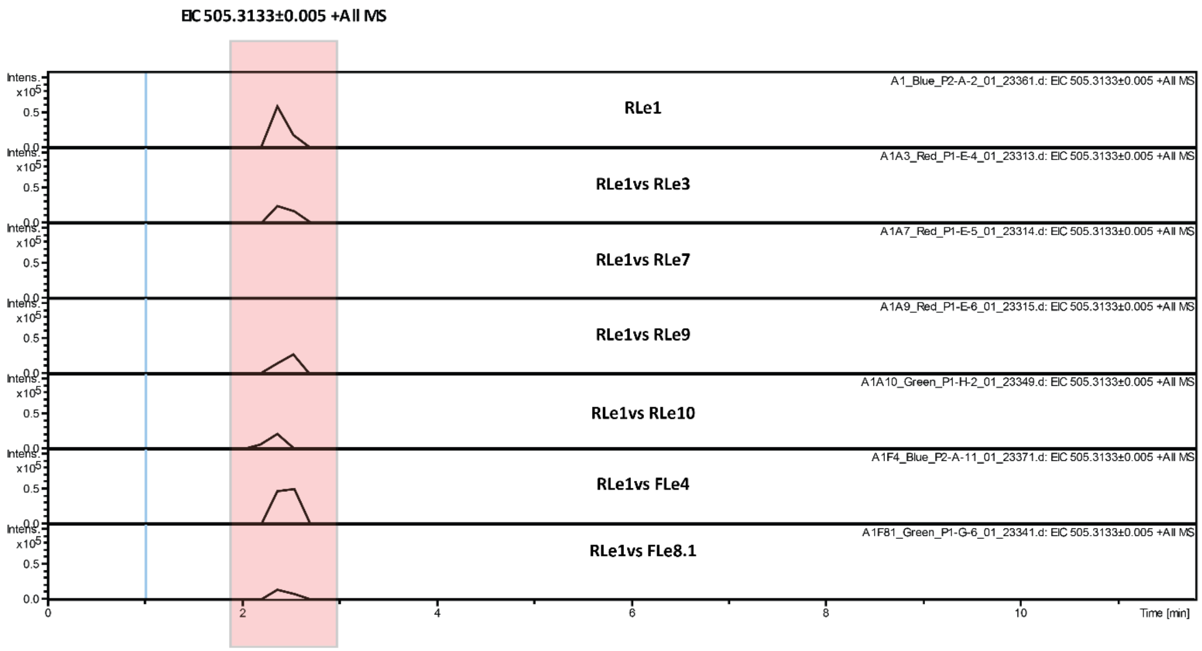

Figure S32. Extracted ion chromatogram comparison for compound (**17**) from mono- and co-cultures involving *S. cattleya* RLe1

Strepin P1 (**17**), *m/z* 505, was produced by *S. cattleya* RLe1 and *K. cystarginea* RLe10 and detected in both, mono- and co-cultures involving *S. cattleya* RLe1. The EIC are representative from four replicates.

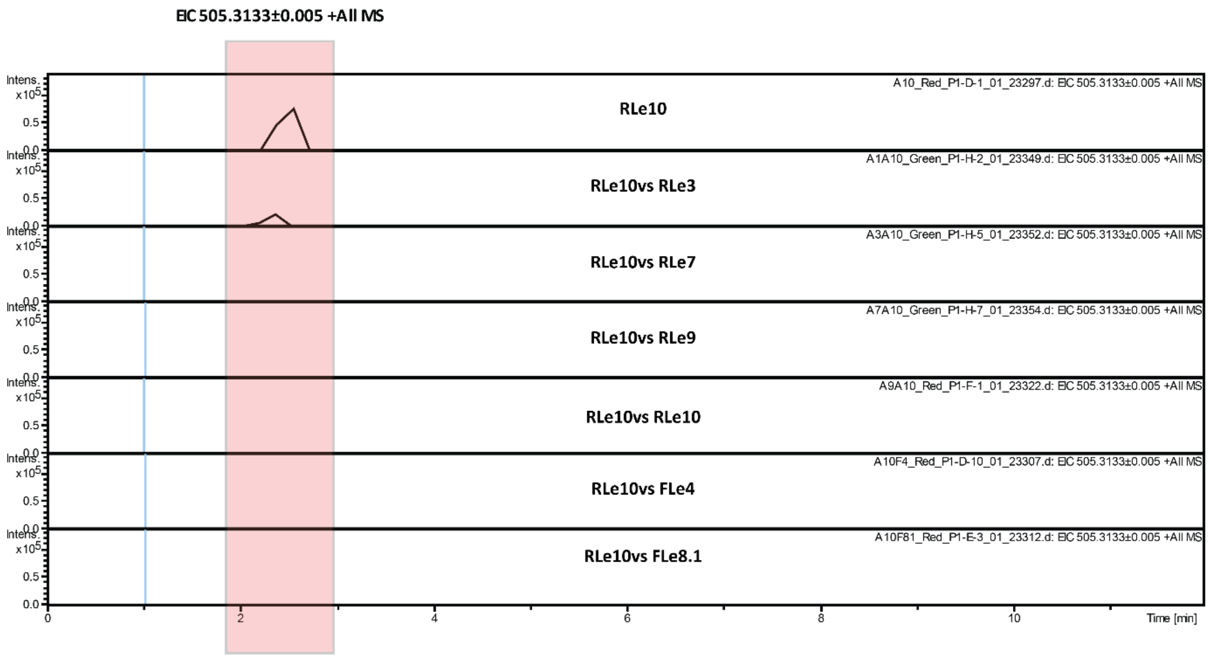

Figure S33. Extracted ion chromatogram comparison for compound (**17**) from mono- and co-cultures involving *K. cystarginea* RLe10

Strepin P1 (**17**), *m/z* 505, was also produced by *K. cystarginea* RLe10 and detected in mono-culture and only in co-cultures involving *S. mobaraensis* RLe3. The EIC are representative from four replicates.

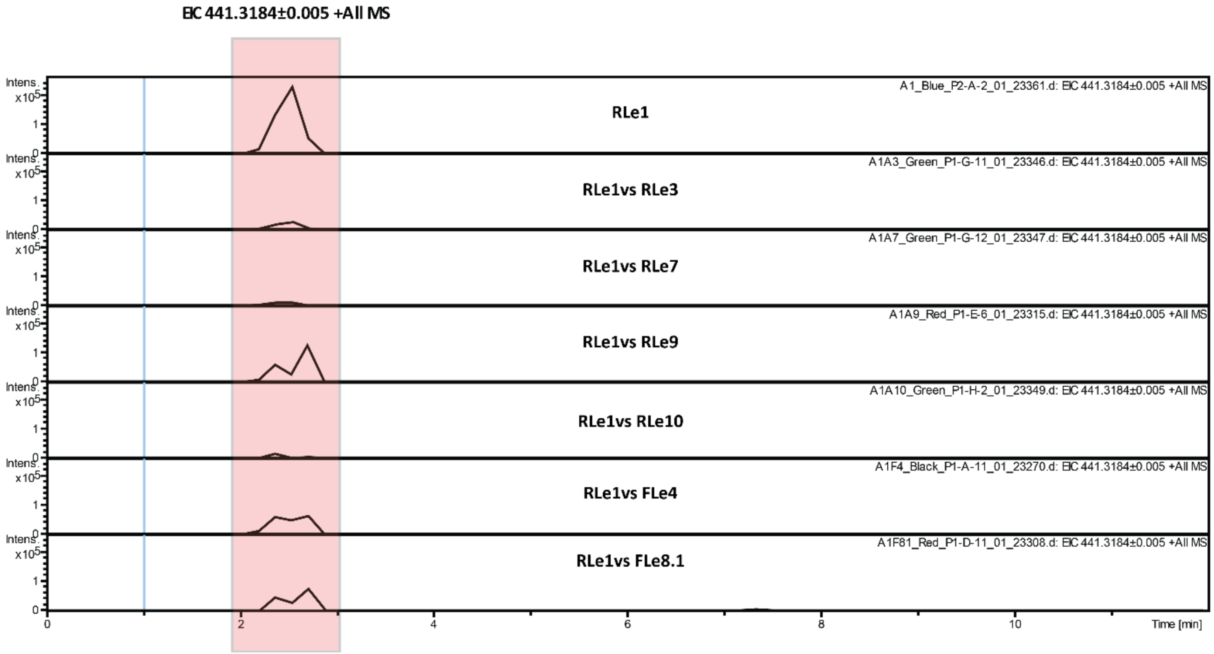

Figure S34. Extracted ion chromatogram comparison for compound (**18**)
Leupeptin Pr-LL (**18**), *m/z* 441, was produced by *S. cattleya* RLe1 and detected in mono- and co-cultures. The EIC are representative from four replicates.

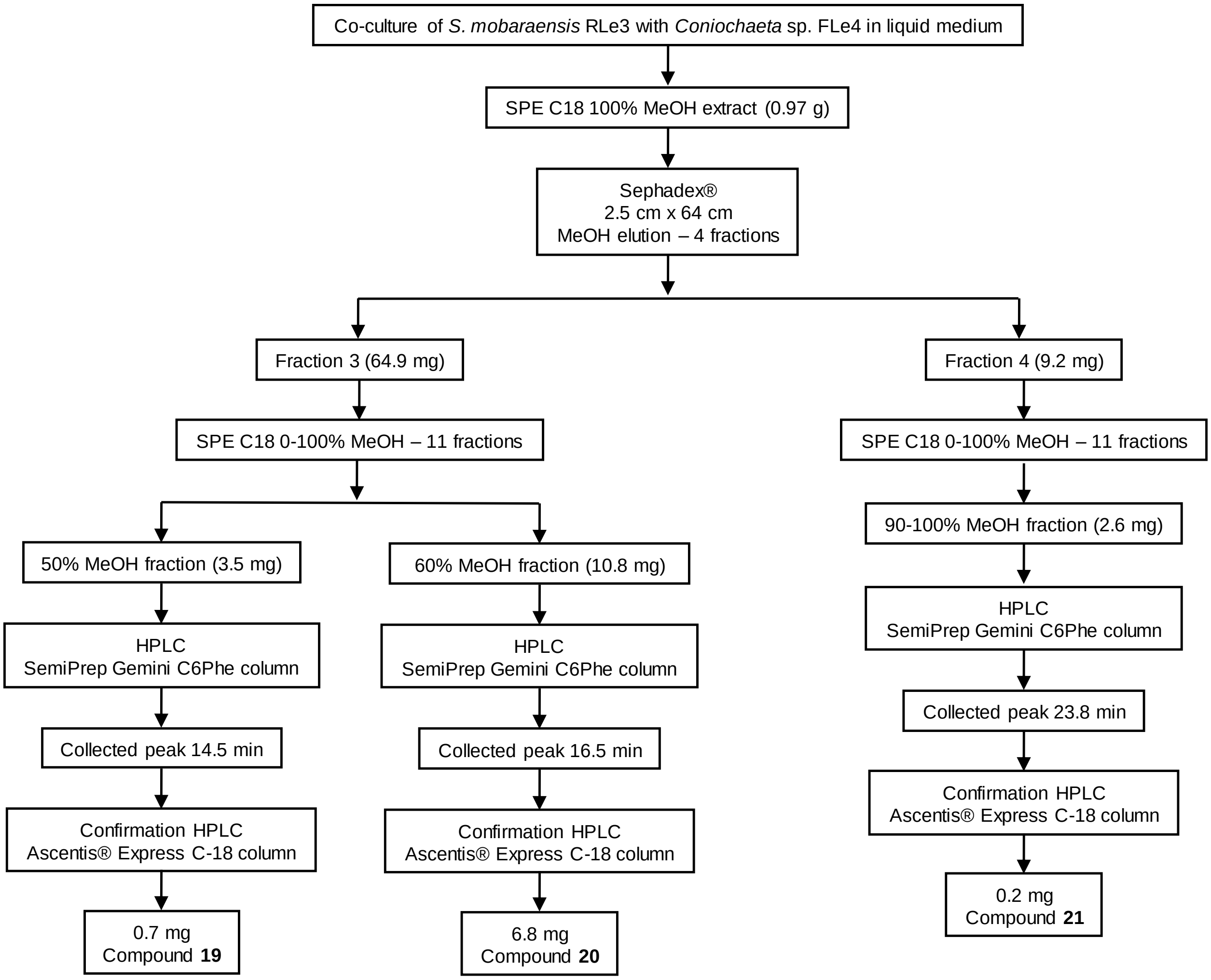

Figure S35. Purification workflow for compounds **19, 20** and **21**

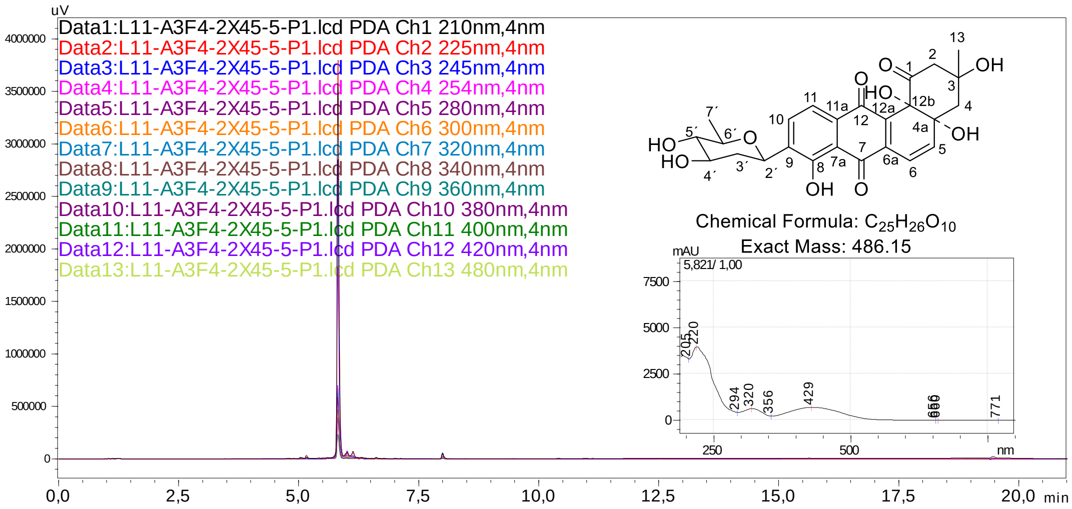

Figure S36. HPLC-DAD of purified peak corresponding to compound (**19**)

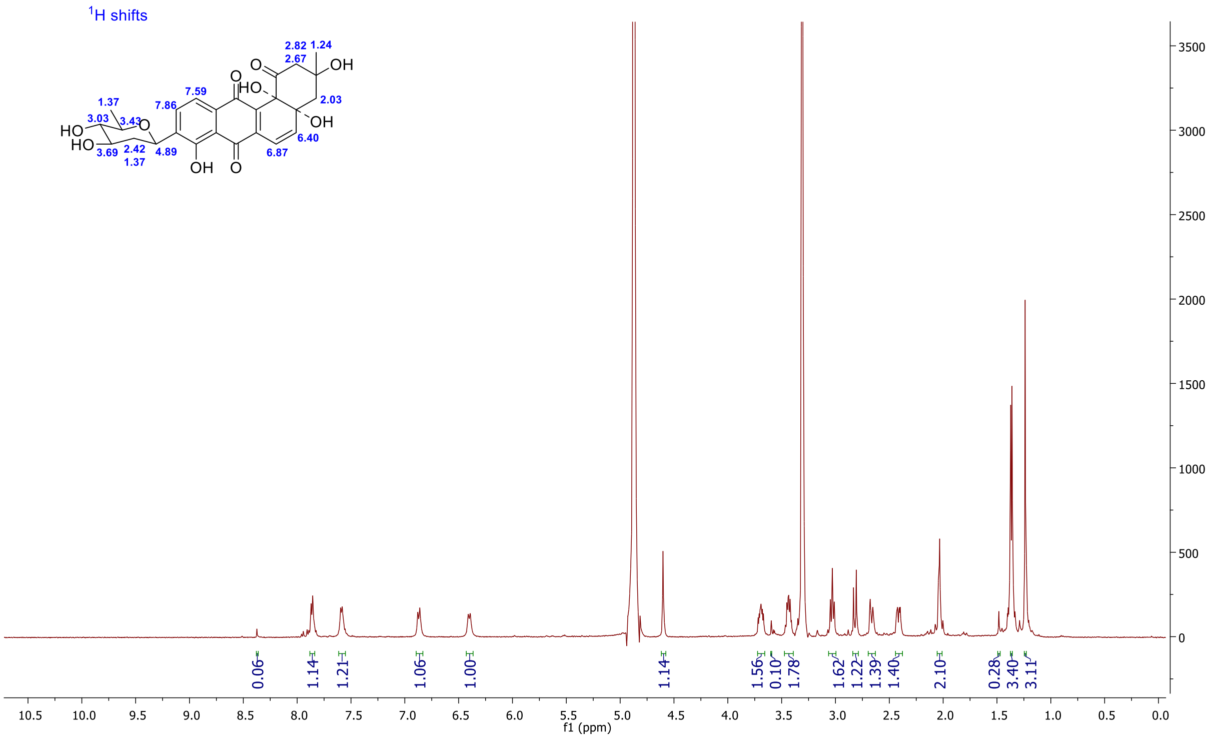

Figure S37. ^{1}H NMR (500 MHz, MeOH-d_4) spectrum of aquayamycin (**19**).

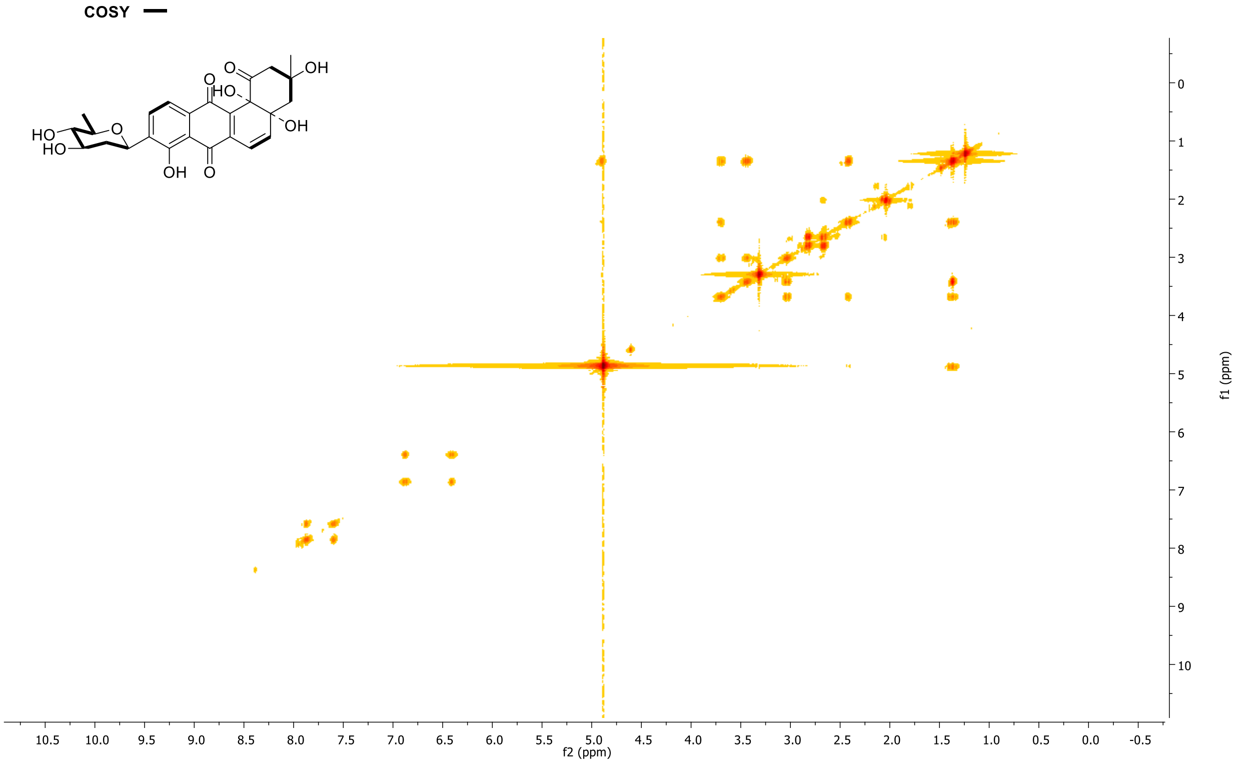

Figure S38. *g*COSY (500 MHz, MeOH-d_4) spectrum of aquayamycin (**19**).

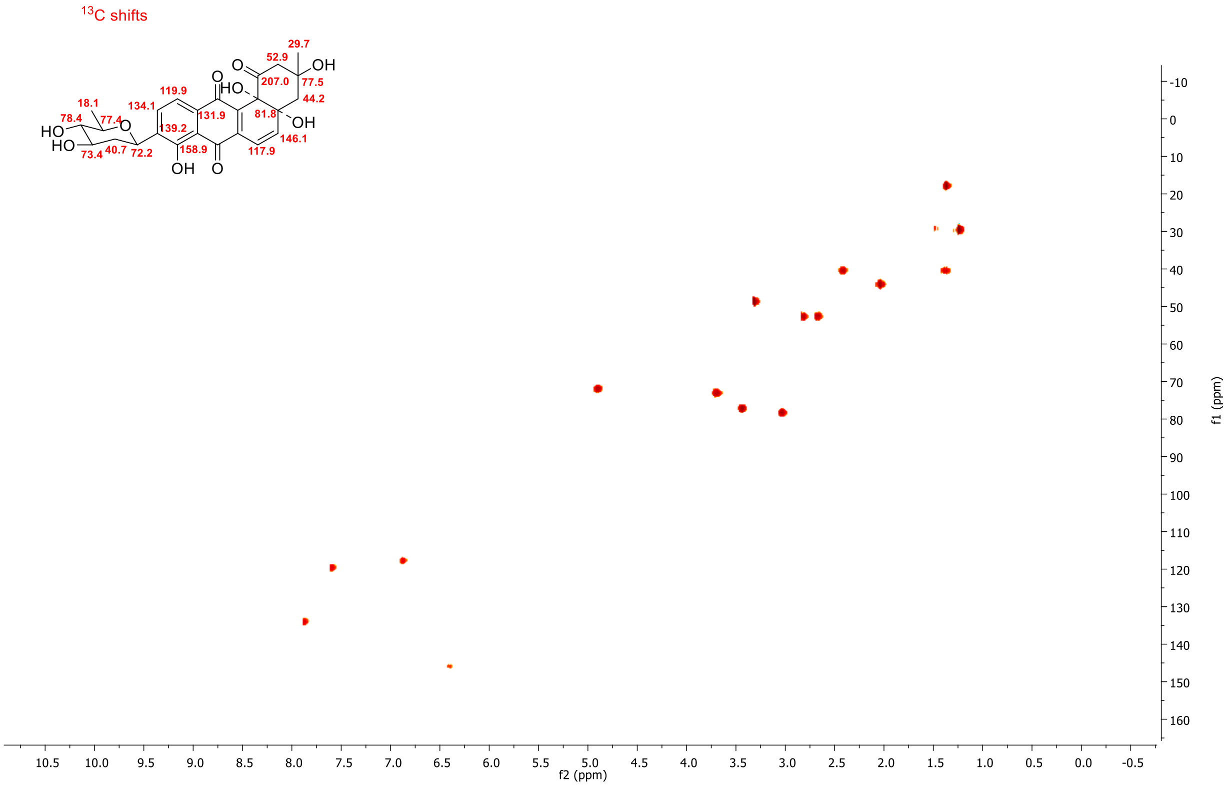

Figure S39. *g*HSQC (500 MHz, MeOH-d_4) spectrum of aquayamycin (**19**).

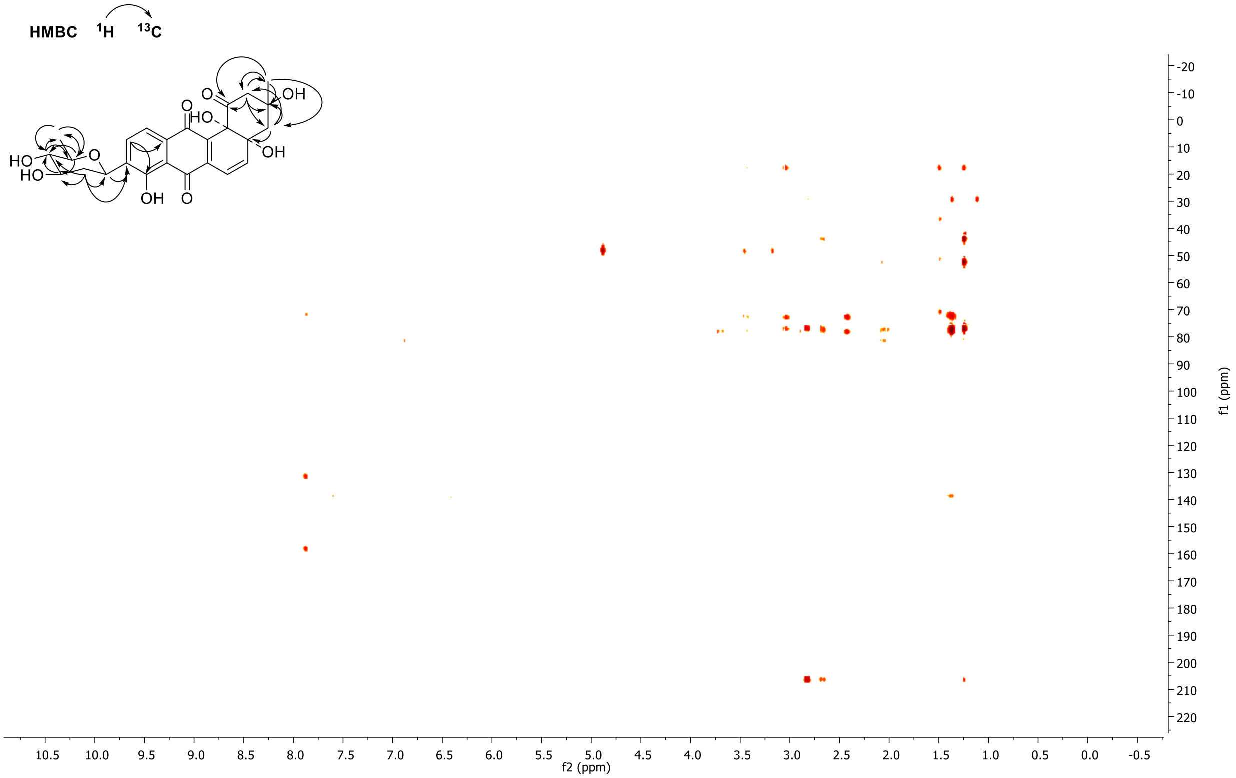

Figure S40. *g*HMBC (500 MHz, MeOH-d_4) spectrum of aquayamycin (**19**).

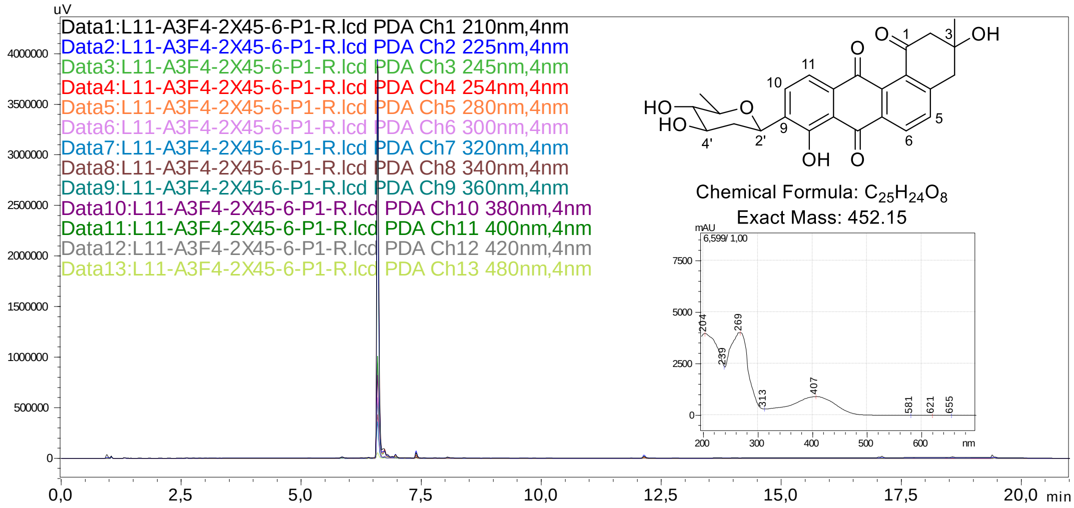

Figure S41. HPLC-DAD of purified peak corresponding to compound (**20**)

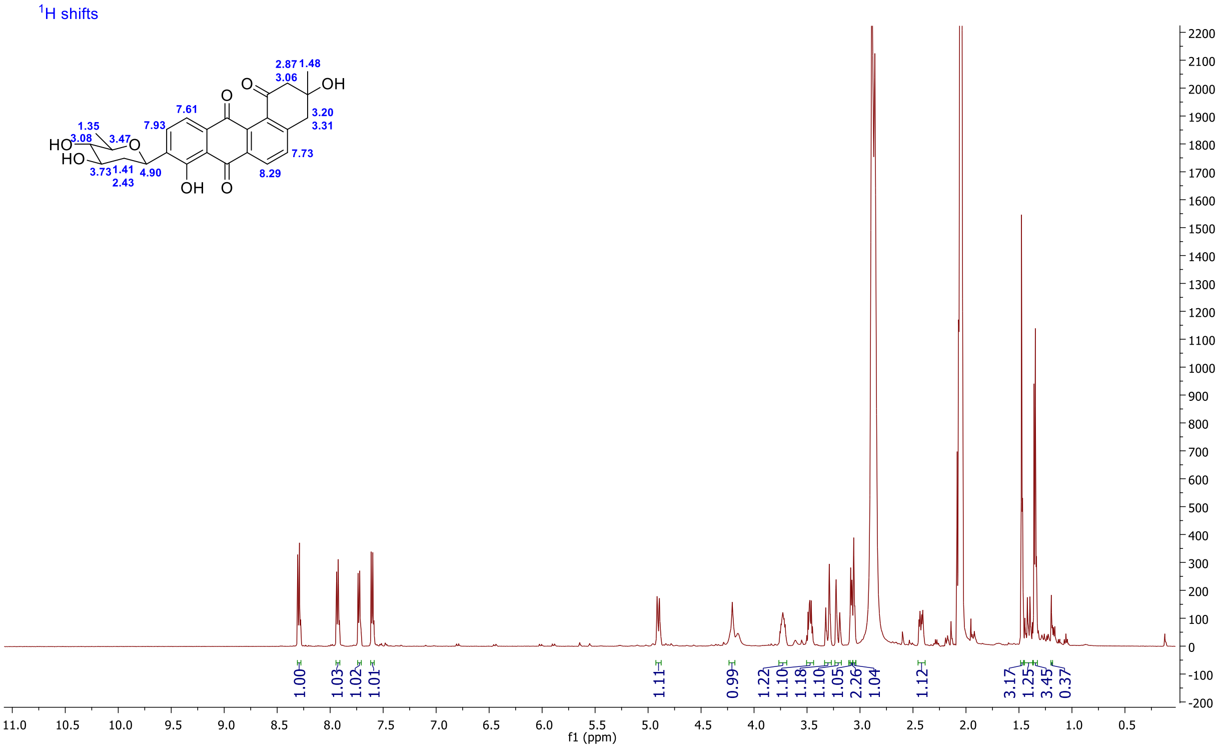

Figure S42. ^{1}H NMR (500 MHz, acetone-d_6) spectrum of urdamycinone B (**20**).

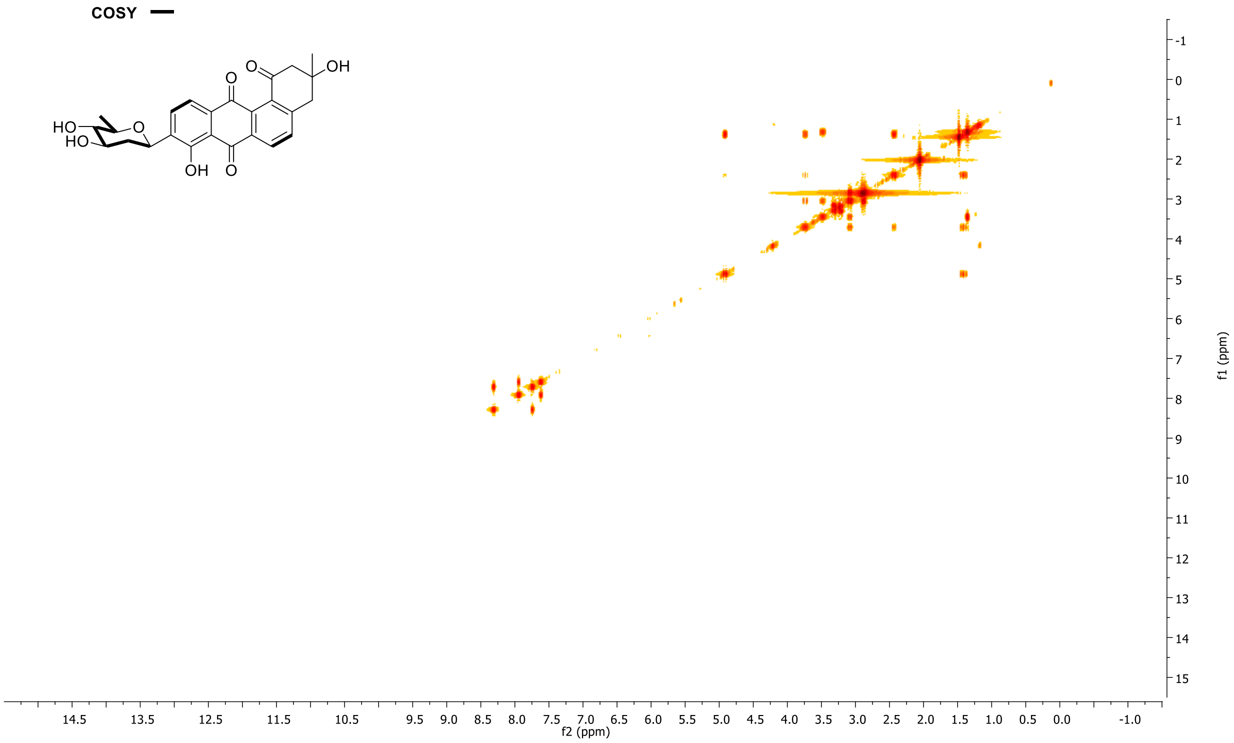

Figure S43. *g*COSY (500 MHz, acetone-d_6) spectrum of urdamycinone B (**20**).

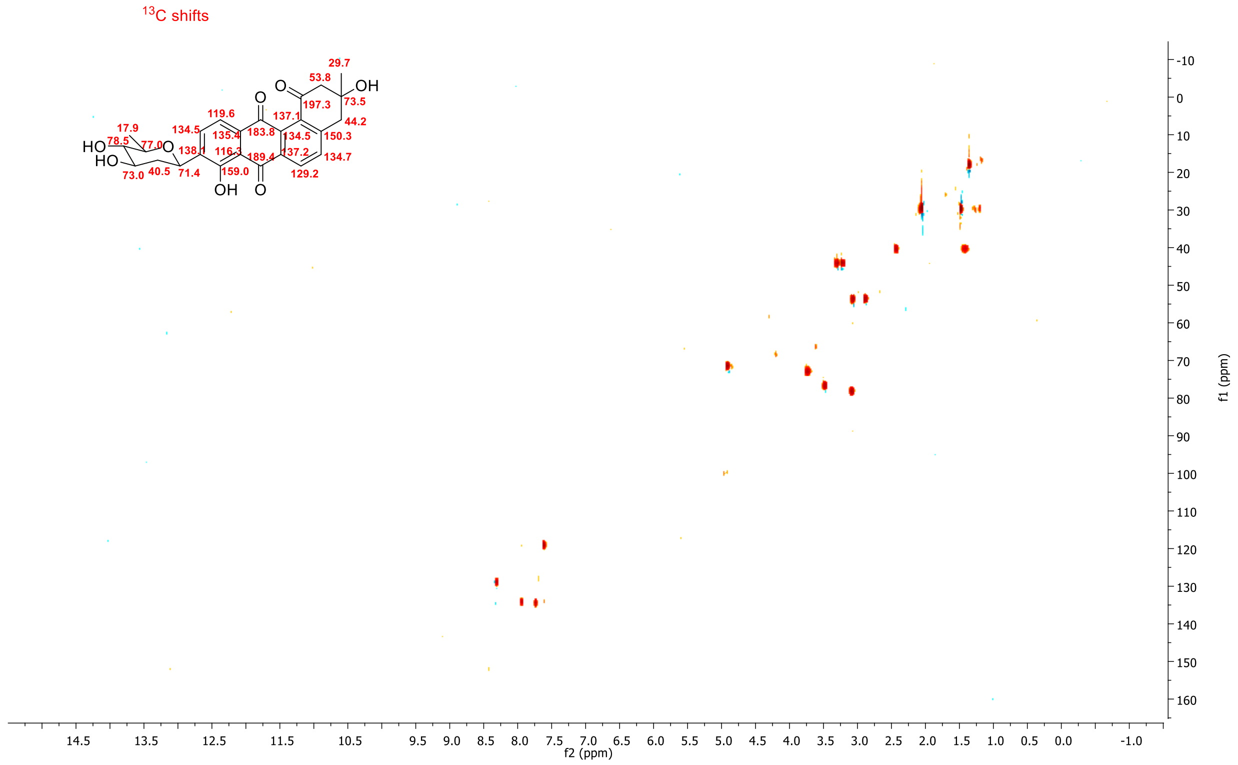

Figure S44. *g*HSQC (500 MHz, acetone-d_6) spectrum of urdamycinone B (**20**).

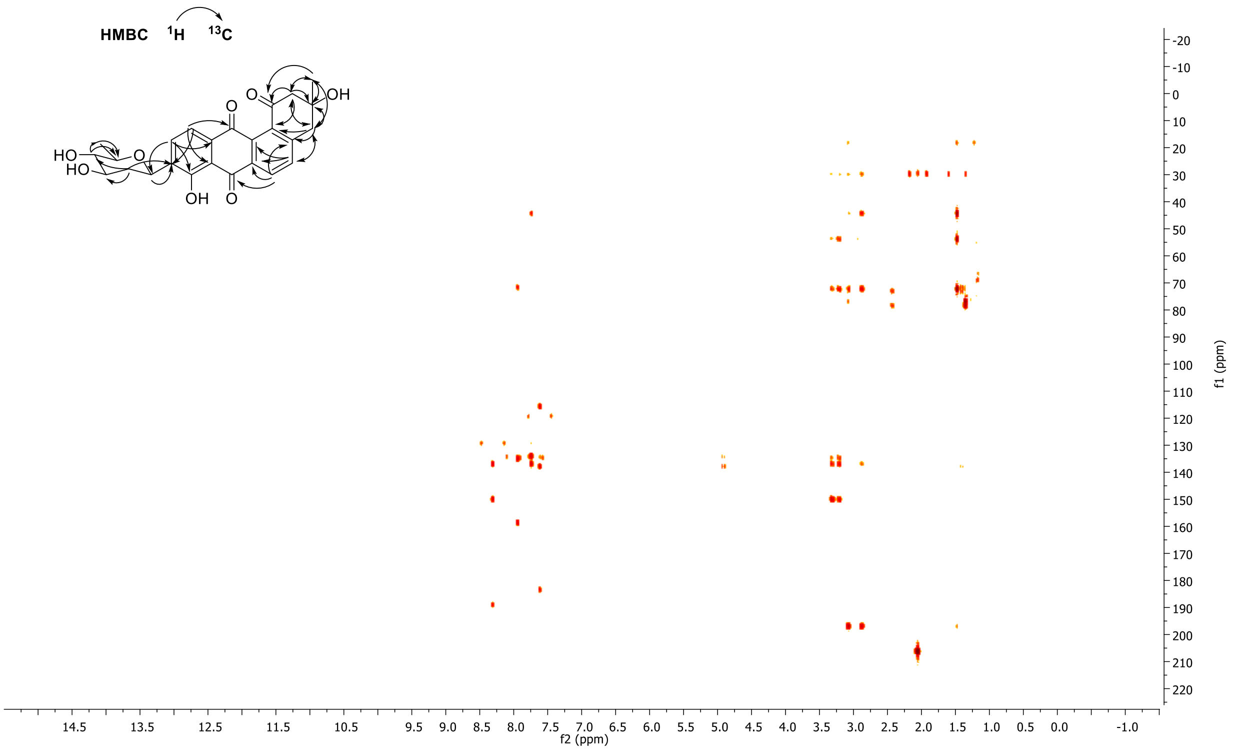

Figure S45. *g*HMBC (500 MHz, acetone-*d*$_6$) spectrum of urdamycinone B (**20**).

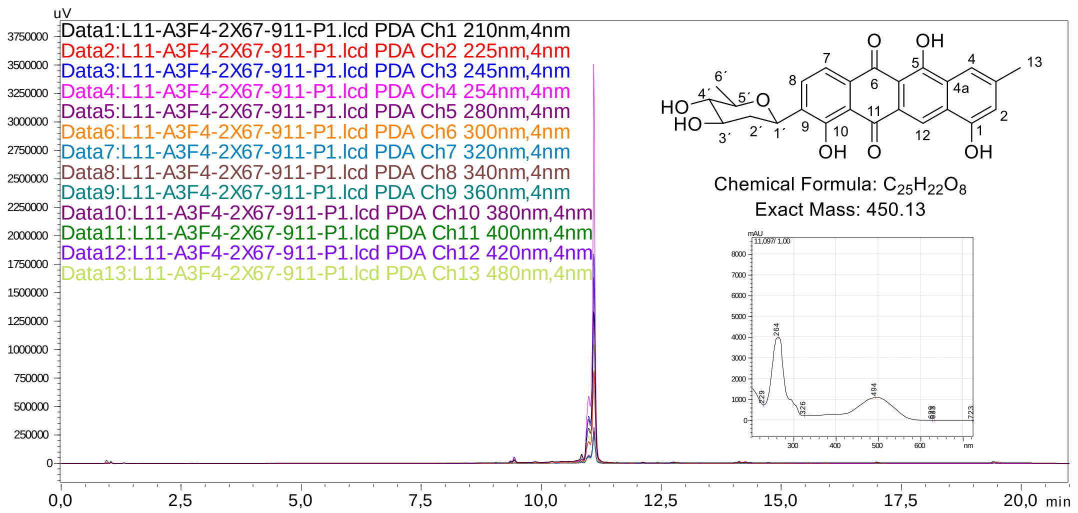

Figure S46. HPLC-DAD of purified peak corresponding to compound (**21**)

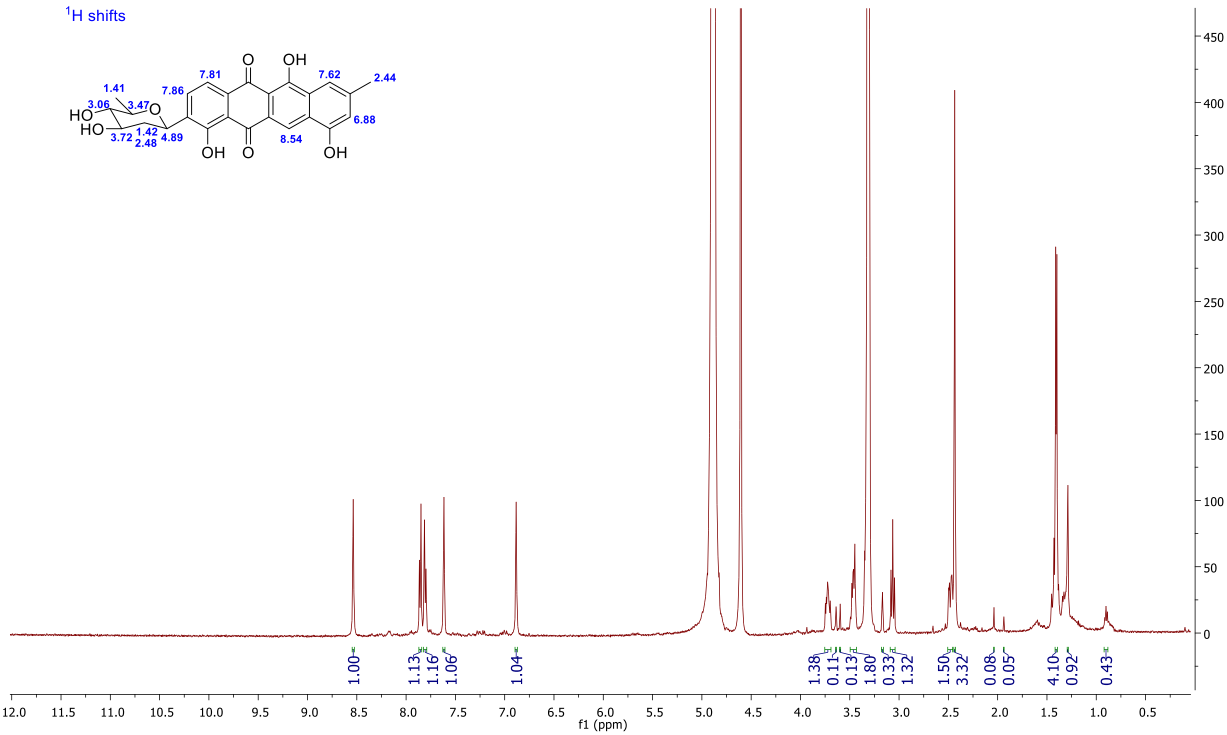

Figure S47. ^{1}H NMR (500 MHz, MeOH-d_4) spectrum of galtamycinone (**21**).

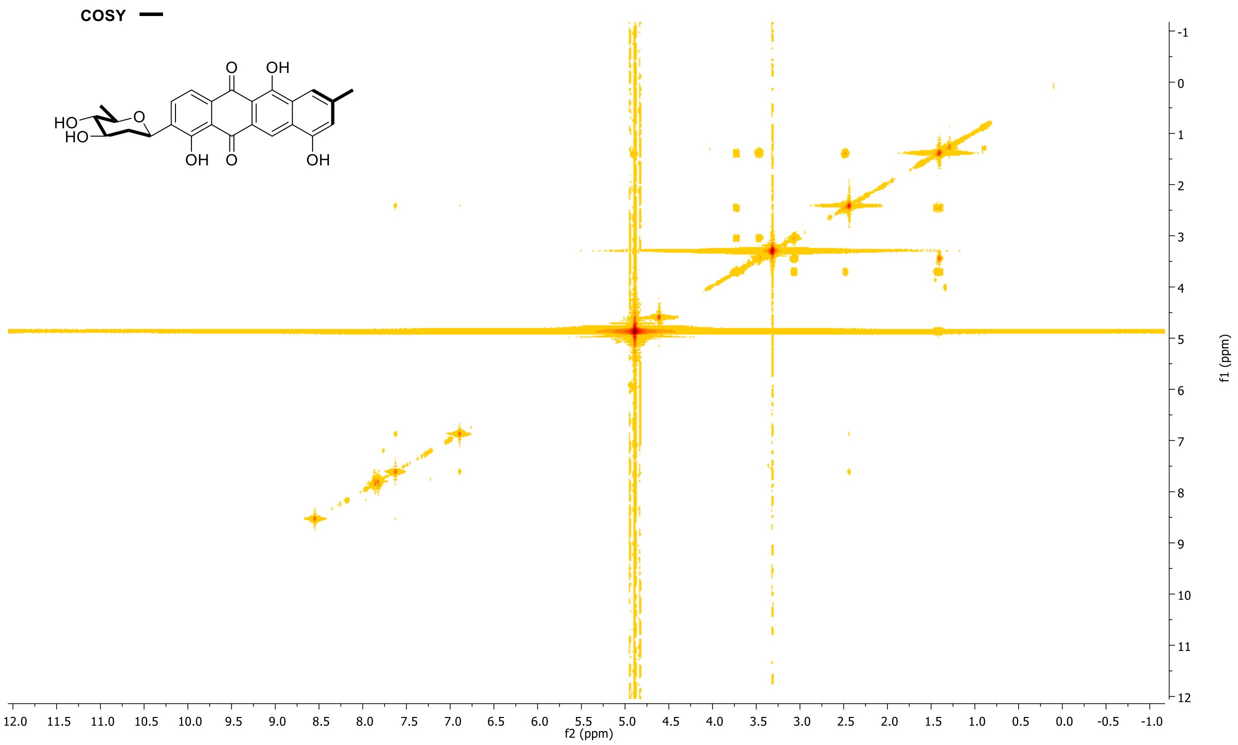

Figure S48. *g*COSY (500 MHz, MeOH-d_4) spectrum of galtamycinone (**21**).

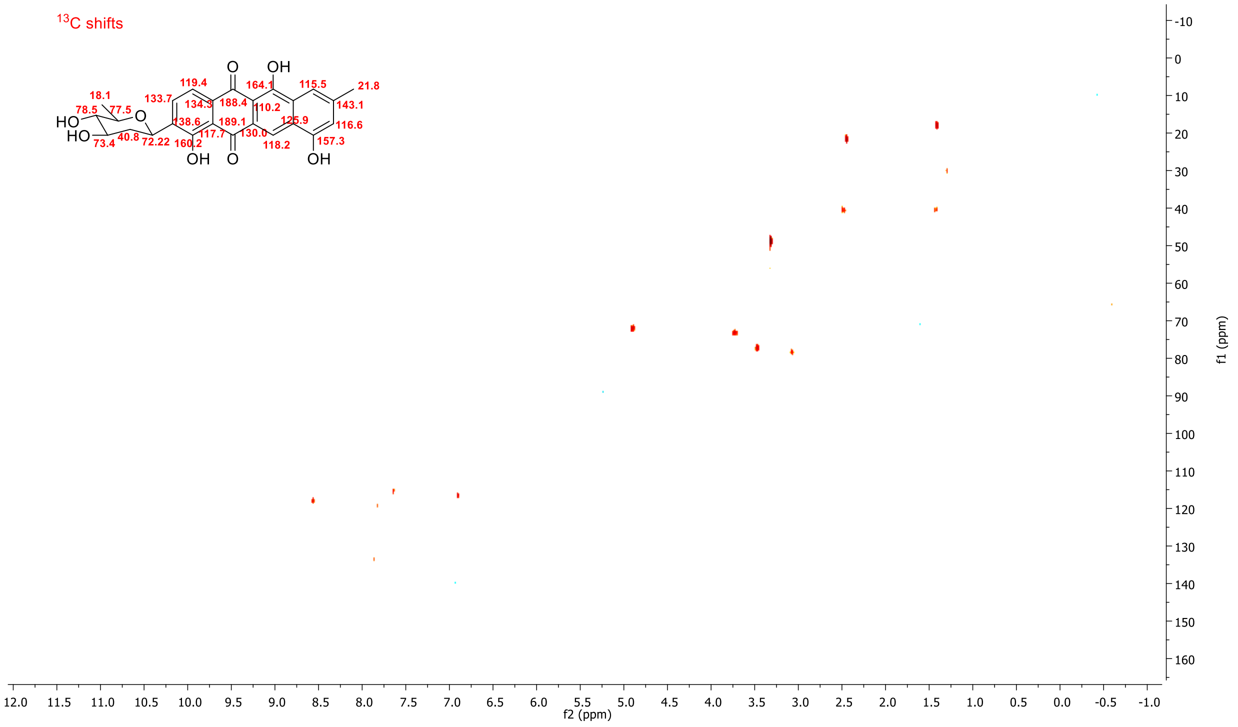

Figure S49. *g*HSQC (500 MHz, MeOH-d_4) spectrum of galtamycinone (**21**).

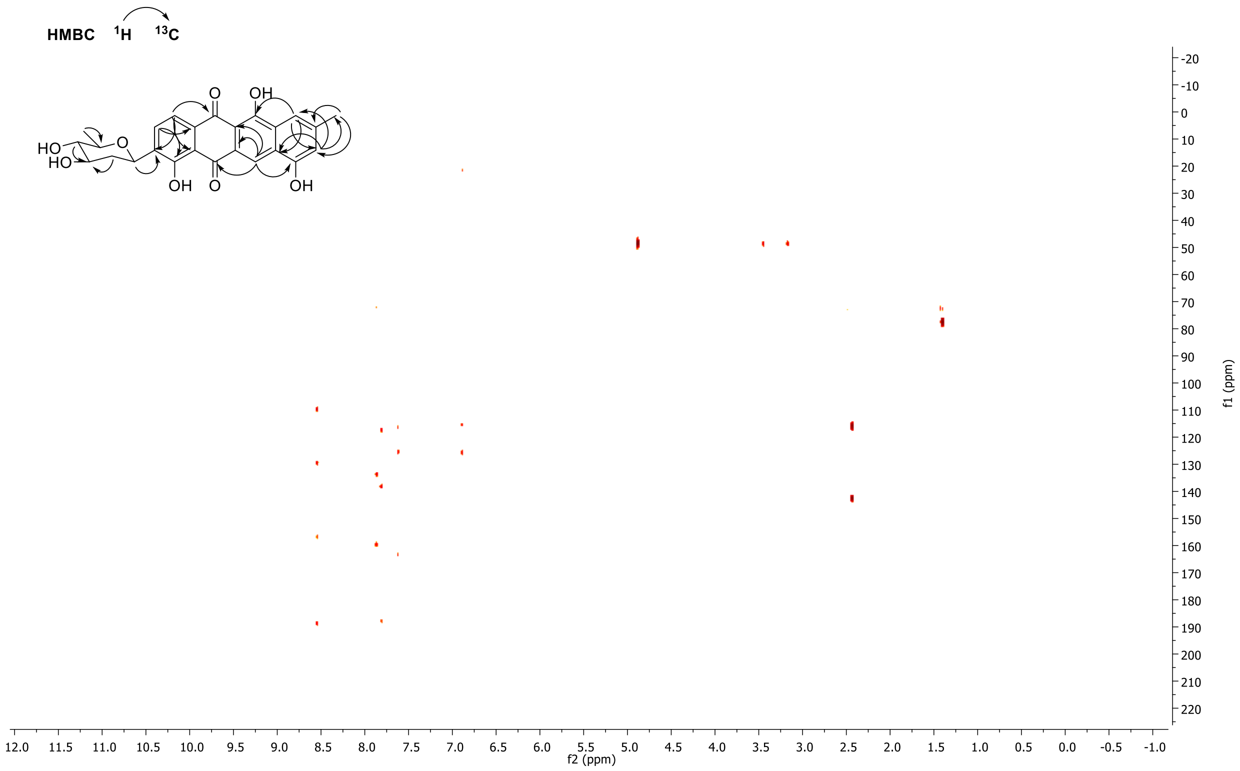

Figure S50. *g*HMBC (500 MHz, MeOH-*d*4) spectrum of galtamycinone (**21**).

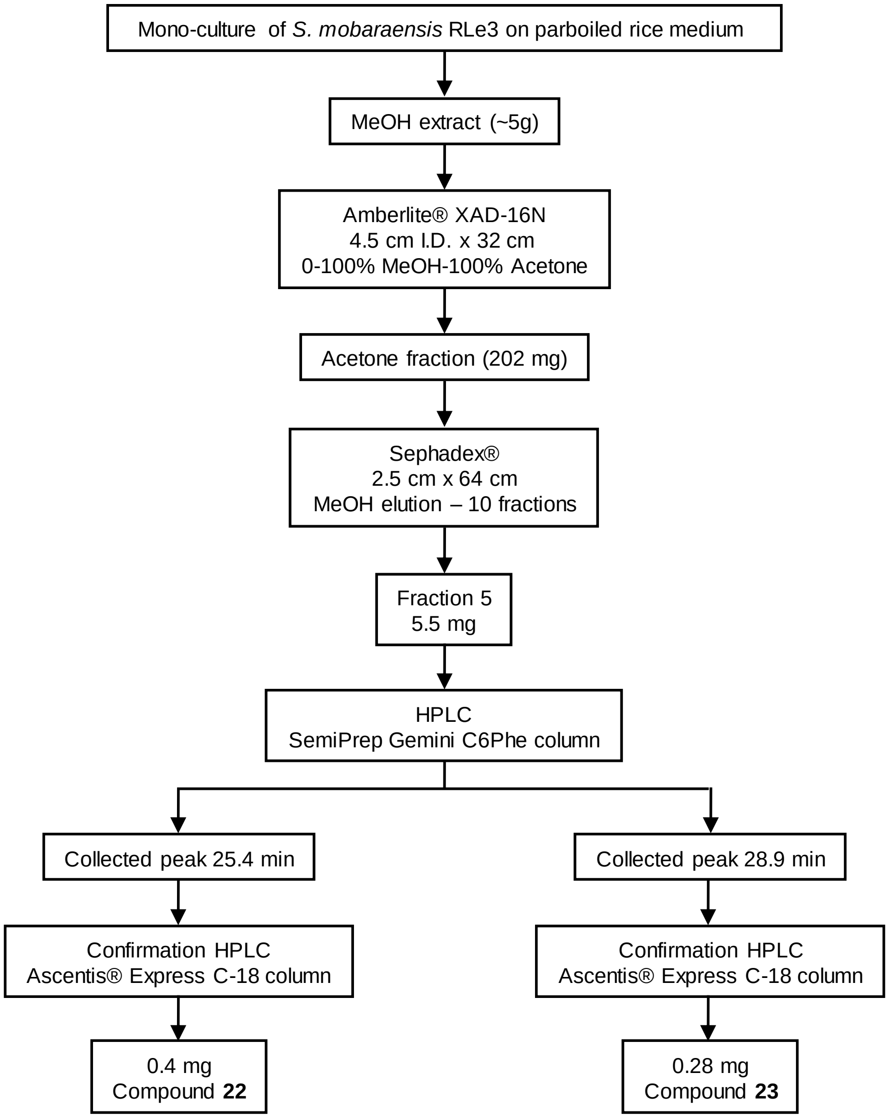

Figure S51. Purification workflow for compounds **22** and **23**

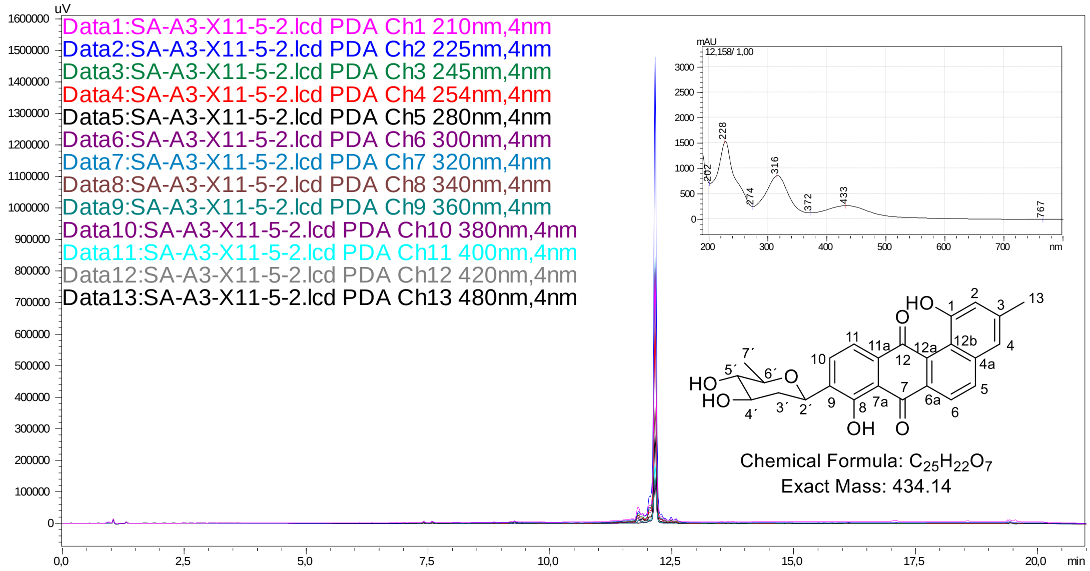

Figure S52. HPLC-DAD of purified peak corresponding to compound (**22**)

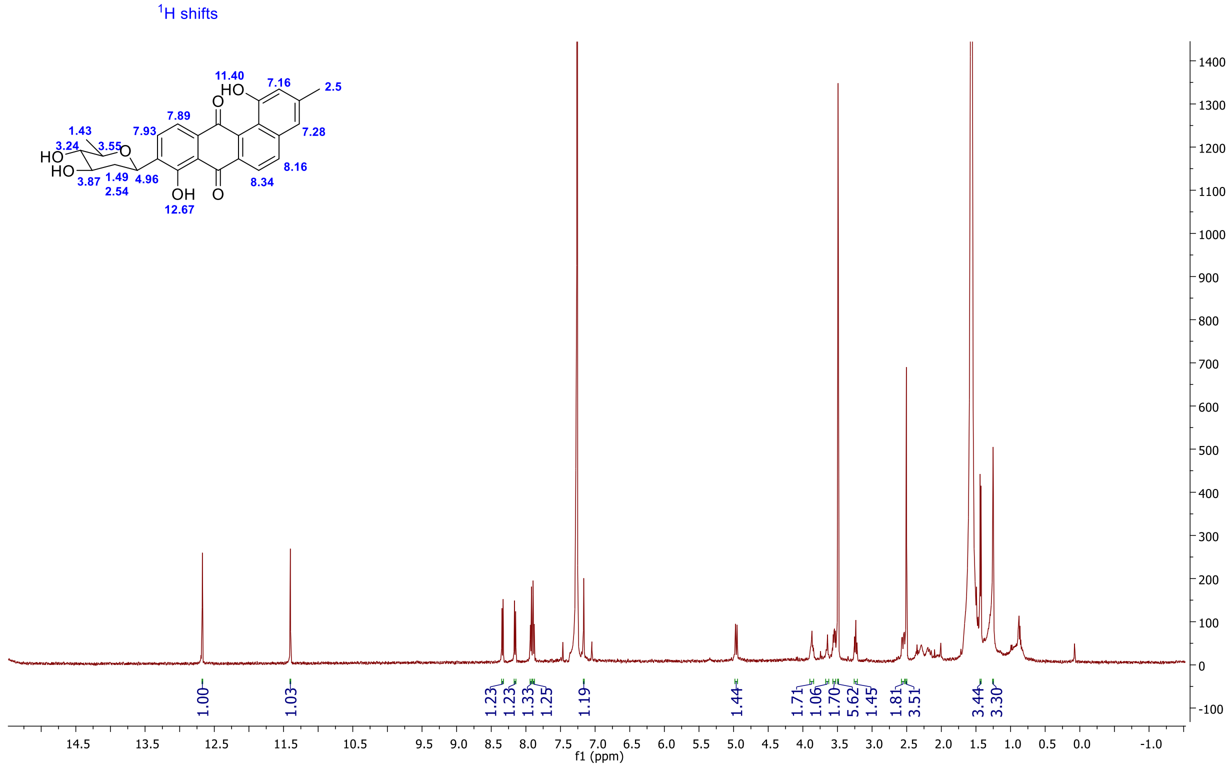

Figure S53. ^{1}H NMR (500 MHz, $CDCl_3$) spectrum of dehydroxyaquayamycin (**22**).

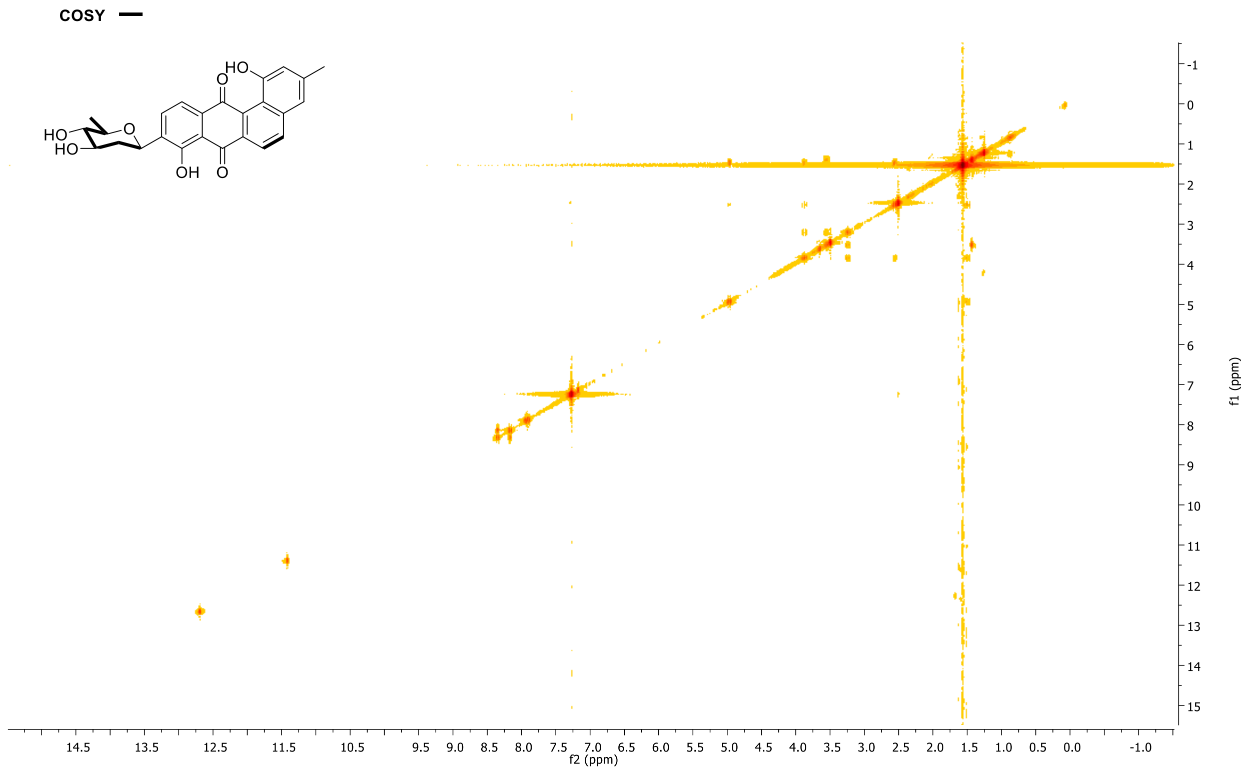

Figure S54. *g*COSY (500 MHz, $CDCl_3$) spectrum of dehydroxyaquayamycin (**22**).

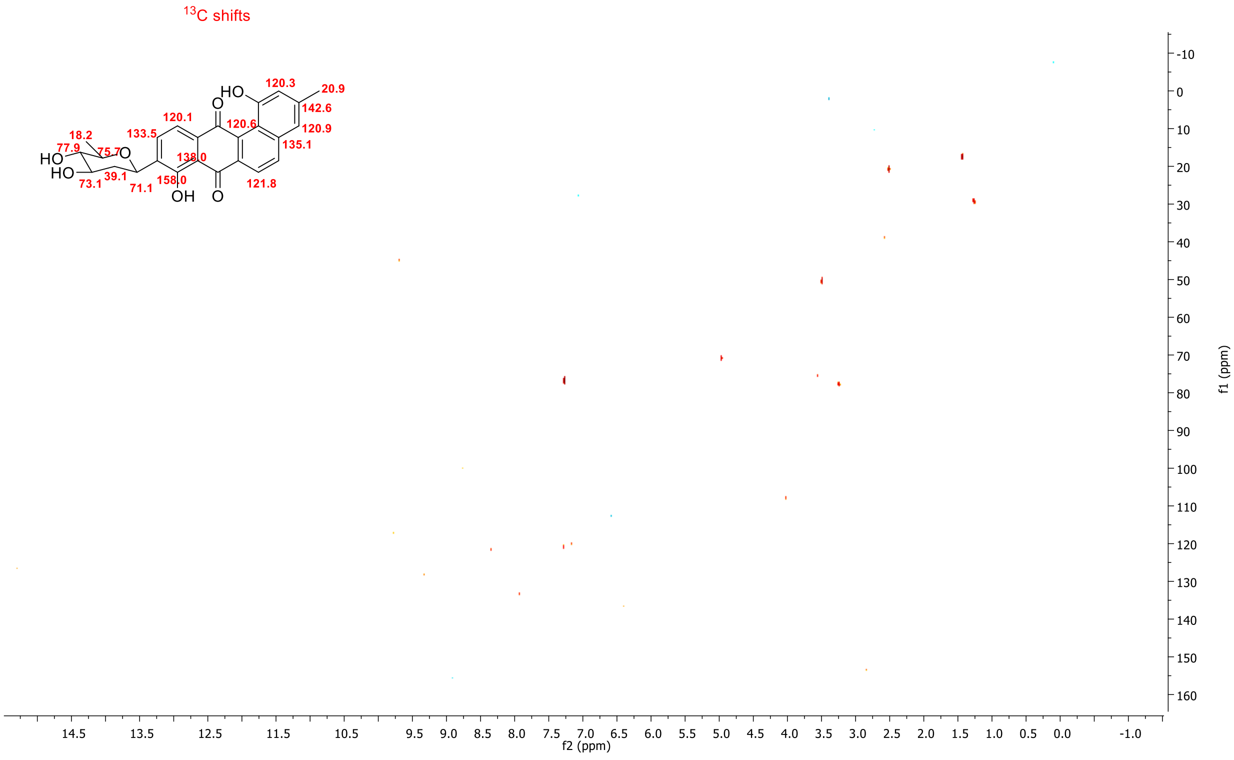

Figure S55. *g*HSQC (500 MHz, $CDCl_3$) spectrum of dehydroxyaquayamycin (**22**).

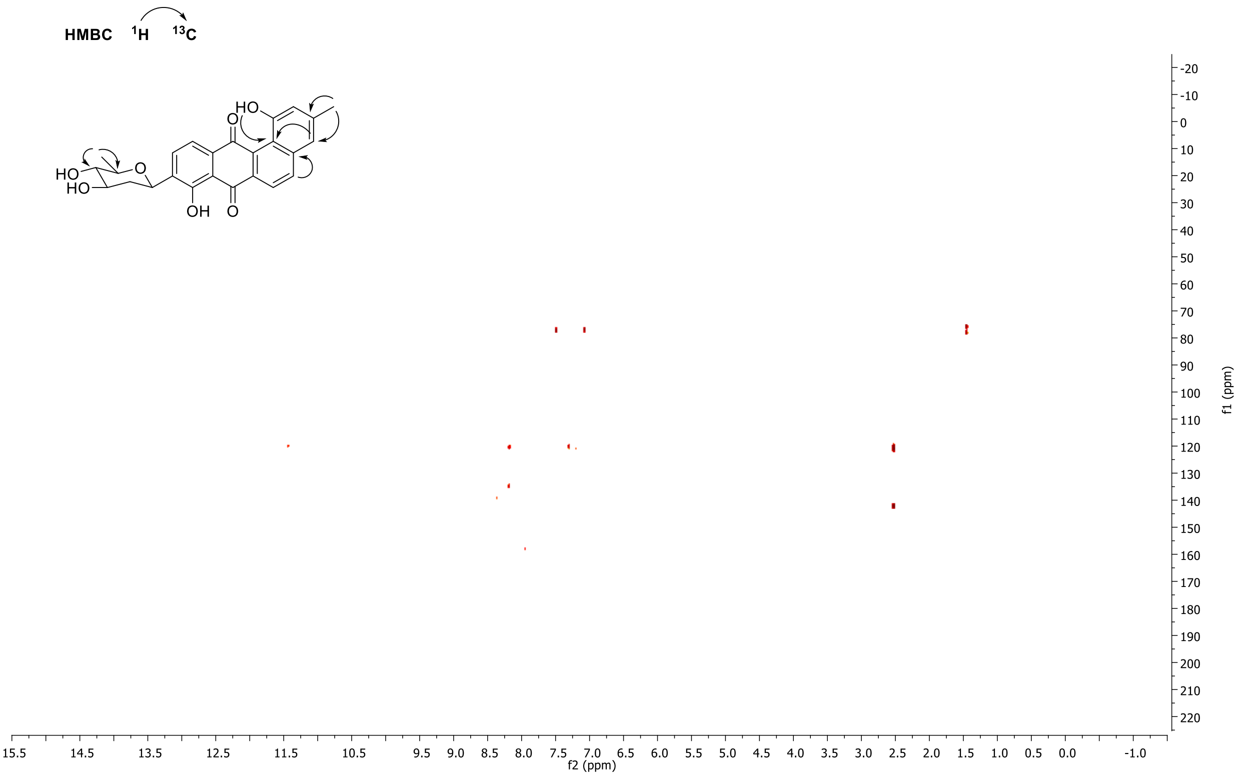

Figure S56. *g*HMBC (500 MHz, $CDCl_3$) spectrum of dehydroxyaquayamycin (**22**).

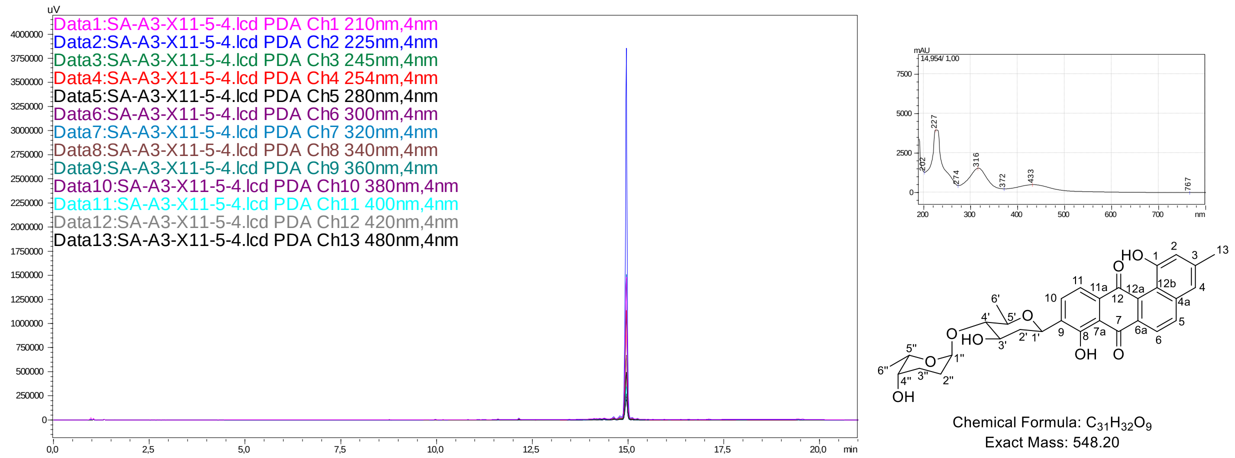

Figure S57. HPLC-DAD of purified peak corresponding to compound (**23**)

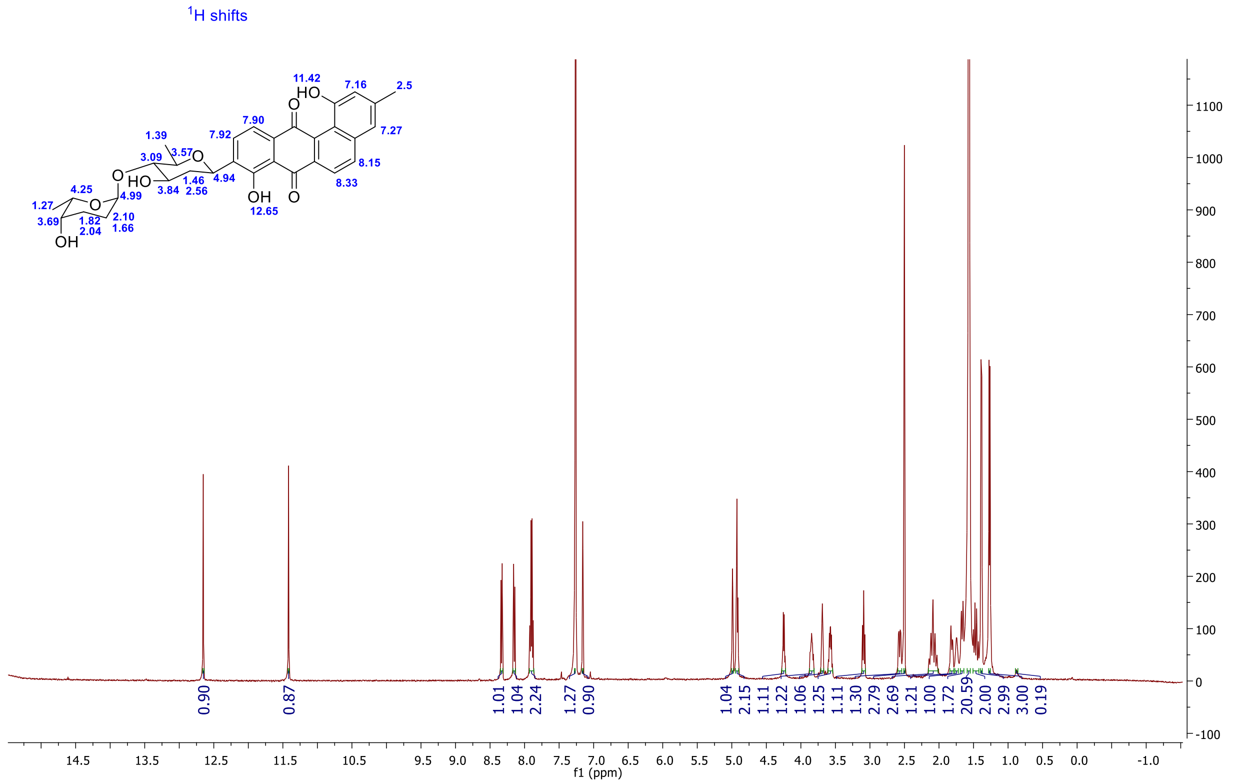

Figure S58. ^{1}H NMR (500 MHz, $CDCl_3$) spectrum of marangucycline A_2 (**23**).

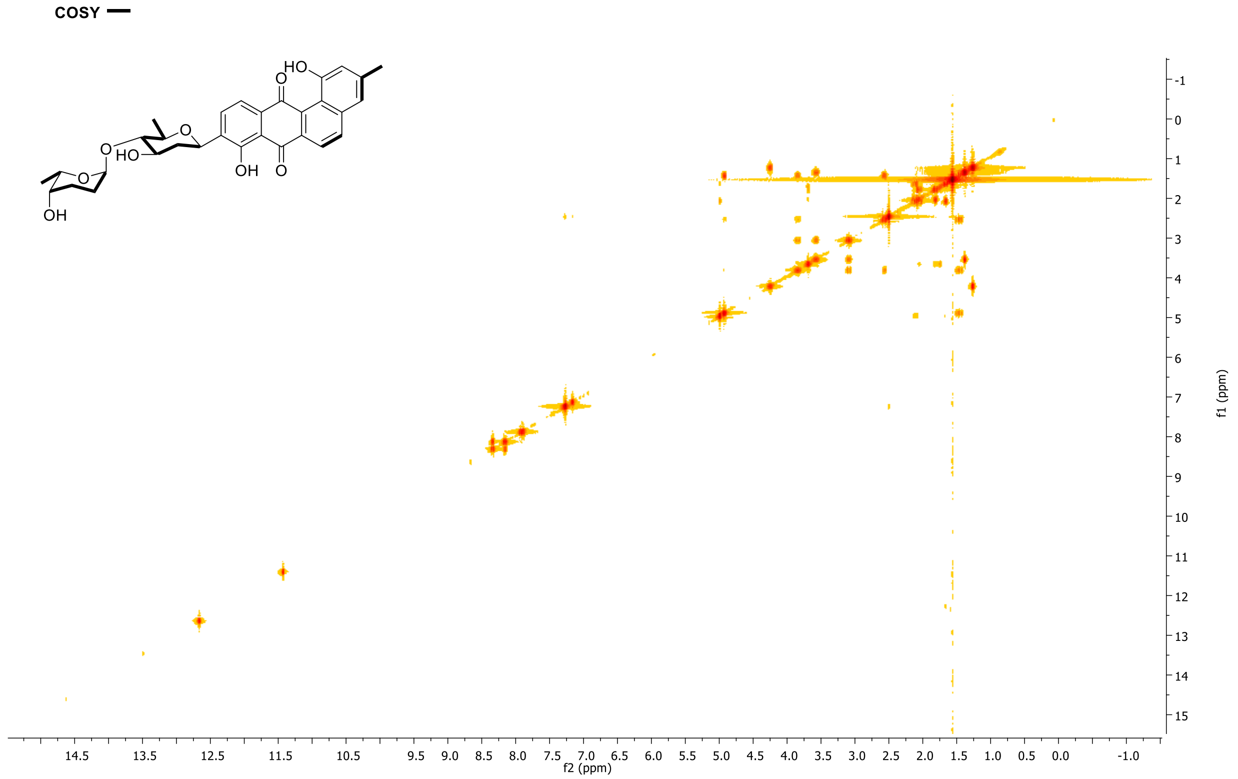

Figure S59. COSY (300 MHz, $CDCl_3$) spectrum of marangucycline A_2 (**23**).

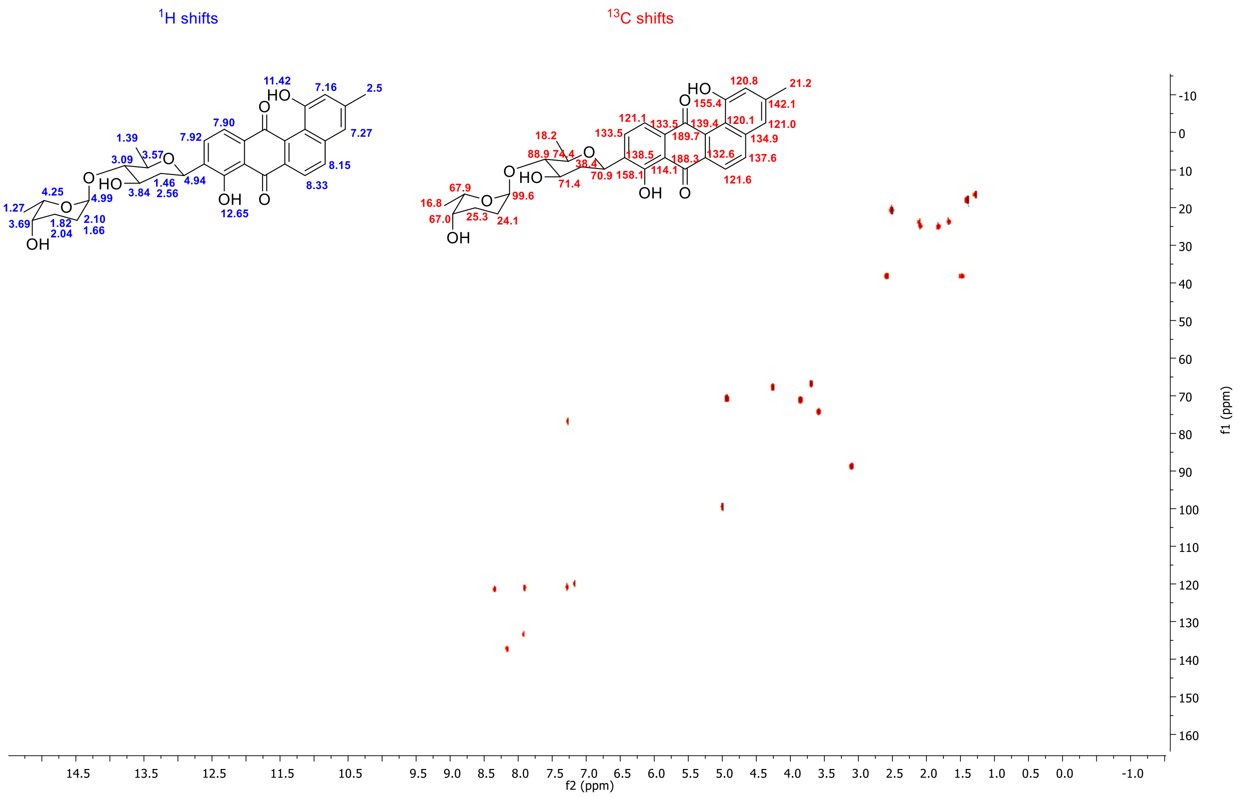

Figure S60. *g*HSQC (400 MHz, $CDCl_3$) spectrum of marangucycline A_2 (**23**).

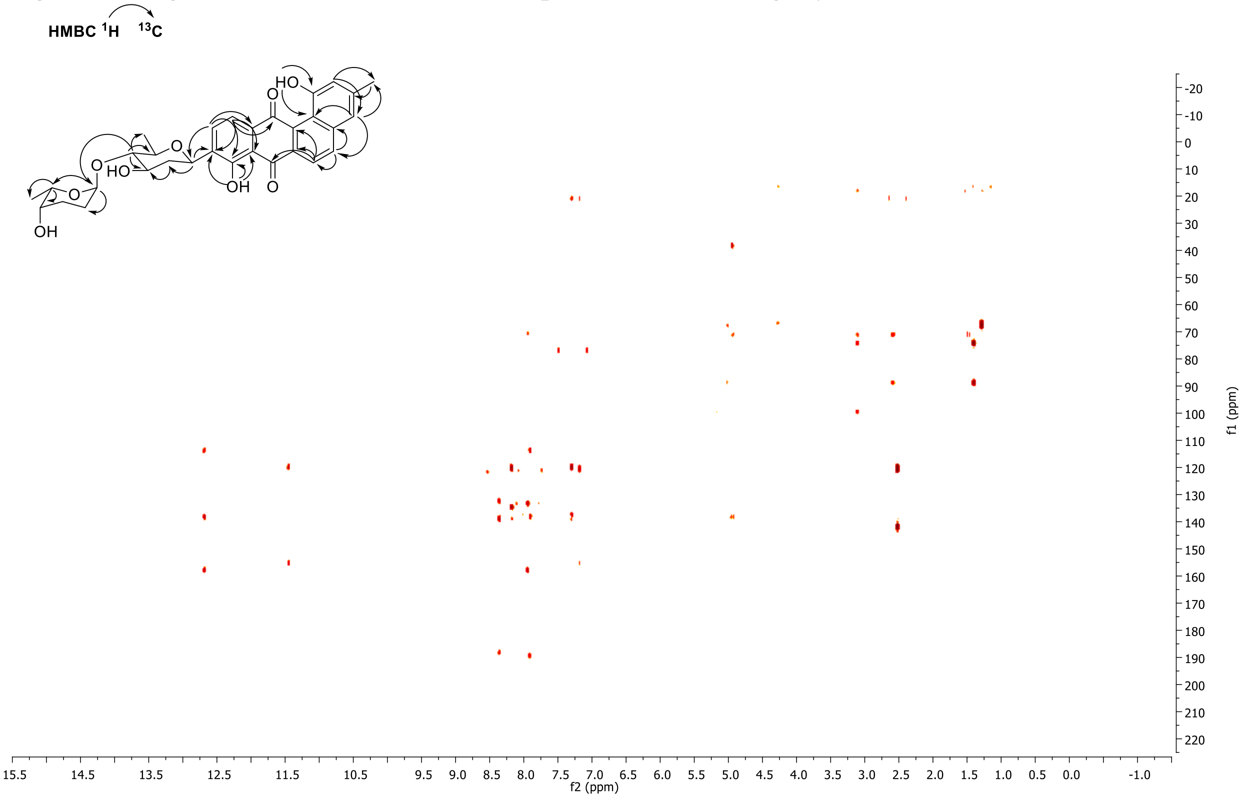

Figure S61. *g*HMBC (500 MHz, $CDCl_3$) spectrum of marangucycline A_2 (**23**).

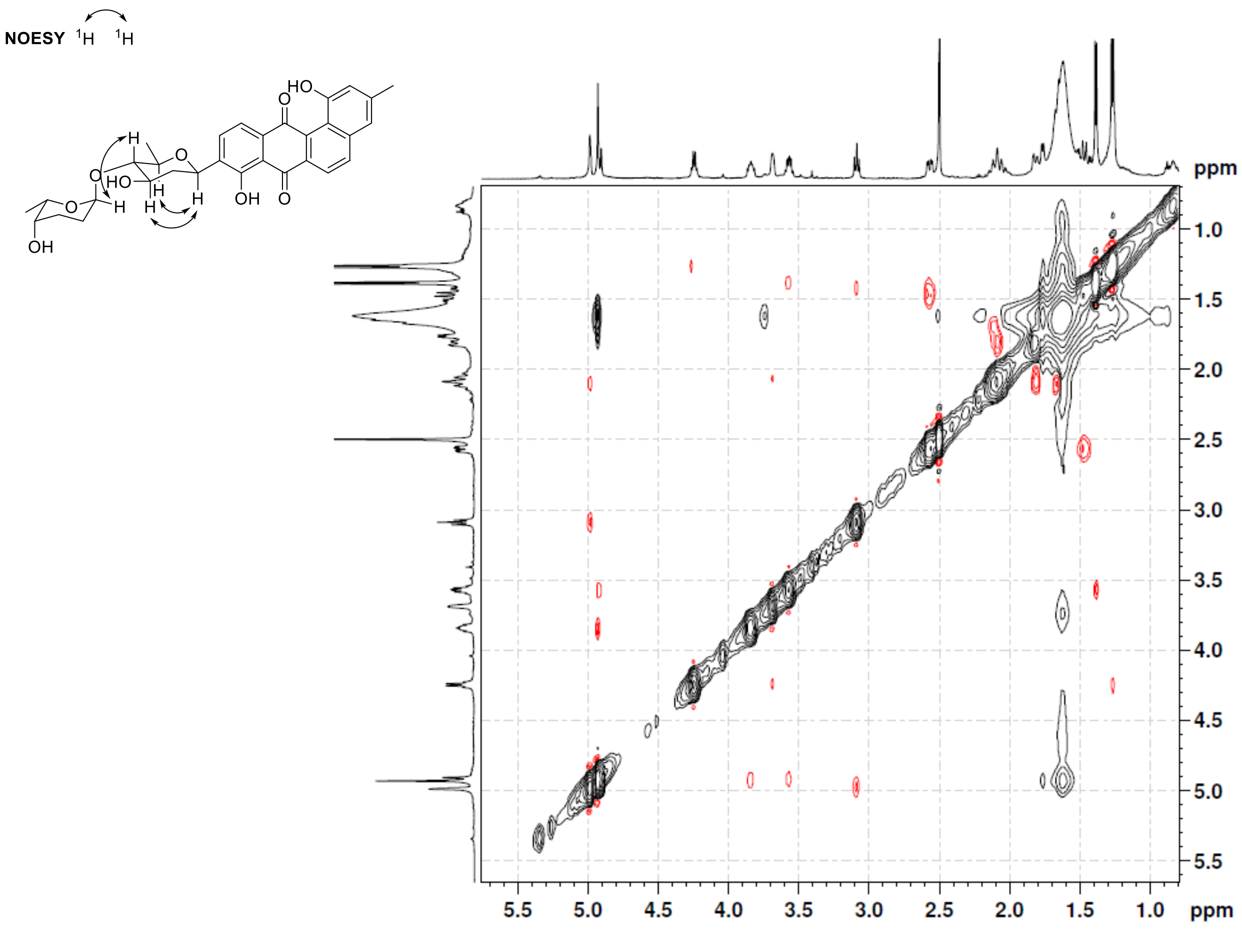

Figure S62. NOESY (500 MHz, $CDCl_3$) spectrum of marangucycline A_2 (**23**).

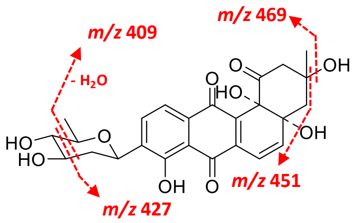

Aquayamycin (**19**)
$C_{25}H_{26}O_{10}$
$[M+H]^+$ Calcd.: 487.1599
$[M+H]^+$ Obs.: 487.1590
Δ = 1.8 ppm

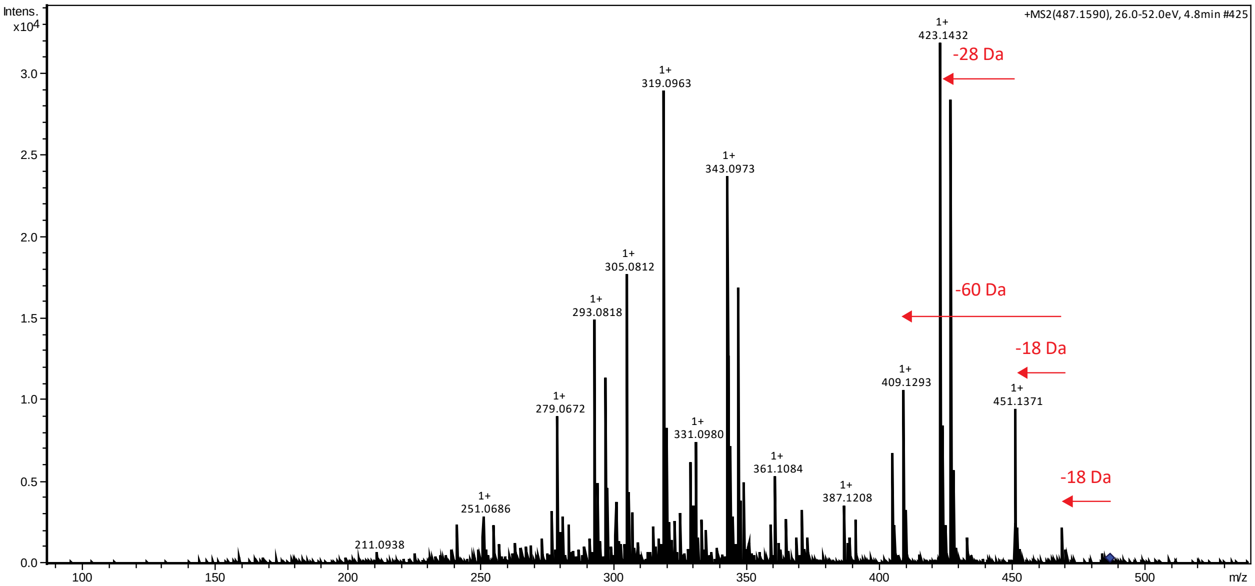

Figure S63. MS/MS spectrum of detected aquayamycin (**19**) from molecular networking of interactions amongst endophytic microorganisms from *L. ericoides*.

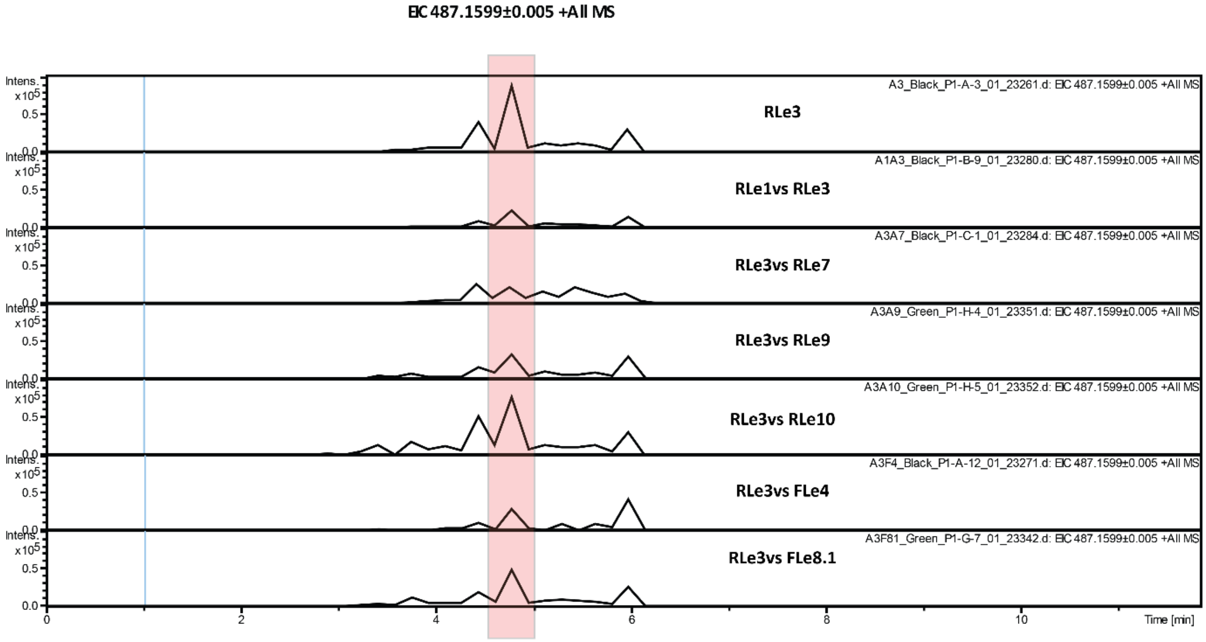

Figure S64. Extracted ion chromatogram comparison for compound (**19**)
Aquayamycin (**19**), *m/z* 487, was produced by *S. mobaraensis* RLe3 and detected in mono- and co-cultures. The EIC are representative from four replicates.

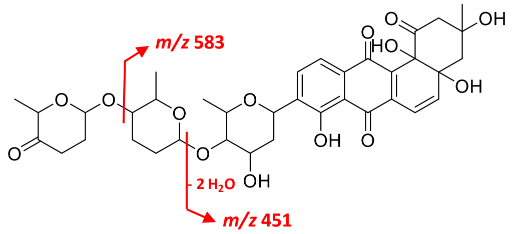

Antibiotic DQ 112A as representative annotation

Aquayamycin analogue (**24**)
$C_{37}H_{44}O_{14}$
$[M+H]^+$ Calcd.: 713.2804
$[M+H]^+$ Obs.: 713.2843
Δ = - 5.5 ppm

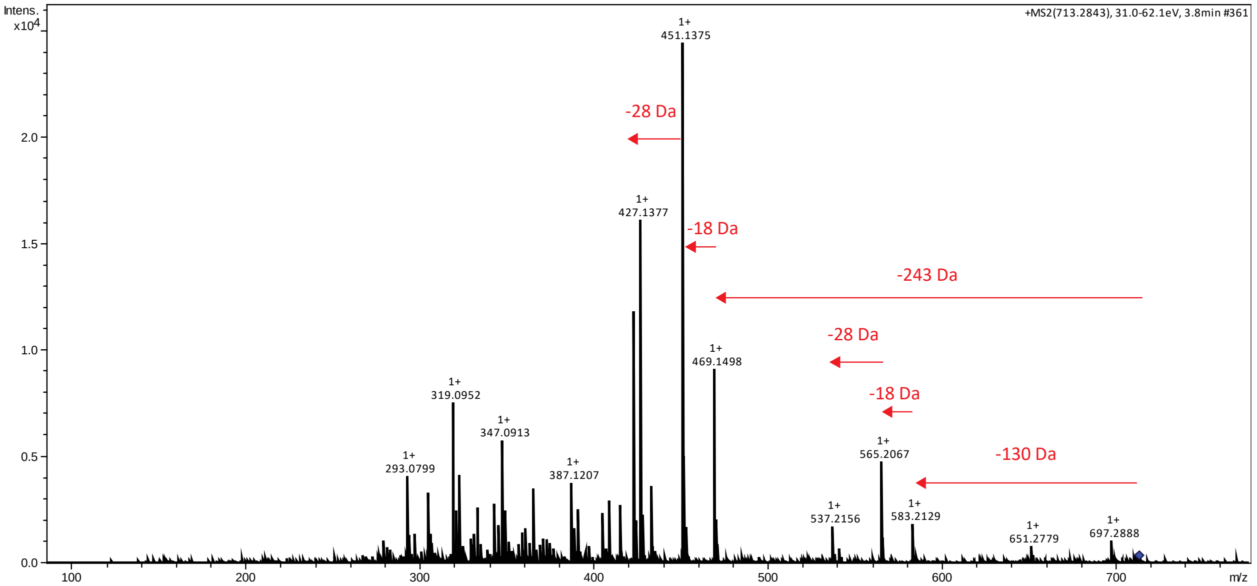

Figure S65. MS/MS spectrum of detected aquayamycin analogue *m/z* 713 (**24**) from the molecular network of interactions amongst endophytic microorganisms from *L. ericoides*.

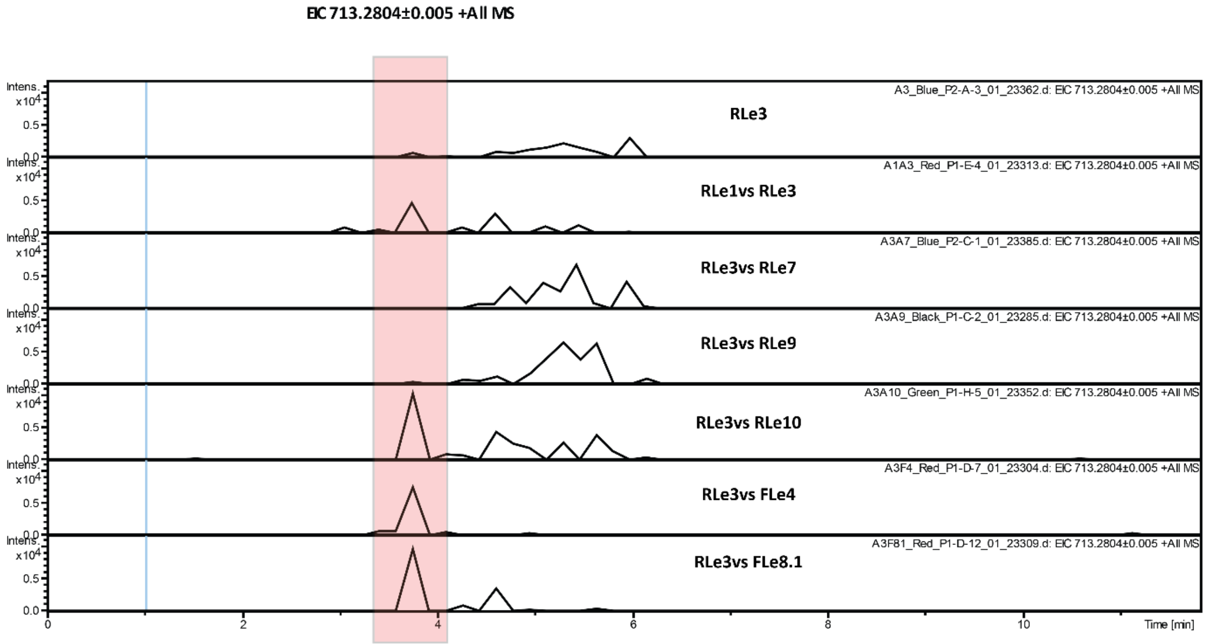

Figure S66. Extracted ion chromatogram comparison for compound (**24**)
Aquayamycin analogue (**19**), *m/z* 713, was produced by *S. mobaraensis* RLe3 and detected in mono- and co-cultures. The EIC are representative from four replicates.

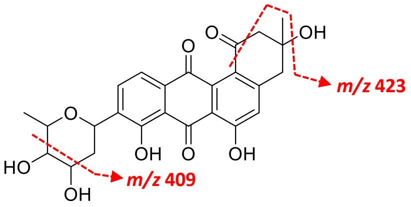

Aquayamycin analogue (**25**)
$C_{25}H_{24}O_9$
$[M+H]^+$ Calcd.: 469.1493
$[M+H]^+$ Obs.: 469.1493
Δ = 0.0 ppm

Antibiotic BA 12100MY1 as representative annotation

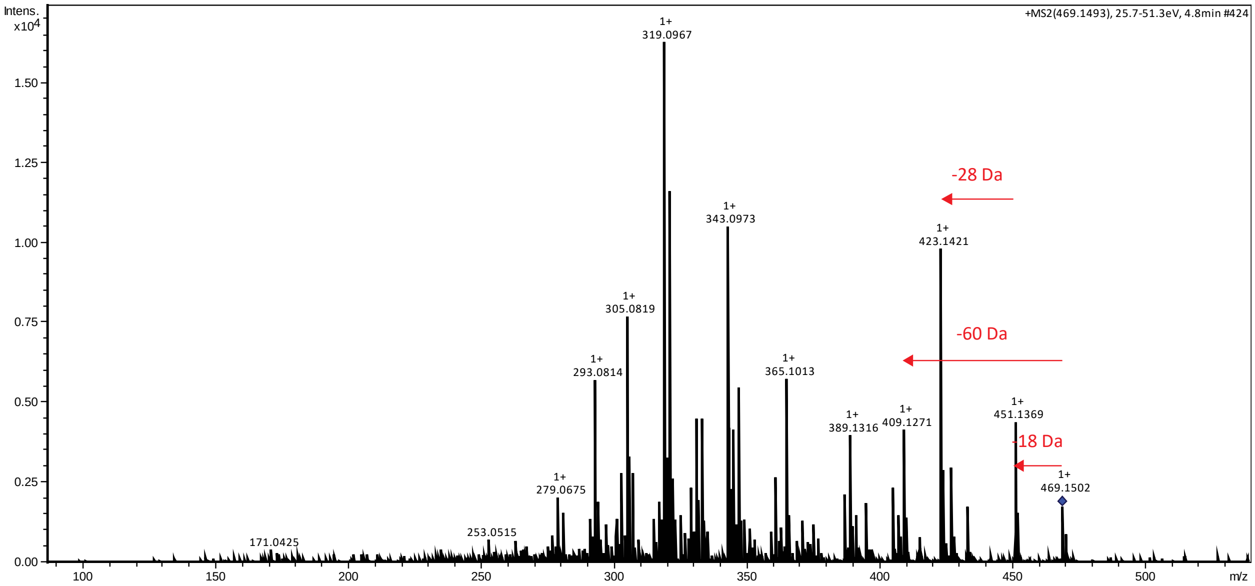

Figure S67. MS/MS spectrum of detected aquayamycin analogue *m/z* 469 (**25**) from the molecular network of interactions amongst endophytic microorganisms from *L. ericoides*.

EIC 469.1493±0.005 +All MS

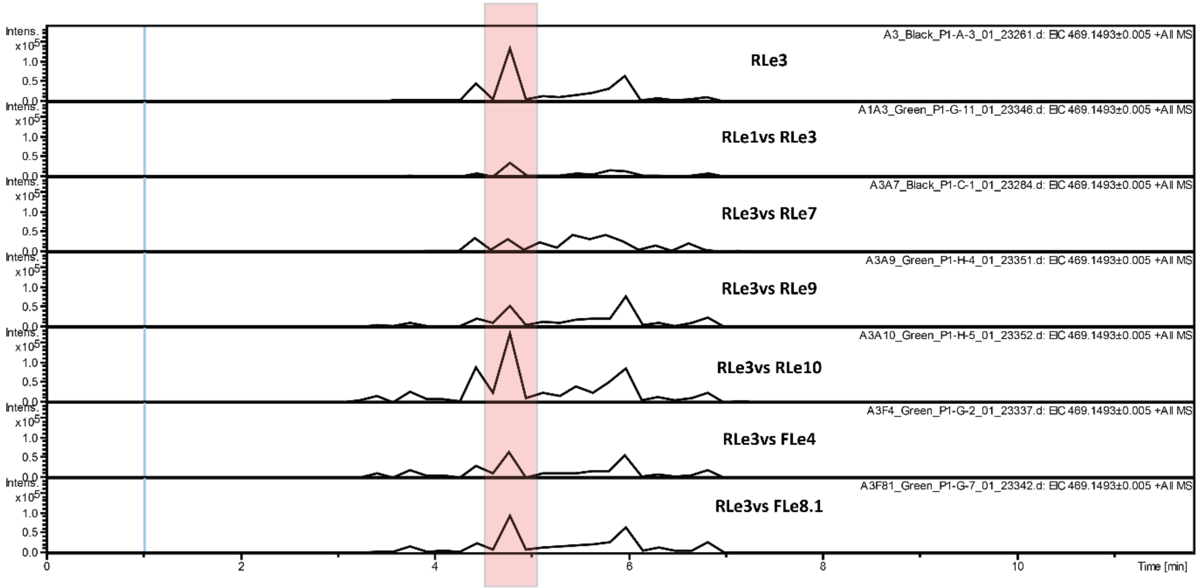

Figure S68. Extracted ion chromatogram comparison for compound (**25**)

Aquayamycin analogue (**25**), *m/z* 469, was produced by *S. mobaraensis* RLe3 and detected in mono- and co-cultures. The EIC are representative from four replicates.

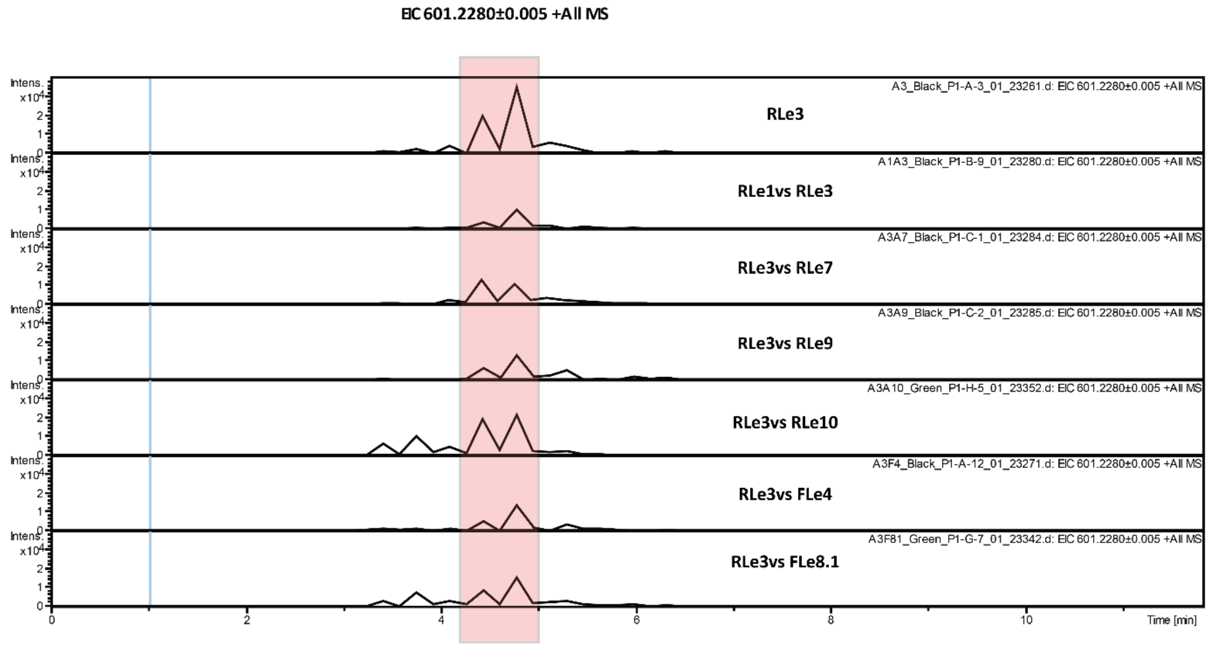

Figure S69. Extracted ion chromatogram comparison for compound (**26**)
Aquayamycin analogue (**26**), *m/z* 601, was produced by *S. mobaraensis* RLe3 and detected in mono- and co-cultures. The EIC are representative from four replicates.

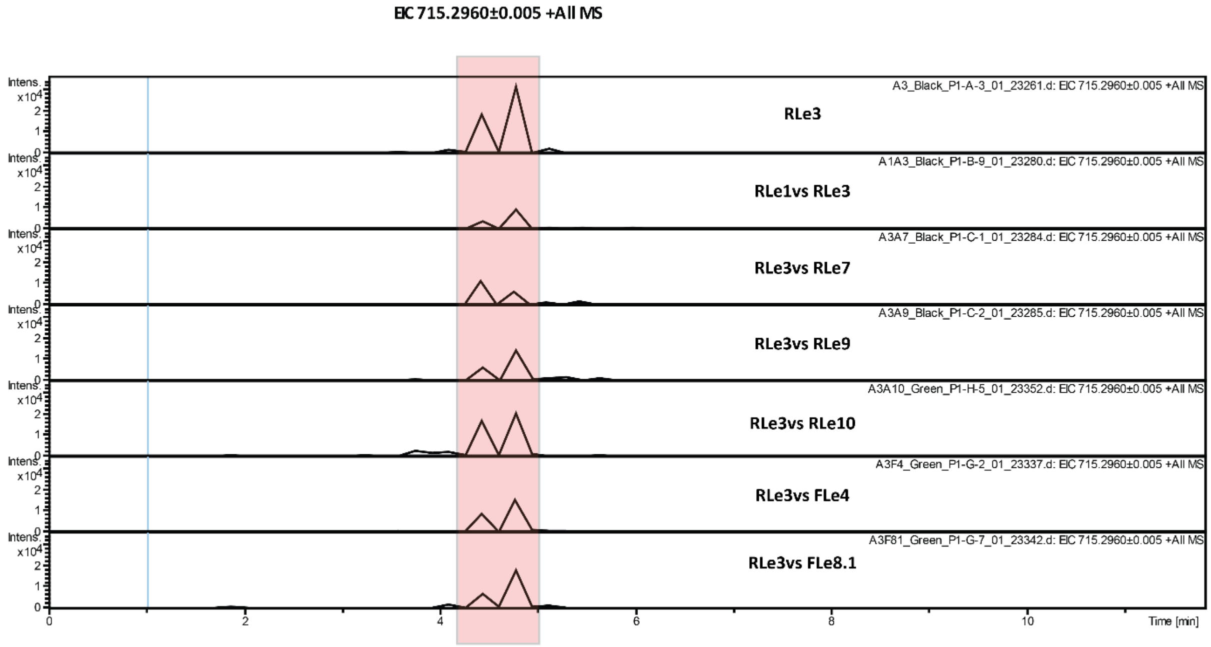

Figure S70. Extracted ion chromatogram comparison for compound (**27**)
Aquayamycin analogue (**27**), *m/z* 715, was produced by *S. mobaraensis* RLe3 and detected in mono- and co-cultures. The EIC are representative from four replicates.

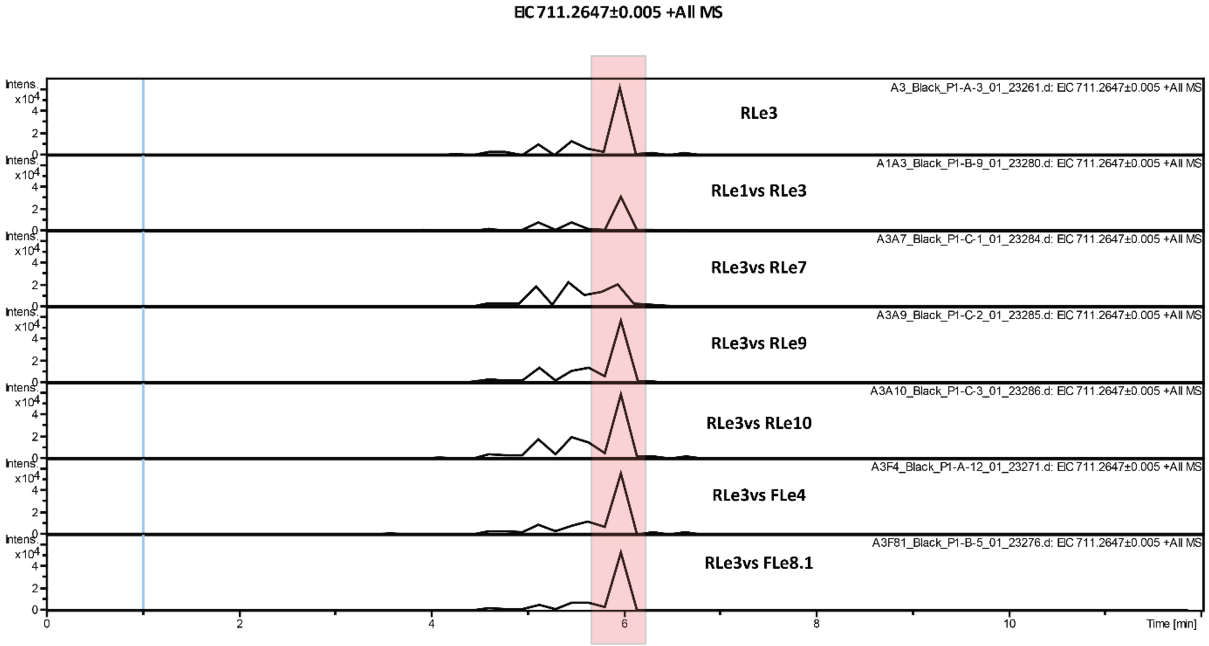

Figure S71. Extracted ion chromatogram comparison for compound (**28**)
Aquayamycin analogue (**28**), *m/z* 711, was produced by *S. mobaraensis* RLe3 and detected in mono- and co-cultures. The EIC are representative from four replicates.

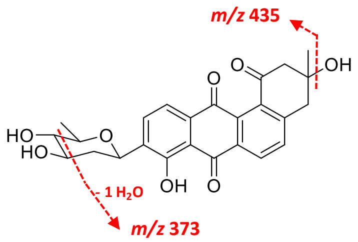

Urdamycinone B (**20**)
$C_{25}H_{24}O_8$
$[M+H]^+$ Calcd.: 453.1544
$[M+H]^+$ Obs.: 453.1548
Δ = - 0.9 ppm

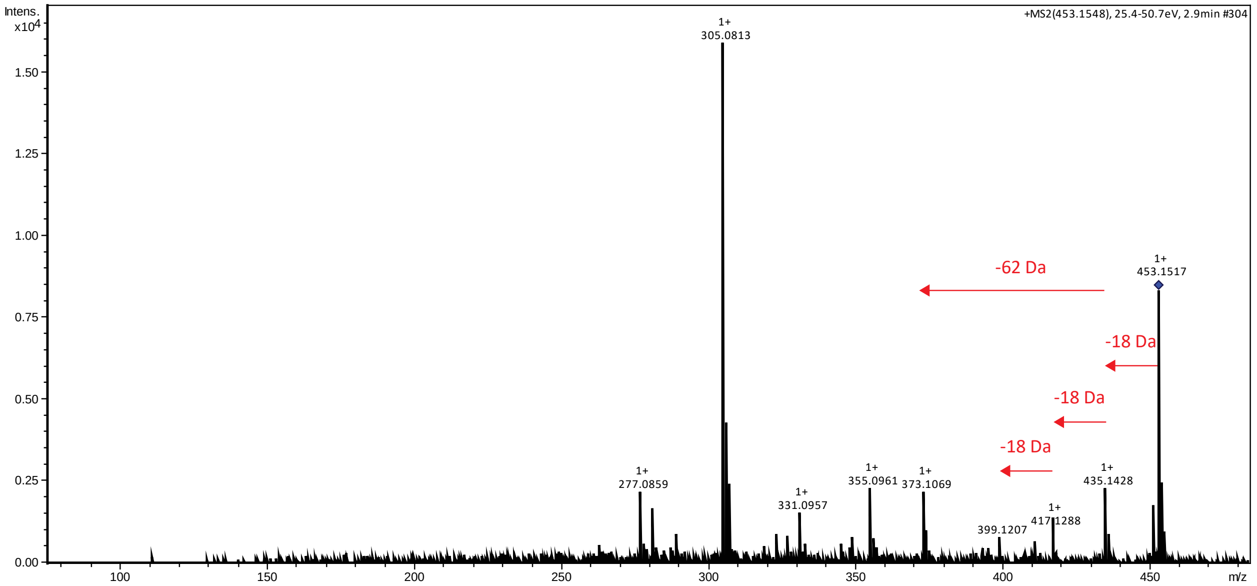

Figure S72. MS/MS spectrum of detected urdamycinone B (**20**) from the molecular network of interactions amongst endophytic microorganisms from *L. ericoides.*

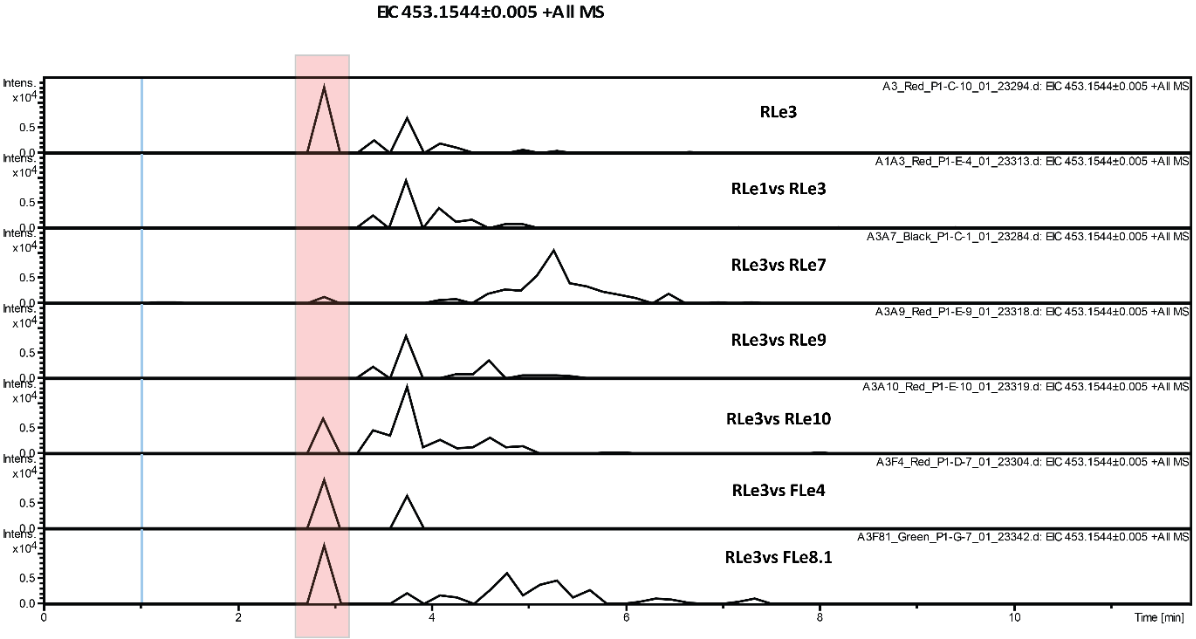

Figure S73. Extracted ion chromatogram comparison for compound (**20**)
Urdamycinone B (**28**), *m/z* 453, was produced by *S. mobaraensis* RLe3 and detected in mono- and co-cultures. The EIC are representative from four replicates.

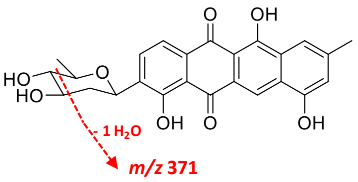

Galtamycinone (**21**)
$C_{25}H_{22}O_8$
$[M+H]^+$ Calcd.: 451.1387
$[M+H]^+$ Obs.: 451.1381
Δ = 1.4 ppm

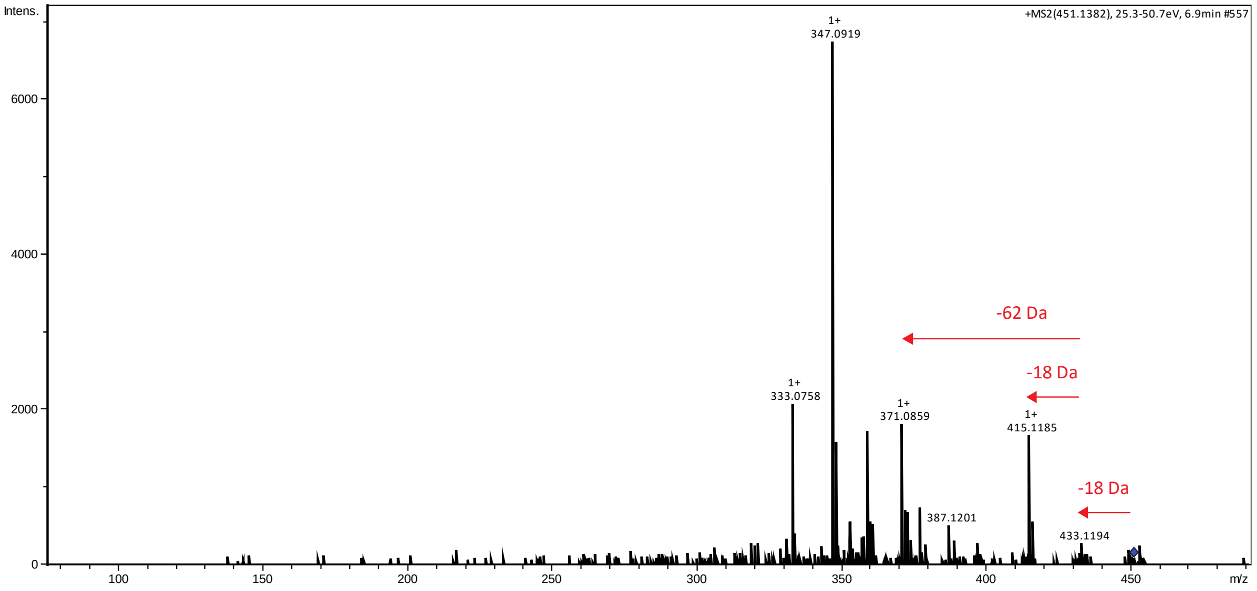

Figure S74. MS/MS spectrum of detected galtamycinone (**21**) from the molecular network of interactions amongst endophytic microorganisms from *L. ericoides*.

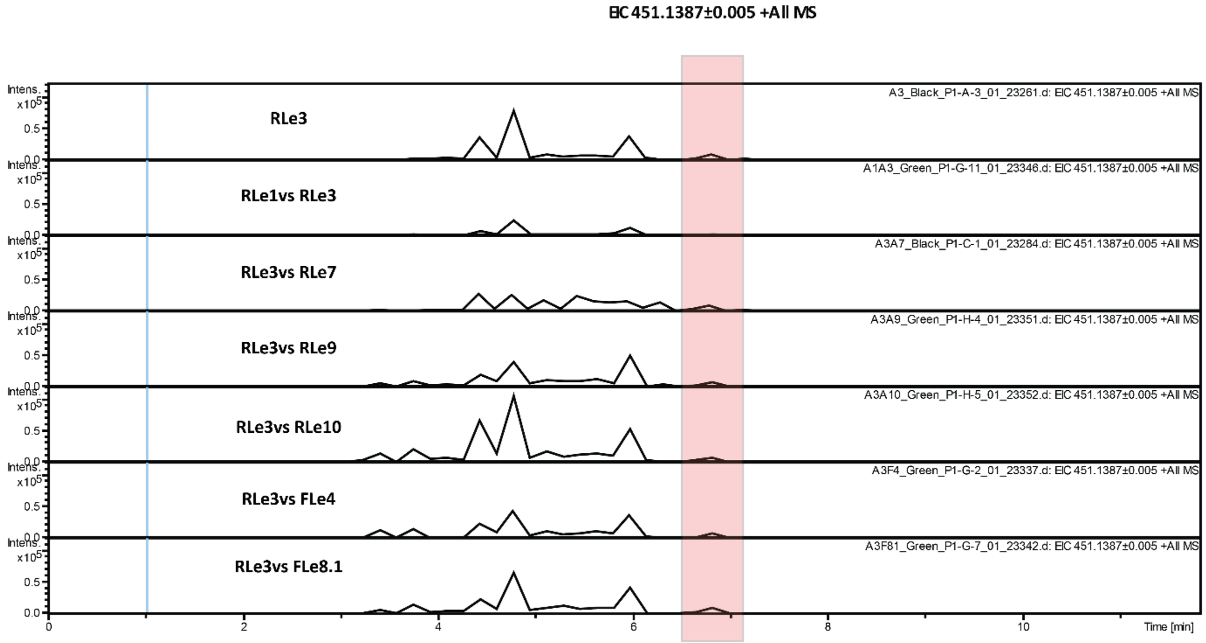

Figure S75. Extracted ion chromatogram comparison for compound (**21**)
Galtamycinone (**21**), *m/z* 451, was produced by *S. mobaraensis* RLe3 and detected in mono- and co-cultures. The EIC are representative from four replicates.

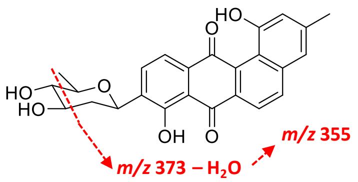

Dehydroxyaquayamycin (**22**)
$C_{25}H_{22}O_7$
$[M+H]^+$ Calcd.: 435.1438
$[M+H]^+$ Obs.: 435.1457
Δ = - 4.3 ppm

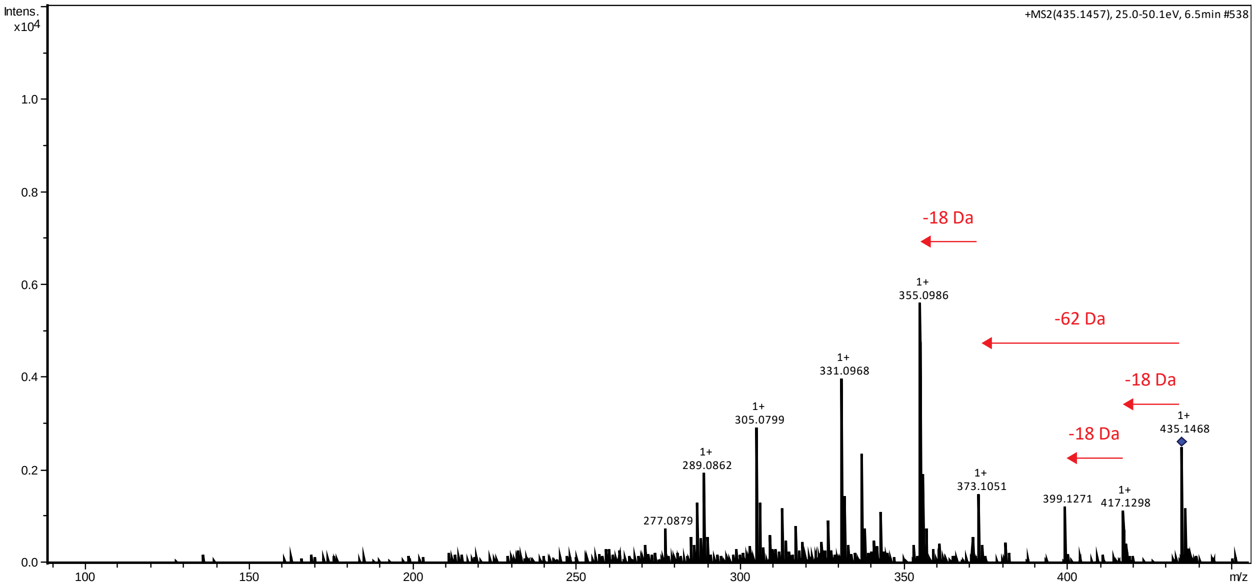

Figure S76. MS/MS spectrum of detected dehydroxyaquayamycin (**22**) from the molecular network of interactions amongst endophytic microorganisms from *L. ericoides*.

Although nodes from co-cultures are red and nodes from mono- and co-cultures of *S. mobaraensis* RLe3 are in aquamarine, the compound **22**, corresponding to dehydroxyaquayamycin, was only found in low abundance which resulted in fragment spectra with additional peaks from chemical noise. Consequently, a separate cluster of two nodes was created by the algorithm.

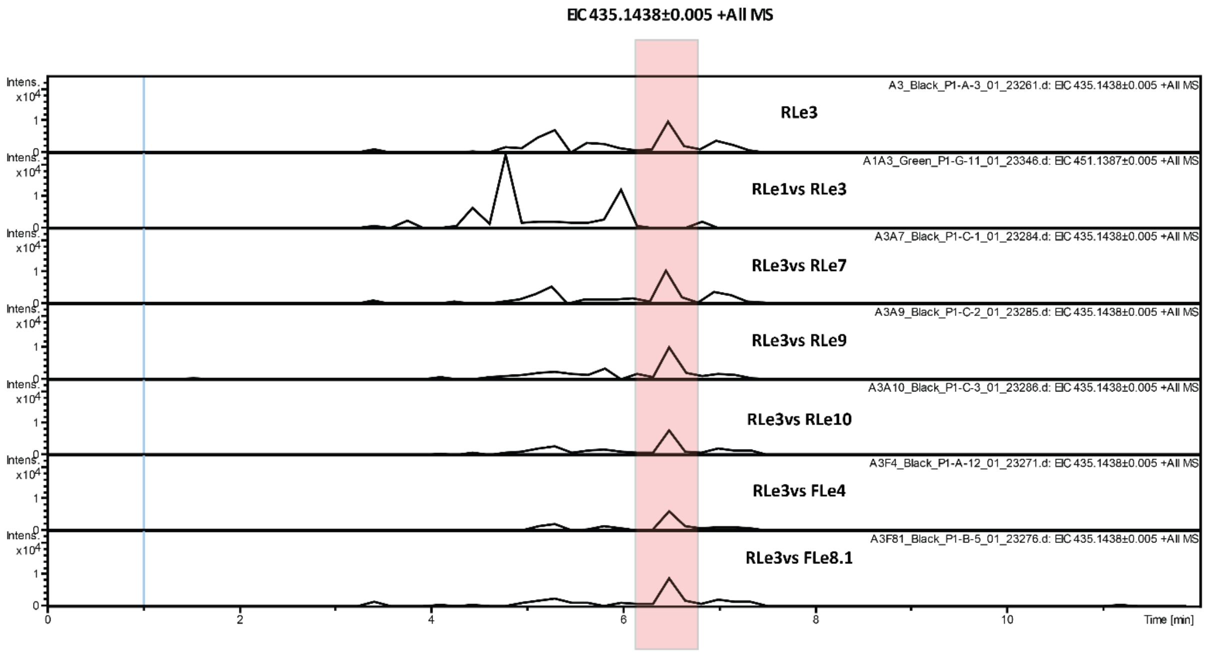

Figure S77. Extracted ion chromatogram comparison for compound (**22**)
Dehydroxyaquayamycin (**22**), *m/z* 435, was produced by *S. mobaraensis* RLe3 and detected in mono- and co-cultures. The EIC are representative from four replicates.

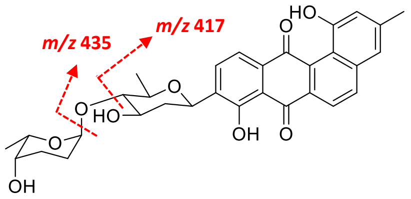

Marangucycline A_2 (**23**)
$C_{31}H_{32}O_9$
$[M+H]^+$ Calcd.: 549.2119
$[M+H]^+$ Obs.: 549.2115
Δ = 0.7 ppm

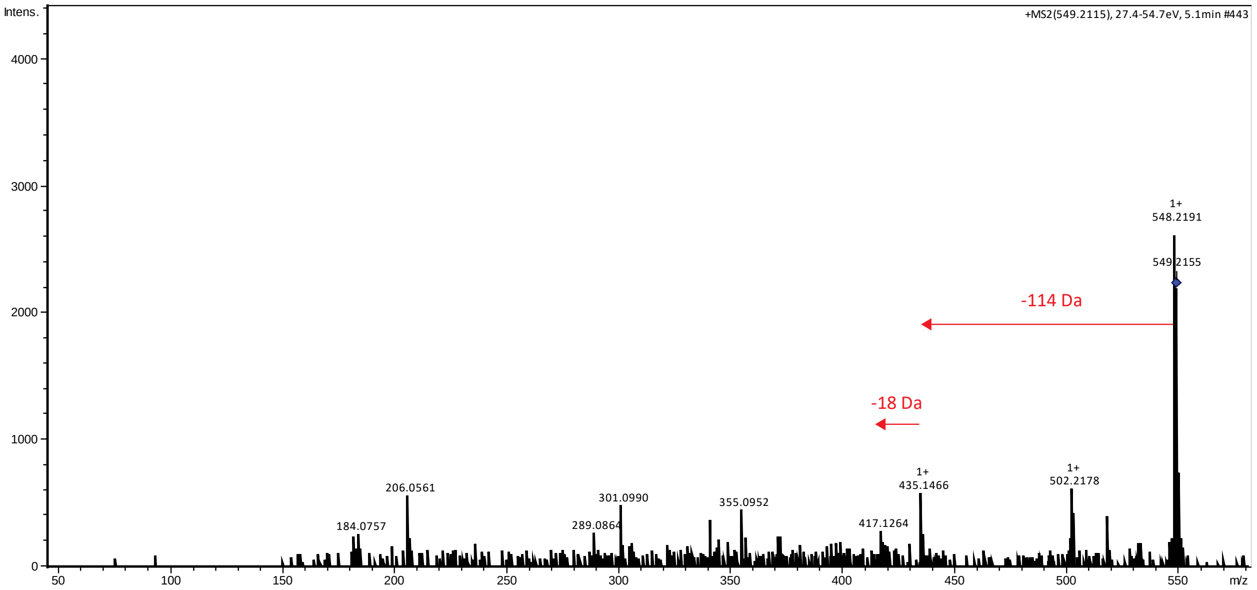

Figure S78. MS/MS spectrum of detected marangucycline A_2 (**23**) from the molecular network of interactions amongst endophytic microorganisms from *L. ericoides*.
The node of *m/z* 549, corresponding to compound **23**, was detected from samples of mono-cultures of *S. mobaraensis* RLe3 and in co-cultures of *S. mobaraensis* RLe3 with *K. cystarginea* RLe10.

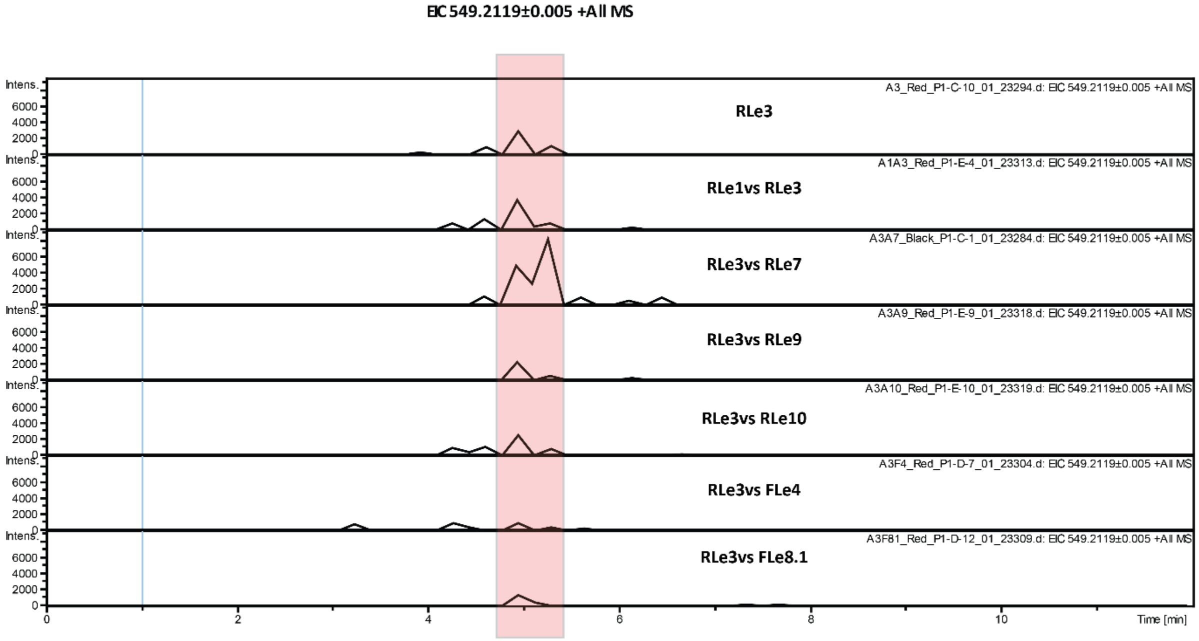

Figure S79. Extracted ion chromatogram comparison for compound (**23**)
Marangucycline A_2 (**23**), *m/z* 549, was produced by *S. mobaraensis* RLe3 and detected in mono- and co-cultures. The EIC are representative from four replicates.

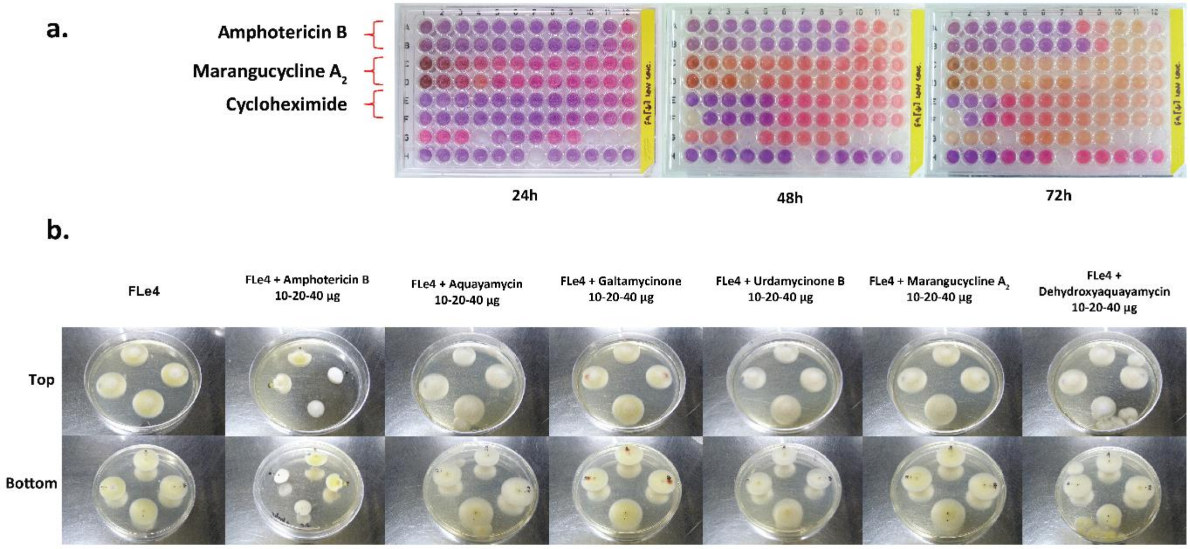

Figure S80. Biological assay of purified compounds against *Coniochaeta* sp. FLe4.
Coniochaeta sp. FLe4 was tested against pure compounds in: **a.** MIC including AmpB, marangucycline A_2 and cycloheximide (as antifungal control). *Coniochaeta* sp. FLe4 mycelia suspension (E) was obtained from a three-days culture of the fungus in ISP-2 reactivated as previously described. Each well contained 20 µL of resazurin (R) as indicator of cell viability. Controls for substances H1-2 (amphotericin B, A2411 Sigma-Aldrich), H3-4 (compound 6) and H5-6 (cycloheximide, C7698 Sigma-Aldrich), for the fungus growth (G1-G3), for DMSO with fungus (G5-G9), sterility of DMSO (H8-H9), and sterility of ISP-2 (H10 - H12) were also included; **b**. Diffusion agar assay including amphotericin B (compound **1**), aquayamycin (compound **19**), urdamycinone B (compound **20**), galtamycinone (compound **21**), dehydroxyaquayamycin (compound **22**) and marangucycline A_2 (compound **23**) at 10, 20 and 40 µg applied directly on the agar in front to the fungal colony.

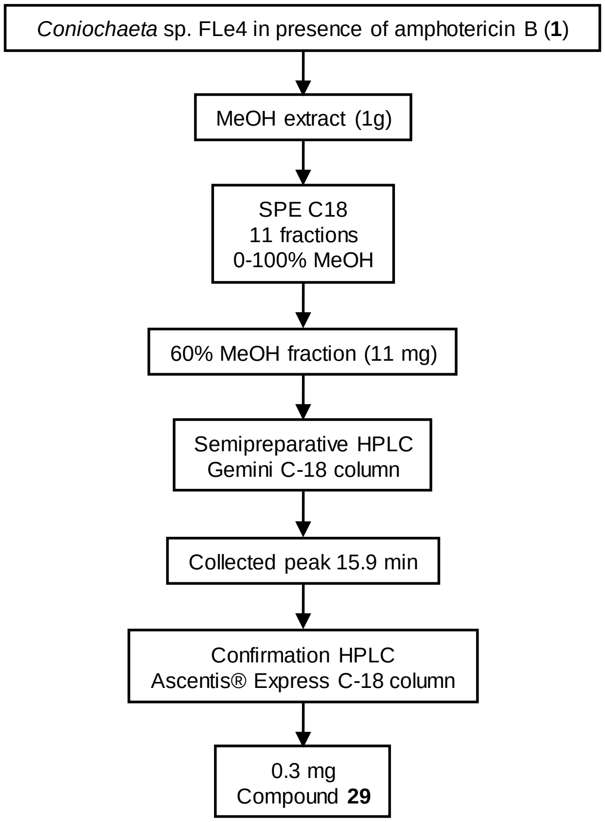

Figure S81. Purification workflow for compound (**29**)

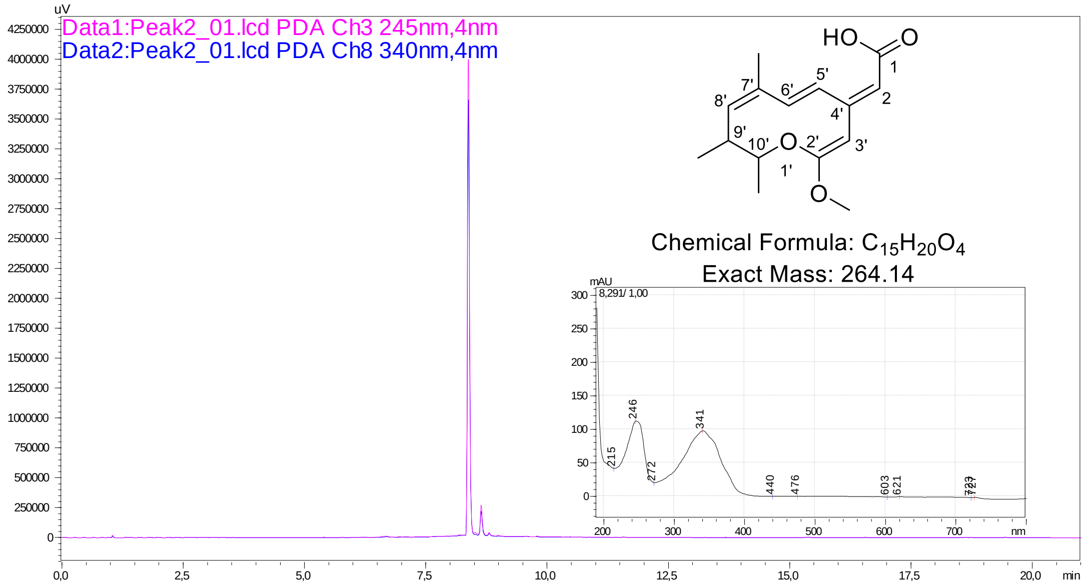

Figure S82. HPLC-DAD of purified peak corresponding to compound (**29**)

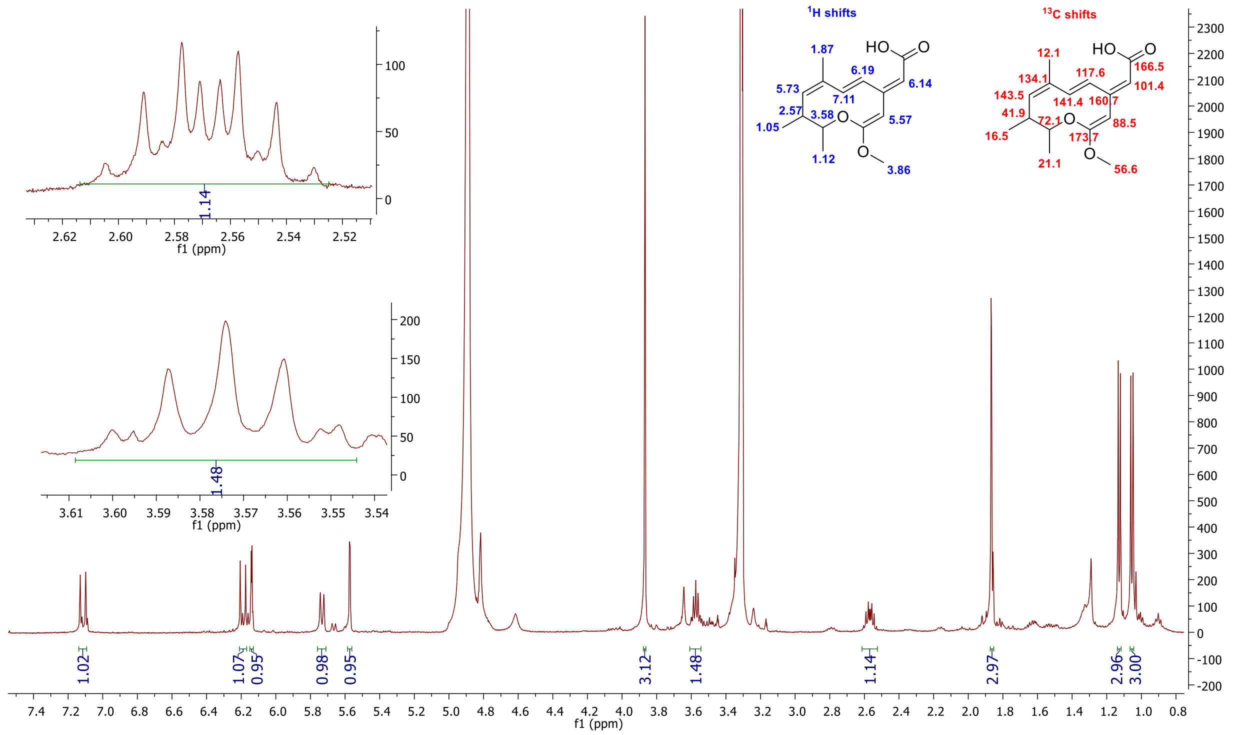

Figure S83. ^{1}H NMR (500 MHz, MeOH-*d*4) spectrum of compound (**29**)

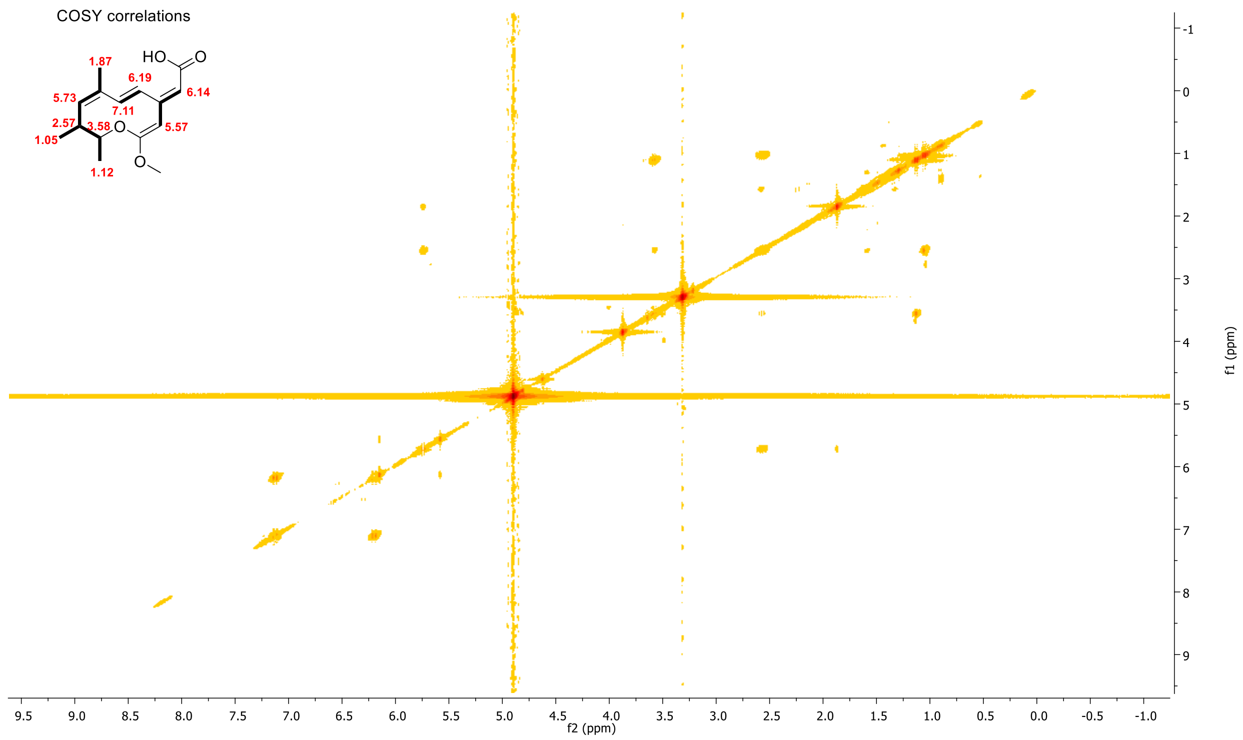

Figure S84. COSY (500 MHz, MeOH-*d*4) spectrum of compound (**29**)

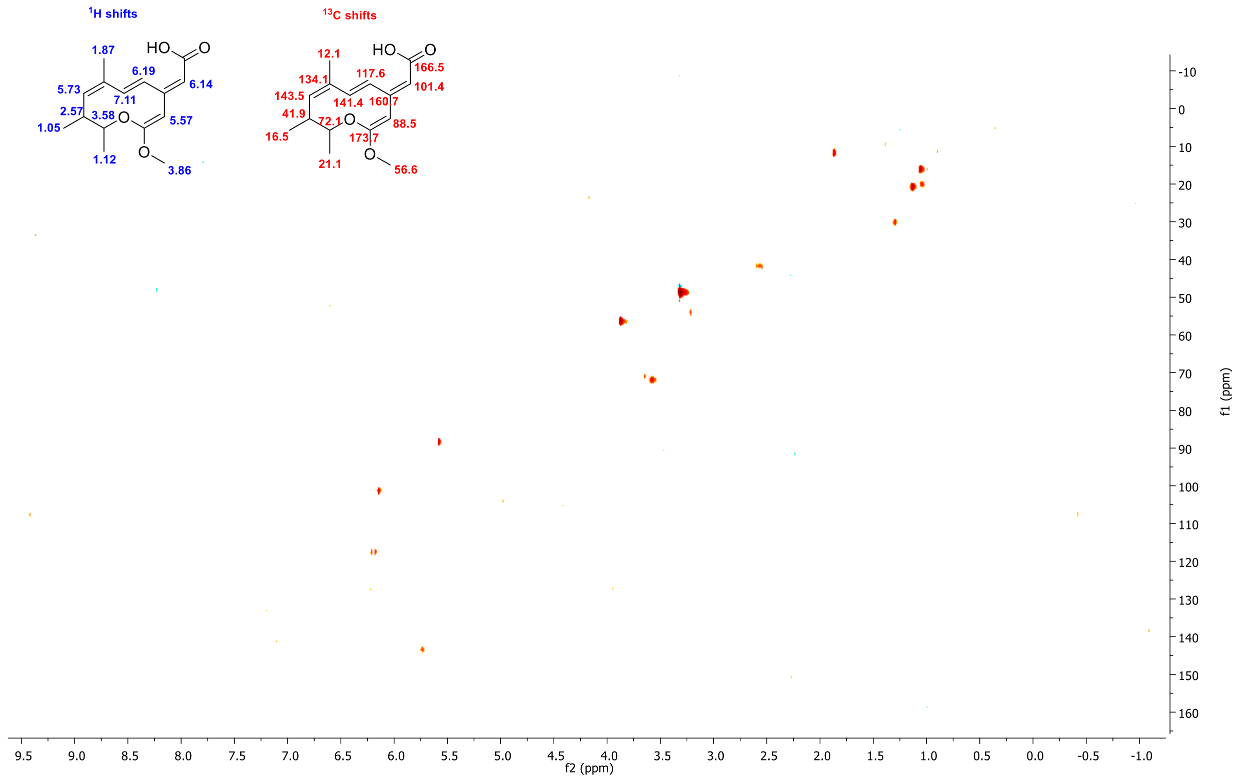

Figure S85. *g*HSQC (500 MHz, MeOH-*d*4) spectrum of compound (**29**)

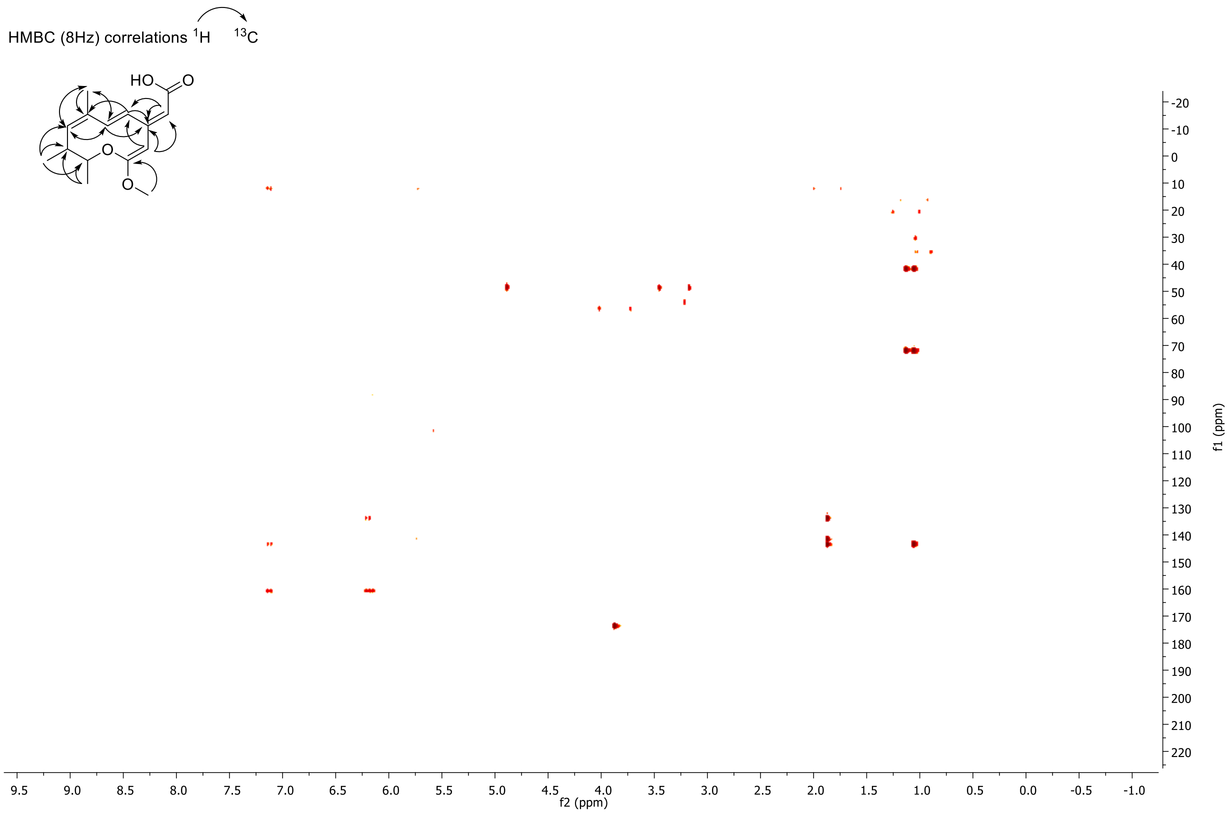

Figure S86. *g*HMBC (500 MHz, MeOH-*d*4) spectrum of compound (**29**)

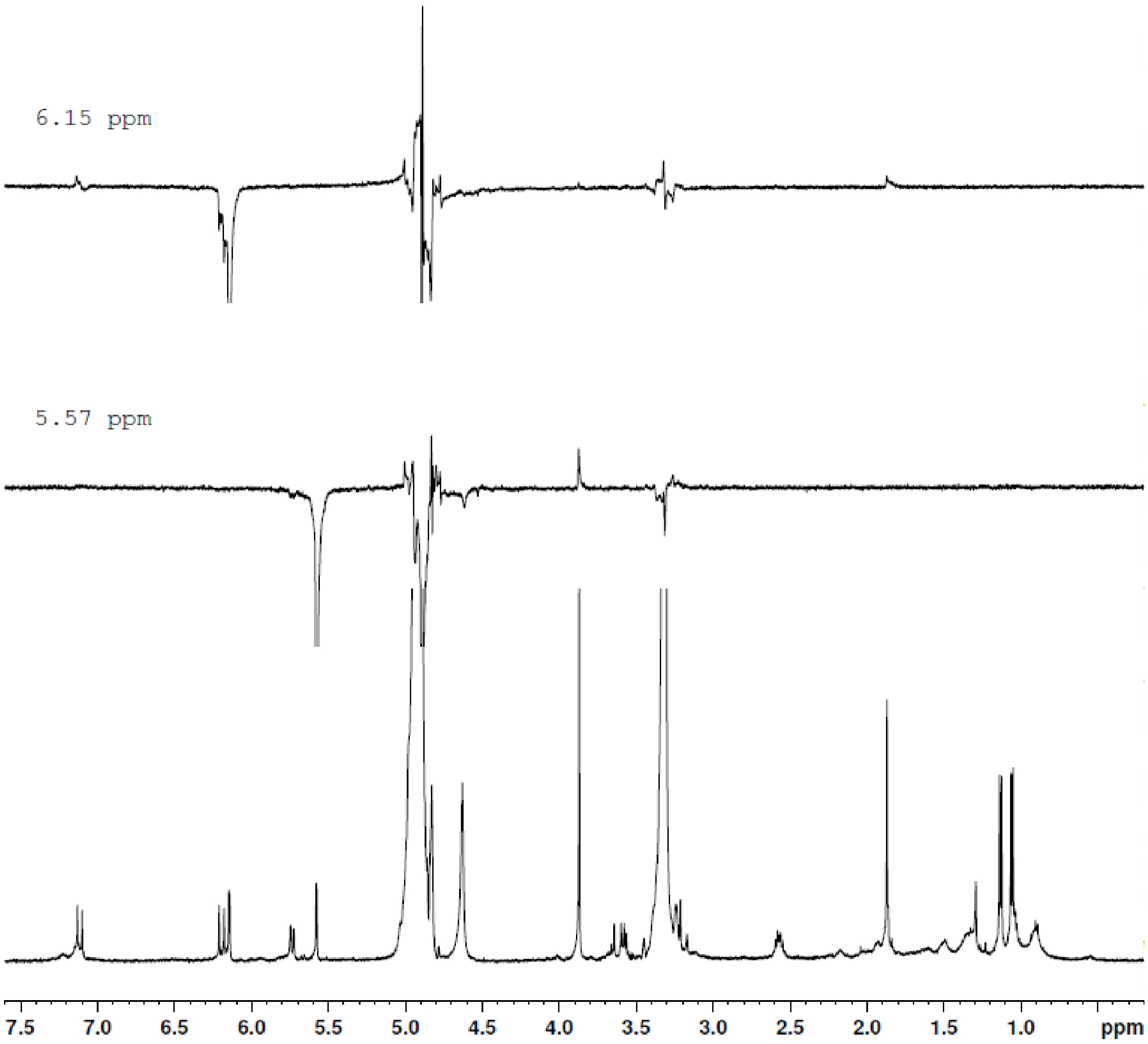

Figure S87. NOE DIFF (500 MHz, MeOH-*d*4) spectrum of compound (**29**)

NOESY correlations

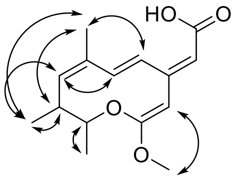

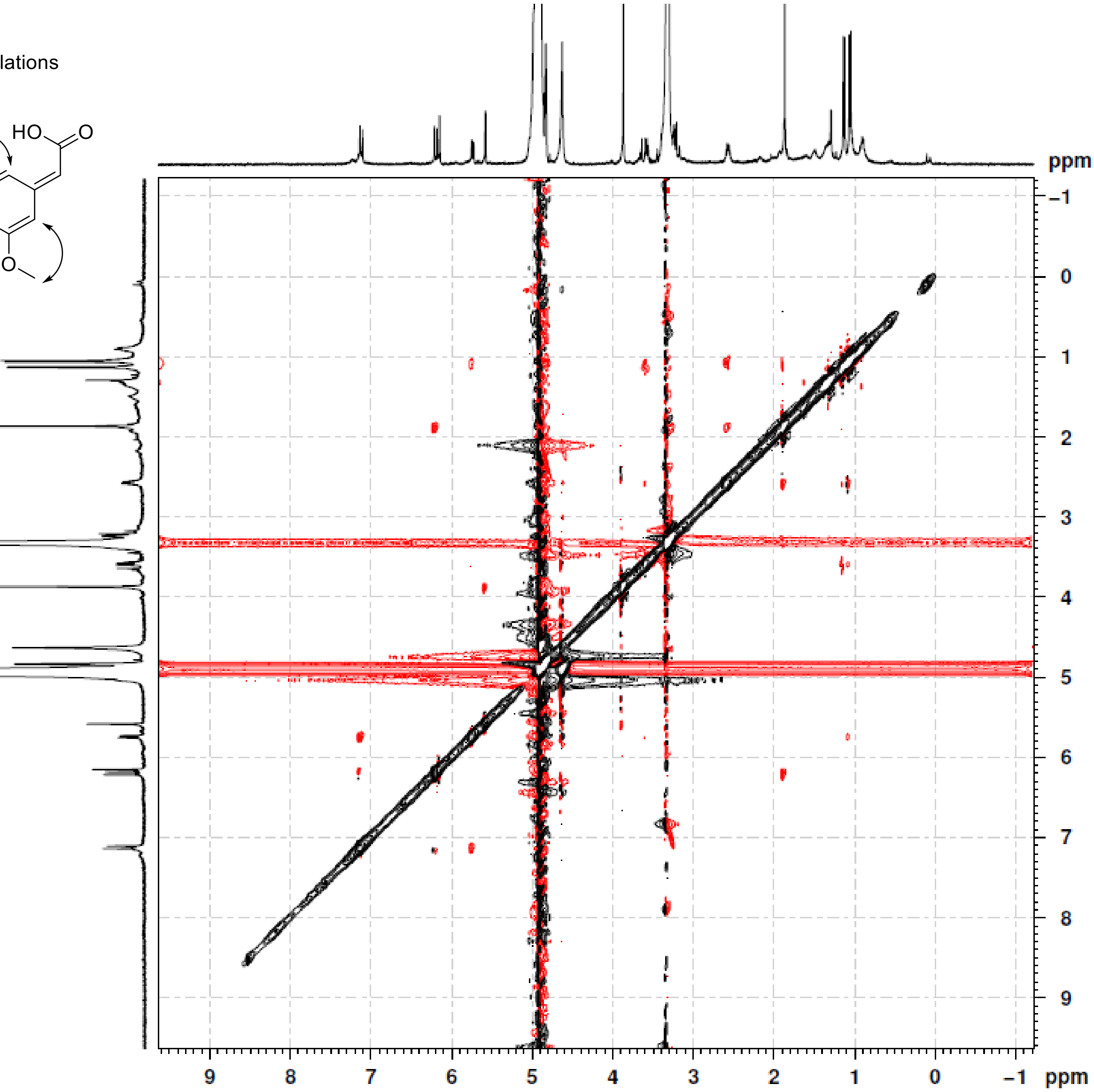

Figure S88. NOESY (500 MHz, MeOH-*d*4) spectrum of compound (**29**)

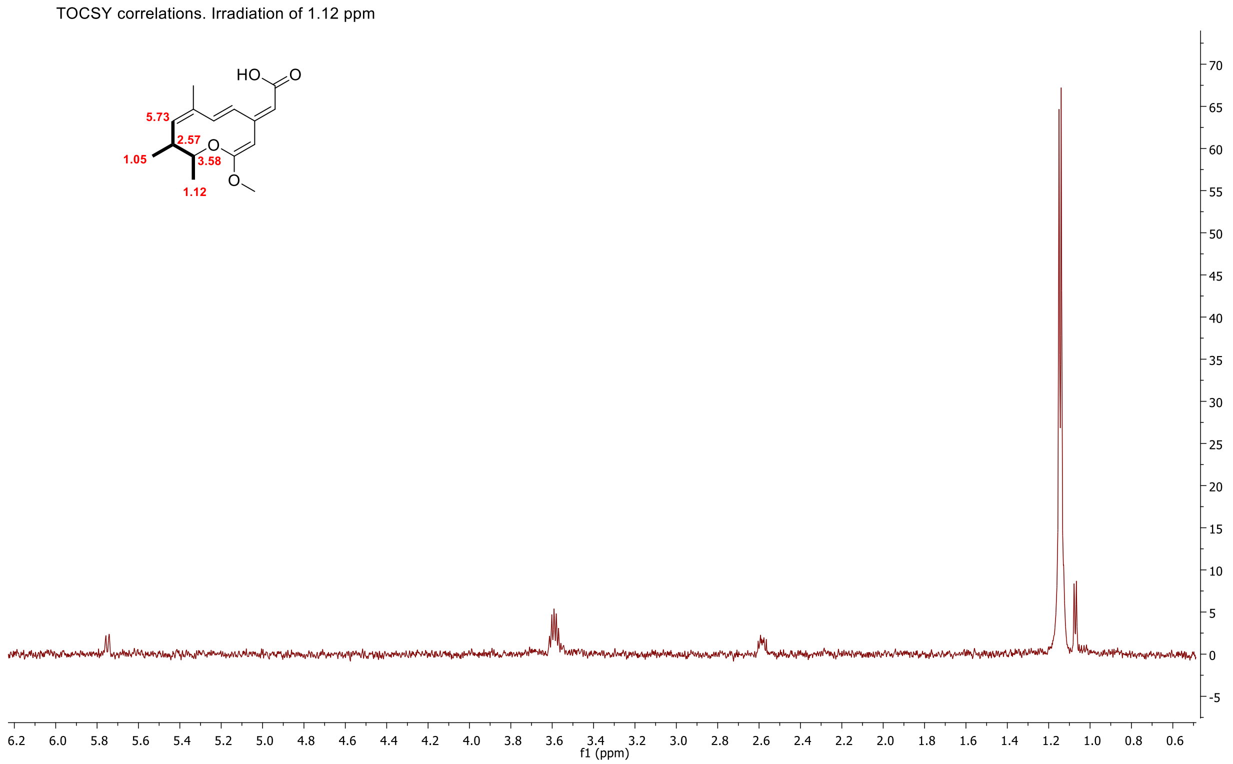

Figure S89. TOCSY 1D 1.12 ppm (600 MHz, MeOH-*d*4) spectrum of compound (**29**)

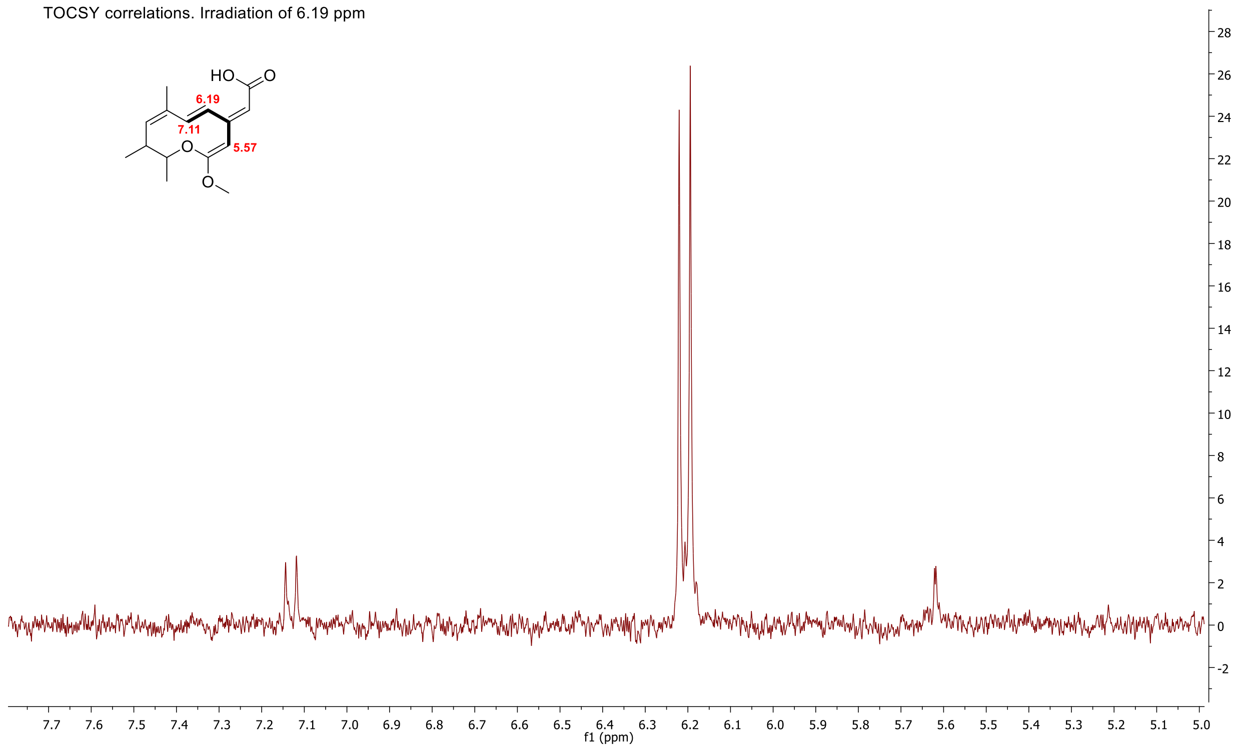

Figure S90. TOCSY 1D 6.19 ppm (600 MHz, MeOH-d4) spectrum of compound (**29**)

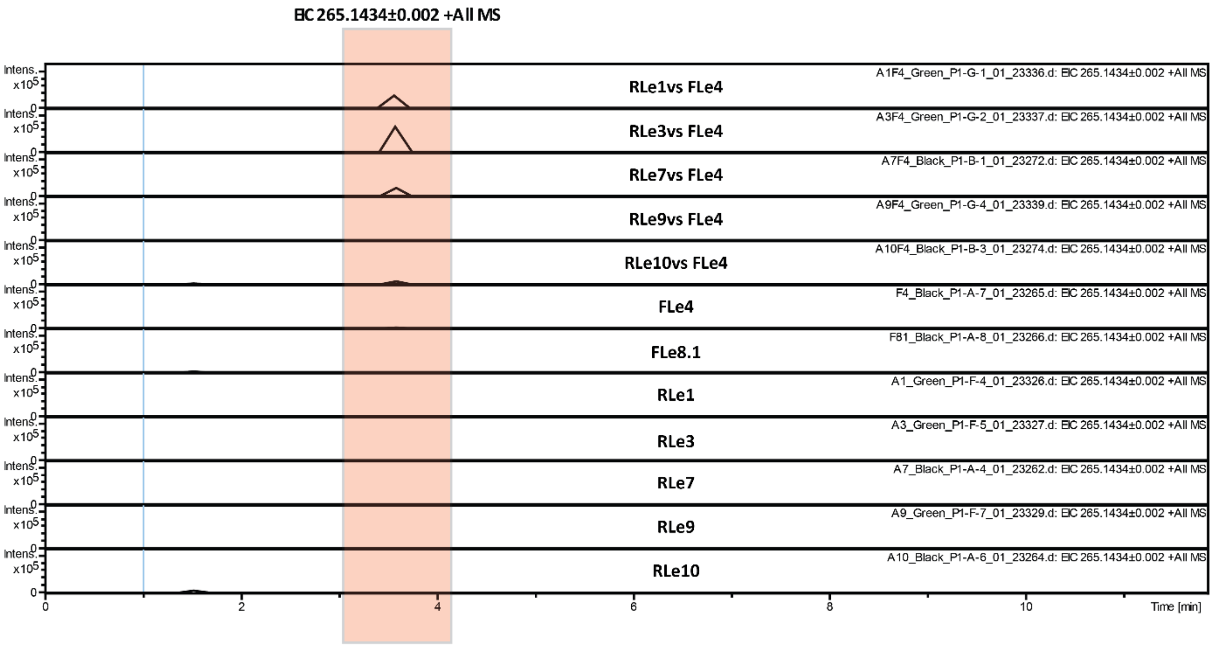

Figure S91. Comparison of extracted ion chromatograms (MS1) of *m/z* 265.1434±0.002 of mono- and co-cultures involving *Coniochaeta* sp. FLe4.

Specifically, the node of *m/z* 265 corresponding to compound **29** (calcd for $C_{15}H_{21}O_4$, $[M+H]^+$, 265.1434) was detected from samples of co-cultures involving *Coniochaeta* sp. FLe4 with *S. cattleya* RLe1, *S. mobaraensis* RLe3, *S. albospinus* RLe7 and *K. cystarginea* RLe10. Comparison of extracted ion chromatograms (EIC) from mono-culture and co-cultures of *S. cattleya* RLe1, *S. mobaraensis* RLe3, *S. albospinus* RLe7, *Streptomyces* sp. RLe9, *K. cystarginea* RLe10, *Coniochaeta* sp. FLe4 and *C. boninense* FLe8.1 confirmed the absence of this compound in samples from mono-cultures of actinobacteria and fungi, as well as from co-cultures between *Streptomyces* sp. RLe9 and *Coniochaeta* sp. FLe4.

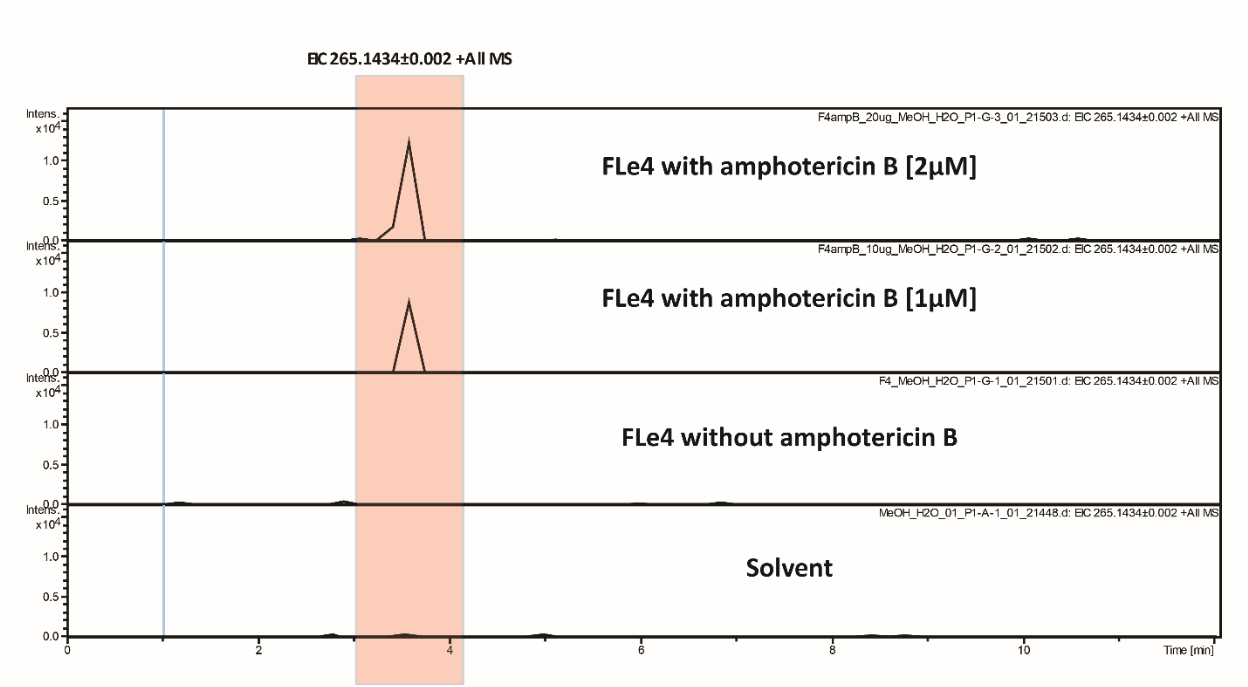

Figure S92. Comparison of extracted ion chromatograms (MS1) of *m/z* 265.1434±0.002 from samples of cultures involving *Coniochaeta* sp. FLe4 in presence and absence of amphotericin B. Specifically, the node of *m/z* 265 corresponding to compound **29** (calcd for $C_{15}H_{21}O_4$, $[M+H]^+$, 265.1434) was detected from samples of *Coniochaeta* sp. FLe 4 in presence of amphotericin B (compound **1**) at 1 and 2 μM. Isolation of compound **29** was obtained from large-scale cultures of *Coniochaeta* sp. FLe4 in presence of amphotericin B (compound **1**) at 2 μM.

Table S1. NMR Spectroscopy Data (500 MHz, MeOH-d_4) for compound **19** consistent with reported compound from literature.[1]

Position	δ_C, type	δ_H, mult, (J in Hz)	^{1}H-^{1}H COSY	^{1}H-^{13}C HMBC (H→C)
1	207.0, C	-	-	-
2a	52.9, CH_2	2.67, d (12.5)	2.03; 2.82	44.2; 77.5; 207.0
2b	52.9, CH_2	2.82, d (12.5)	2.67	29.7; 77.5; 207.0
3	77.5, C	-	-	-
4	44.2, CH_2	2.03, br s	2.67	52.9; 77.5; 81.8
4a	81.8, C	-	-	-
5	146.1, CH	6.40, d (8.9)	6.87	-
6	117.9, CH	6.87, d (8.9)	6.40	-
6a	131.7, C	-	-	-
7	ND	-	-	-
7a	ND	-	-	-
8	158.9, C	-	-	-
9	139.2, C	-	-	-
10	134.1, CH	7.86, d (7.6)	7.59	131.9; 158.9
11	119.9, CH	7.59, d (7.6)	7.86	-
11a	131.9, C	-	-	-
12	ND	-	-	-
12a	ND	-	-	-
12b	ND	-	-	-
13	29.7, CH_3	1.24, s	-	44.2; 52.9; 77.5; 207.0
2’	72.2, CH	4.89 (overlap with solvent peak)	1.37	139.2 (weak);
3’	40.7, CH_2	2.42, dd (12.5, 4.3) / 1.37, m (overlap with H-13)	1.37; 3.69 / 2.42; 3.69; 4.89	73.4; 78.4 / 72.2; 73.4; 139.2
4’	73.4, CH	3.69, m	1.37; 2.42; 3.03	78.4
5’	78.4, CH	3.03, t (9.0)	3.43; 3.69	18.1; 73.4; 77.4
6’	77.4, CH	3.43, td (12.5; 9.0)	1.37, 3.03	18.1; 78.4; 73.4
7’	18.1, CH_3	1.37, d (6.1)	3.43	78.4

Table S2. NMR Spectroscopy Data (500 MHz, Acetone-d_6) for compound **20** consistent with reported data from literature.[2]

	Compound 3 (acetone *d*6)				Urdamycinone B in acetone *d*6
Position	δ_C, type	δ_H, mult, (*J* in Hz)	^{1}H-^{1}H COSY	^{1}H-^{13}C HMBC (H→C)	δ_H, mult, (*J* in Hz)
1	197.3, C	-	-	-	-
2	53.8, CH_2	3.06 (overlapping with H-5') / 2.87 (overlapping with solvent peak H_2O)	2.87	29.7; 44.2; 73.5; 197.3 / 29.7; 44.2; 73.5; 137.1; 197.3	2-Hax 3.33, d (17.0) / 2-Heq 3.21, dd (17.0, 1.0)
3	73.5, C	-	-	-	-
4	44.2, CH_2	3.20, d (16.8) / 3.31, d (16.8)	3.31 / 3.20	29.7; 53.8; 73.5; 134.7; 137.1; 150.3 / 29.7; 53.8; 73.5; 134.7; 137.1; 150.3	4-Hax 3.10, d (14.5) / 4-Heq 2.90, dd (14.5, 1.5)
4a	150.3, C	-	-	-	-
5	134.7, CH	7.73, d (8.0)	8.29	44.2; 134.5; 137.2	7.61, d (8.0)
6	129.2, CH	8.29, d (8.0)	7.73	137.2; 150.3; 189.4	7.95, d (8.0)
6a	137.2, C	-	-	-	-
7	189.4, C	-	-	-	-
7a	116.3, C	-	-	-	-
8	159.0, C	-	-	-	-
9	138.1, C	-	-	-	-
10	134.5, CH	7.93, d (7.8)	7.61	71.4; 135.4; 159.0	7.76, d (8.0)
11	119.6, CH	7.61, d (7.8)	7.93	116.3; 138.1; 183.8	8.31, d (8.0)
11a	135.4, C	-	-	-	-
12	183.8, C	-	-	-	-
12a	134.5, C	-	-	-	-
12b	137.1, C	-	-	-	-
13	29.7, CH_3	1.48, s	-	44.2; 53.8; 73.5; 197.3	1.49, s
2'	71.4, CH	4.90, d (11.2)	1.41	138.1	4.92, dd (11.5, 1.5)
3'	40.5, CH_2	1.41, q (11.3) / 2.43, ddd (12.7, 5.0, 2.0)	-	73.0; 138.1 / 73.0; 78.5	3'-Hax 1.23, ddd (13.0, 11.5, 9.5)* / 3'-Heq 2.46, ddd (13.0, 5.0, 2.0)
4'	73.0, CH	3.73, m	1.41; 3.08	-	3.76, ddd (12.0, 9.5, 5.0)
5'	78.5, CH	3.08 (d, *J* = 6.0 Hz, overlapping with H-2)	3.47; 3.73	17.9; 77.0	3.11, dd (8.5, 8.0)
6'	77.0, CH	3.47, m	1.35; 3.08	-	3.50, dd (9.5, 6.0)
7'	17.9, CH_3	1.35, d (5.8)	3.47	77.0	1.37, d (6.0)

*Partially obscured in the reported paper,[2] which is consistent with the observed overlapping signal between 1.35-1.48 ppm in our data.

Table S3. NMR Spectroscopy Data (500 MHz, MeOH-d_4) for compound **21** consistent with reported data from literature.[3]

	Compound 4				Galtamycinone (in pyridine-*d*5)*
**Position**	**δ_C, type**	**δ_H, mult, (*J* in Hz)**	**^{1}H-^{1}H COSY**	**^{1}H-^{13}C HMBC (H→C)**	**δ_C, type**
1	157.3, C	-	-	-	157.7, C
2	116.6, CH	6.88, s	2.44; 7.62	21.8; 115.5; 125.9	117.1, CH
3	143.1, C	-	-	-	142.3, C
4	115.5, CH	7.62, s	2.44; 6.88	116.6; 125.9; 164.1	115.5, CH
4a	ND	-	-	-	129.9, C
5	164.1, C	-	-	-	164.0, C
5a	110.2, C	-	-	-	109.9, C
6	188.4, C	-	-	-	187.0, C
6a	134.3, C	-	-	-	133.3, C
7	119.4, CH	7.81, d (7.9)	-	117.7; 138.6; 188.4	118.9, CH
8	133.7, CH	7.86, d (7.9)	-	134.3; 160.2	133.4, CH
9	138.6, C	-	-	-	138.6, C
10	160.2, C	-	-	-	159.6, C
10a	117.7, C	-	-	-	116.9, C
11	189.1, C	-	-	-	188.4, C
11a	130.0, C	-	-	-	126.4, C
12	118.2, C	8.54, s	-	110.2; 130.0; 157.3; 189.1	118.0, C
12a	125.9, C	-	-	-	125.9, C
13	21.8, CH_3	2.44, s	6.88; 7.62	116.6; 143.1	22.2, CH_3
1’	72.2, CH	4.89 (solvent overlap)	-	138.6 (weak)	72.2, CH
2’	40.8, CH_2	2.48 dd (4.1, 12.5) / 1.42, m (overlaps with H-6’)	1.42; 3.72 / 2.48; 3.72; 4.89	73.4 / 73.4	41.1, CH_2
3’	73.4, CH	3.72, m	1.42; 2.48; 3.06	-	73.5, CH
4’	78.5, CH	3.06, t (9.0)	3.47; 3.72	73.4	78.7, CH
5’	77.5, CH	3.47, m	1.41; 3.06	-	77.5, CH
6’	18.1, CH_3	1.41, d (5.9)	3.47	77.5	19.0, CH_3

ND=not detected; *NMR data from ref.[3]

Table S4. NMR Spectroscopy Data (500 MHz, $CDCl_3$) for compound **22**, consistent with reported data from literature.[4]

	Compound 5				Dehydroxyaquayamycin (in acetone-*d*6)*	
**Position**	**δ_C, type**	**δ_H, mult, (*J* in Hz)**	**^1H-^1H COSY**	**^1H-^{13}C HMBC (H→C)**	**δ_C, type**	**δ_H, mult, (*J* in Hz)**
1	ND	-	-	-	155.5, C	-
2	120.3, CH	7.16, s	-	-	117.6, CH	6.53, s
3	142.6, C	-	-	-	147.8, C	-
4	120.9, CH	7.28, s	-	120.6	NR	6.91, s
4a	135.1, C	-	-	-	135.0, C	-
5	ND	8.16, d (8.6)	8.34	135.1; 120.6	135.7, CH	7.70, d (8.58)
6	121.8, CH	8.34, d (8.6)	8.16	-	120.2/121.8, CH**	7.70, d (8.58)
6a	ND	-	-	-	133.7, C	-
7	ND	-	-	-	187.0, C	-
7a	138.0, C	-	-	-	134.7, C	-
8	158.0, C	-	-	-	157.3, C	-
9	ND	-	-	-	137.5, C	-
10	133.5, CH	7.93, d (8.1)	-	-	133.6, CH	7.39, d (7.82)
11	120.1, CH	7.89, d (8.1)	-	-	119.8, CH	7.20, d (7.82)
11a	ND	-	-	-	114.8, C	-
12	ND	-	-	-	188.4, C	-
12a	ND	-	-	-	138.9, C	-
12b	120.6, C	-	-	-	119.1, C	-
13	20.9, CH_3	2.50, s	-	142.6; 120.9	22.5, CH_3	1.99, s
1-OH	-	11.40, s	-	120.6	-	10.38, s
8-OH	-	12.67, s	-	-	-	12.08, s
1’	-	-	-	-	-	-
2’	71.7, CH	4.96, d (11.2)	1.49	-	71.1, CH	4.35, d (11.32)
3’	39.1, CH_2	1.49 (hidden) / 2.54, dd (12.0; 4.0)	2.54; 3.87; 4.96 / 1.49; 3.87	-	NR	3’-Ha: 0.80, d (12.6) / 3’-Hb: 1.81, ddd (12.60, 11.32, 3.99)
4’	73.1, CH	3.87, m	3.24; 2.54; 1.49	-	72.2, CH	3.08-3.15, m
4’-OH	-	ND	-	-	-	4.53, d (4.63)
5’	77.9, CH	3.24, t (8.6)	3.55; 3.87	-	77.5, CH	2.45, dd (8.85, 5.03)
5’-OH	-	ND	-	-	-	4.62, d (5.15)
6’	75.7, CH	3.55, dd (8.6; 6.0)	3.24; 1.43	-	76.6, CH	3.00-3.06, m
7’	18.2, CH_3	1.43, d (6.0)	3.55	75.7; 77.9	18.9, CH_3	0.82, d (6.05)

ND=not detected; NR=not reported, *NMR data reported from DMSO-*d6* in the main paper (experimental section), but spectra in supplementary data, Figures 51S-54S is reported in acetone-d6,[4] **Two values reported for C-6.

Table S5. NMR Spectroscopy Data (500 MHz, $CDCl_3$) for marangucycline A[5] and compound **23**

	Marangucycline A_2 (6)					Marangucycline A	
**Position**	**δ_C, type**	**δ_H, mult, (*J* in Hz)**	**^1H-^1H COSY**	**^1H-^{13}C HMBC (H→C)**	**NOESY**	**δ_C, type**	**δ_H, mult, (*J* in Hz)**
1	155.4, C	-	-	-	-	155.6, C	-
2	120.0, CH	7.16, s	-	121.0; 21.2	-	120.2, CH	7.12, s
3	142.1, C	-	-	-	-	142.1, C	-
4	121.0, CH	7.27, s	2.50	137.6; 120.1; 21.2	-	121.5, CH	7.23, s
4a	134.9, C	-	-	-	-	132.6, C	-
5	137.5, CH	8.15, d (8.5)	8.33	121.6; 134.9	-	137.7, CH	8.11, d (8.5)
6	121.6, CH	8.33, d (8.5)	8.15	132.6; 139.4; 188.3	-	122.0, CH	8.29, d (8.5)
6a	132.6, C	-	-	-	-	135.0, C	-
7	188.3, C	-	-	-	-	188.3, C	-
7a	114.1, C	-	-	-	-	114.2, C	-
8	158.1, C	-	-	-	-	158.2, C	-
9	138.5, C	-	-	-	-	138.6, C	-
10	133.5, CH	7.92, d (8.0)	-	70.9; 133.5; 158.1	-	133.6, CH	7.90, d (8.0)
11	121.1, CH	7.90, d (8.0)	-	114.1; 138.5; 189.5	-	121.3, CH	7.86, d (8.0)
11a	133.5, C*	-	-	-	-	133.6, C	-
12	189.5, C	-	-	-	-	189.6, C	-
12a	139.4, C	-	-	-	-	139.3, C	-
12b	120.1, C	-	-	-	-	120.2, C	-
13	21.2, CH_3	2.50, s	7.27	121.0; 142.1	-	21.4, CH_3	2.48, s
1-OH	-	11.42, s	-	120.1; 155.4	-	-	11.43, s
8-OH	-	12.65, s	-	114.1; 138.5; 158.1	-	-	12.62, s
1'	70.9, CH	4.94, d (10.0)	1.46; 2.57	38.4; 71.4; 138.5	3.84; 3.57	71.3, CH	4.90, d (11.2)
2'	38.4, CH_2	1.46, m; 2.56, dd (13.0, 4.6)	2.56, 3.84, 4.94; 1.46, 3.84, 4.94	71.4; 71.4, 88.9	-	38.8, CH_2	2.57, m; 1.46, m
3'	71.3, CH	3.84, m	1.46, 2.57, 3.09	-	4.94	71.5, CH	3.87, m
4'	88.9, CH	3.09, t (8.6)	3.57, 3.84	18.2; 71.4; 74.4; 99.6	4.99	89.2, CH	3.07, t (6.5)
5'	74.4, CH	3.57, m	1.39, 3.09	18.2	4.94	74.7, CH	3.57, m
6'	18.2, CH_3	1.39, d (6.0)	3.57	74.5; 88.9	-	18.6, CH_3	1.38, d (6.0)
1"	99.6, CH	4.99, s	2.10, 1.66	88.9; 67.9; 24.1	3.09	98.9, CH	4.92, s
2"	24.1, CH_2	1.66, m; 2.10, m	1.66; 4.99	-	-	27.3, CH_2	1.93, m; 1.83, m
3"	25.3, CH_2	1.82, m; 2.04, m	2.10, 2.04;	-	-	30.1, CH_2	1.87, m; 1.25, m
4"	67.0, CH	3.69, br s	1.82	-	-	71.8, CH	3.36, td (10.0, 4.0)
5"	67.9, CH	4.25, q (6.5)	1.27	16.8; 67.0	-	71.7, CH	3.91, m
6"	16.8, CH_3	1.27, d (6.6)	4.25	67.9	-	18.0, CH_3	1.33, d (6.0)

Data consistent with β-D-olivose and L-Rhodinose[5,6] *Not observed, shift assumed from ref.[5]

Table S6. NMR Spectroscopy Data (500 MHz, MeOH-*d*4) for compound **29,** this study)

Position	δ_C, type	δ_H, mult, (*J* in Hz)	^1H-^1H COSY	^1H-^{13}C HMBC (H→C) (8Hz)	^1H-^{13}C HMBC (H→C) (4Hz)	NOESY	TOCSY1D
1	166.5, C	-	-	-	-	-	-
2	101.4, CH	6.14, d (2.2)	5.57	160.7; 117.6	88.5; 117.6; 160.7; 173.7		
2’	173.7, C	-	-	-	-	-	-
3’	88.5, CH	5.57, d (2.2)	6.14	101.4	166.5; 173.7	3.86	
4’	160.7, C	-	-	-	-	-	-
5’	117.6, CH	6.19, d (15.7)	7.11	134.0; 160.7	101.4; 134.0; 141.4; 166.5		
6’	141.4, CH	7.11, d (15.7)	6.19	12.1; 143.5; 160.7	12.1; 117.6; 134.0; 143.5; 160.7	5.73	5.57
7’	134.1, C	-	-	-	-	-	-
8’	143.5, CH	5.73, d (10.3)	1.87; 2.57	12.1; 141.4	-	1.05; 7.11	
9’	41.9, CH	2.57, dquint (10.3, 6.7)	1.05; 3.58; 5.73	-	16.5; 21.1; 72.1; 134.1; 143.5	1.05; 1.87	
10’	72.1, CH	3.58, br quint (6.6, 6.3)	1.12; 2.57	-	16.5; 41.9; 143.5		
7’-Me	12.1, CH_3	1.87, s	5.73	134.0; 141.4; 143.5	134.0; 141.4; 143.5	1.05; 2.57; 6.19	
9’-Me	16.5, CH_3	1.05, d (6.7)	2.57	41.9; 72.1; 143.5	41.9; 72.1; 143.5	2.57	
10’-Me	21.1, CH_3	1.12, d (6.3)	3.58	41.9; 72.1	41.9; 72.1	3.58	5.73; 3.58; 2.57; 1.05
2’-OMe	56.6, CH_3	3.86, s	-	173.7	173.7		

References

1. Sezaki M, Kondo S, Maeda K, Umezawa H, Ohno M (1970). Structure of Aquayamycin. *Tetrahedron* **26:** 5171-5190.

2. Rohr J, Zeeck A (1987). Metabolic products of microorganisms. 240. Urdamycins, new angucycline antibiotics from *Streptomyces fradiae*. 2. Structural studies of urdamycin B to urdamycin F. *Journal of Antibiotics* **40:** 459-467.

3. Stroch K, Zeeck A, Antal N, Fiedler HP (2005). Retymicin, galtamycin B, saquayamycin Z and ribofuranosyllumichrome, novel secondary metabolites from Micromonospora sp Tu 6368 - II. Structure elucidation. *Journal of Antibiotics* **58:** 103-110.

4. Supong K, Thawai C, Suwanborirux K, Choowong W, Supothina S, Pittayakhajonwut P (2012). Antimalarial and antitubercular C-glycosylated benz alpha anthraquinones from the marine-derived Streptomyces sp BCC45596. *Phytochemistry Letters* **5:** 651-656.

5. Song YX, Liu GF, Li J, Huang HB, Zhang X, Zhang H *et al* (2015). Cytotoxic and Antibacterial Angucycline- and Prodigiosin- Analogues from the Deep-Sea Derived Streptomyces sp. SCSIO 11594. *Marine Drugs* **13:** 1304-1316.

6. Oki T, Kitamura I, Matsuzawa Y, Shibamoto N, Ogasawara T, Yoshimoto A *et al* (1979). Antitumor anthracycline antibiotics, aclacinomycin a and analogues. II. Structural determination. . *The Journal of Antibiotics* **32:** 801-819.
